# Targeted Expression of miR-7 Operated by TTF-1 Promoter Inhibited the Growth of Human Lung Cancer through the NDUFA4 Pathway

**DOI:** 10.1016/j.omtn.2016.12.005

**Published:** 2016-12-31

**Authors:** Liangyu Lei, Chao Chen, Juanjuan Zhao, HaiRong Wang, Mengmeng Guo, Ya Zhou, Junming Luo, Jidong Zhang, Lin Xu

**Affiliations:** 1 Department of Immunology, Zunyi Medical College, Guizhou 563000, China; 2 Department of Medical Physics, Zunyi Medical College, Guizhou 563000, China

**Keywords:** miR-7, TTF-1, 95D cells, lung cancer, growth, NDUFA4

## Abstract

Targeted expression of gene technique is an important therapeutic strategy for lung cancer. MicroRNA-7 has been well documented as a promising tumor suppressor but never been test in specific gene-promoter-targeted expression in cancer gene therapy. Here, we first evaluated the efficacy of miR-7 expression operated by the promoter of TTF-1, a lineage-specific oncogene in lung cancer, in vitro using an eukaryotic vector of TTF-1-promoter-operated expression of miR-7 (termed as p-T-miR-7). Interestingly, using a nude mice model, the growth and metastasis of human lung cancer cells in vivo were significantly reduced in remote hypodermic injection of the p-T-miR-7 group, accompanied by increased expression of miR-7 and reduced transduction of the Akt and Erk pathway in situ. Mechanism aspect, global gene expression analysis showed that downregulation of NDUFA4, a novel target of miR-7, contributed to the effects of miR-7 expression operated by TTF-1 promoter on the growth and metastasis of human lung cancer cells, as well as altered transduction of the Akt and Erk pathway. Finally, there was no significant difference in weight or histopathology of other organs. These data provided a basis for development of novel modality of miRNA-based targeted expression therapy against clinical lung cancer.

## Introduction

Lung cancer is one of the most common causes of cancer death. The overall 5-year survival rates for surgical resection in lung cancer patients still remain poor. Thus, it is important to identify and characterize new molecular markers and genes to develop more targeted treatment strategies to improve the clinical outcome of lung cancer.^1^, ^2^, ^3^ Recent evidence showed that the technique of targeted gene expression, including specific gene-promoter-operating expression and distinct delivery systems, is a crucial strategy for gene therapy against various cancers including lung cancer.^4^, ^5^, ^6^, ^7^ And, microRNAs (miRNAs), which are endogenous 21- to 23-nt non-coding RNAs, have pointed to central regulatory roles in the development of lung cancer and emerged as important targets for gene therapy against clinical lung cancer.^8^, ^9^, ^10^, ^11^ This research suggested that targeted expression of distinct miRNA molecules might be a useful therapeutic strategy for lung cancer, which would be ultimately benefit to the outcome of clinical lung cancer patients.

MicroRNA-7 (miR-7), a unique member of miRNAs, played an important role in the progression of various tumors including lung cancer.^12^, ^13^, ^14^ For lung cancer, accumulating evidence suggested that miR-7 was an important regulator in the development of lung cancer through controlling the growth and invasion, as well as apoptosis, of lung cancer cells and emerged as a novel potential therapeutic target. For example, Xiong et al.^15^ found that overexpression of miR-7 suppressed NSCLC cells proliferation and migration in vitro, and reduced tumorigenicity in vivo. At the same time, Li et al.^16^ showed that restoration of miR-7 expression suppressed the tumorigenicity of lung cancer cells in vivo. Consistently, our previous work also showed that overexpression of miR-7 could reduce the growth and metastasis of human lung cancer cells in vivo and in vitro.^17^ Moreover, the reduced expression of miR-7 was associated with the sites mutation of its promoter region in lung cancer tissues, indicating that miR-7, an important tumor suppressor, could be used as candidate for targeted gene therapy against lung cancer.^18^, ^19^

Thyroid transcription factor (TTF)-1 is a member of the homeodomain-containing Nkx2 family of transcription factors. Recent evidence showed that TTF-1, as a lineage-specific oncogene, was dominantly expressed in lung cancer, but not other types of cancers, and its expression level was closely correlation with the prognosis of lung cancer patients.^20^, ^21^, ^22^, ^23^ These findings raise an interesting question whether promoter of TTF-1 gene might be an ideal candidate for therapeutic strategy of targeted expression of distinct genes in lung cancer, which remains to be elucidated. Therefore, in this present study, we first constructed an eukaryotic vector of promoter of TTF-1-gene-operating expression of miR-7 (termed as p-T-miR-7) and observed its effects on the growth and migration of human lung cancer cells in vitro. Moreover, we further evaluated the potential effect of the TTF-1-promoter-operating miR-7 expression on the growth and metastasis of human lung cancer cells in vivo. Of note, we found that overexpression of NDUFA4, a novel target molecule of miR-7, could abrogate the effect of TTF-1-promoter-operating miR-7 expression on the growth of lung cancer cells, accompanied with altered expression of phosphorylation of Akt and Erk. Thus, our work showed for the first time that TTF-1-promoter-operating miR-7 expression might be an ideal strategy for targeted expression of miR-7 in lung cancer, which provides preliminary experimental basis for targeted expression of distinct miRNAs in lung cancer and was helpful for the development of gene therapy against clinical lung cancer.

## Results

### TTF-1-Promoter-Operating miR-7 Expression Suppressed the Growth of Human Lung Cells In Vitro

**Figure 1:**
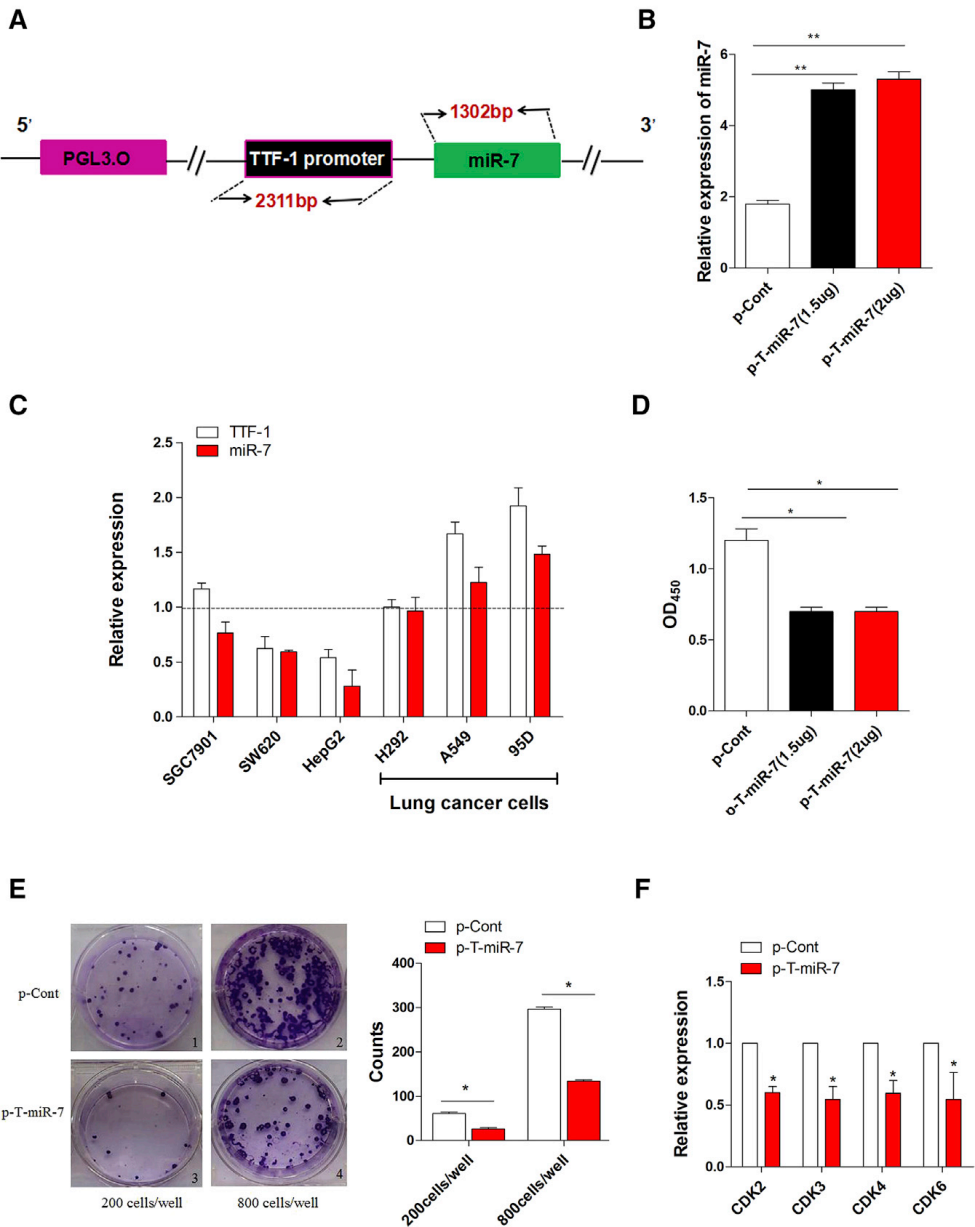
TTF-1-Promoter-Operating miR-7 Expression Inhibited the Growth of Human Lung Cancer Cells In Vitro (A) The schematic of an eukaryotic expression vector (termed as p-T-miR-7). (B) Human lung cancer cell line 95D cells were transiently transfected with p-T-miR-7 (1.5 or 2 μg) or p-Cont (2 μg) in vitro. 48 hr later, cells were collected, and the expression level of miR-7 was detected by real-time PCR assay. (C) The plasmid of p-T-miR-7 (2 μg) was transiently transfected into human lung cancer cell line 95D cells, A549 cells, NCI-H292 cells, gastric cancer cell line SGC901 cells, hepatic cancer cell line HepG2 cells, and colon cancer cell line SW620 cells, respectively. After 48 hr, the expression level of miR-7 and TTF-1 was analyzed by real-time PCR assay. The relative expression of miR-7 was normalized to the corresponding control group. (D) Human lung cancer cell line 95D cells were transiently transfected with p-T-miR-7 (2 μg) or p-Cont (2 μg) in vitro. 48 hr later, the proliferation of 95D cells was detected by CCK-8 assay. (E) The colony-formation assay was performed, and the colon numbers were calculated. (F) Human lung cancer cell line 95D cells were transiently transfected with p-T-miR-7 (2 μg) or p-Cont (2 μg) in vitro. 48 hr later, the expression of indicated cell-growth-related molecules was detected by real-time PCR assay. Representative data of three independent experiments are shown. *p < 0.05, **p < 0.01.

To investigate whether the TTF-1 promoter might be an ideal candidate for operating miR-7 expression in lung cancer, as shown in Figure 1A, we first amplified and inserted the sequence of both TTF-1 promoter and miR-7 into pGL3.0 basic vector and successfully constructed an eukaryotic expression vector, which could express miR-7 operated by TTF-1 promoter (termed as p-T-miR-7) (Figure S1). In order to test the efficiency of TTF-1-promoter-operating miR-7 expression, we then transiently transfected plasmid p-T-miR-7 into human lung cancer cell line 95D cells. Real-time PCR assay showed that the expression level of the miR-7 in p-T-miR-7 transfection group was unmistakably higher than that in the control group (Figure 1B; p < 0.05), indicating TTF-1 promoter could effectively operate the expression of miR-7 in lung cancer cells. Next, to observe the target efficiency, we further transfected the plasmid p-T-miR-7 into six different human cancer cell lines, including lung cancer cell line 95D cells, A549 cells, NCI-H292 cells, gastric cancer cell line SGC901 cells, hepatitic cancer cell line HepG2 cells, and colon cancer cell line SW620 cells, and then detected the expression level of miR-7 operated by TTF-1 promoter. Interestingly, we found that the expression level of miR-7 was higher in lung cancer cells, including 95D cells, A549 cells, and NCI-H292 cells, than in other types of tumor cells (Figure 1C; p < 0.05), indicating higher intrinsic activity of TTF-1 promoter in lung cancer cells. To verify this phenomenon, we also detected the expression of TTF-1 in these cancer cells, which also valuably reflected the intrinsic activity of TTF-1 promoter. Consistently, we found that the expression level of TTF-1 was also higher in lung cancer cells than that in other cancer cells, which was consistent with previous finding that TTF-1 was dominantly expressed in lung cancer cells, but not other types of cancer cells.^24^, ^25^, ^26^

Accumulating literatures have documented that miR-7, as a promising tumor suppressor, could regulate the biological behavior of human lung cancer cells.^1^, ^12^, ^13^ Moreover, our previous research works also showed that overexpression of miR-7 could inhibit the growth of human lung cancer cells.^17^, ^18^ Therefore, we further investigated whether expression of miR-7 operated by TTF-1 promoter could affect the growth of human lung cancer cells in vitro. As shown in Figure 1D, the proliferation of human lung cancer cell line 95D cells decreased significantly in the p-T-miR-7-transfected group (p < 0.05). Moreover, the colon-formation ability of cells was also impaired unmistakably (Figure 1E; p < 0.05), which was consistent with our previous works.^17^, ^18^ To confirm this finding, we also detected the expression of cell-growth-related factors such as CDK2, CDK3, CDK4, and CDK6 in lung cancer cells. As shown in Figure 1F, the relative expression of these CDK family members also decreased significantly in the p-T-miR-7-transfected group compared with those in the control group (p < 0.05). Combining these data demonstrated that miR-7 could be effectively targeted expression by the TTF-1 promoter and suppressed the growth of human lung cells in vitro.

### TTF-1-Promoter-Operating miR-7 Expression Inhibited Tumorigenicity In Vivo

**Figure 2:**
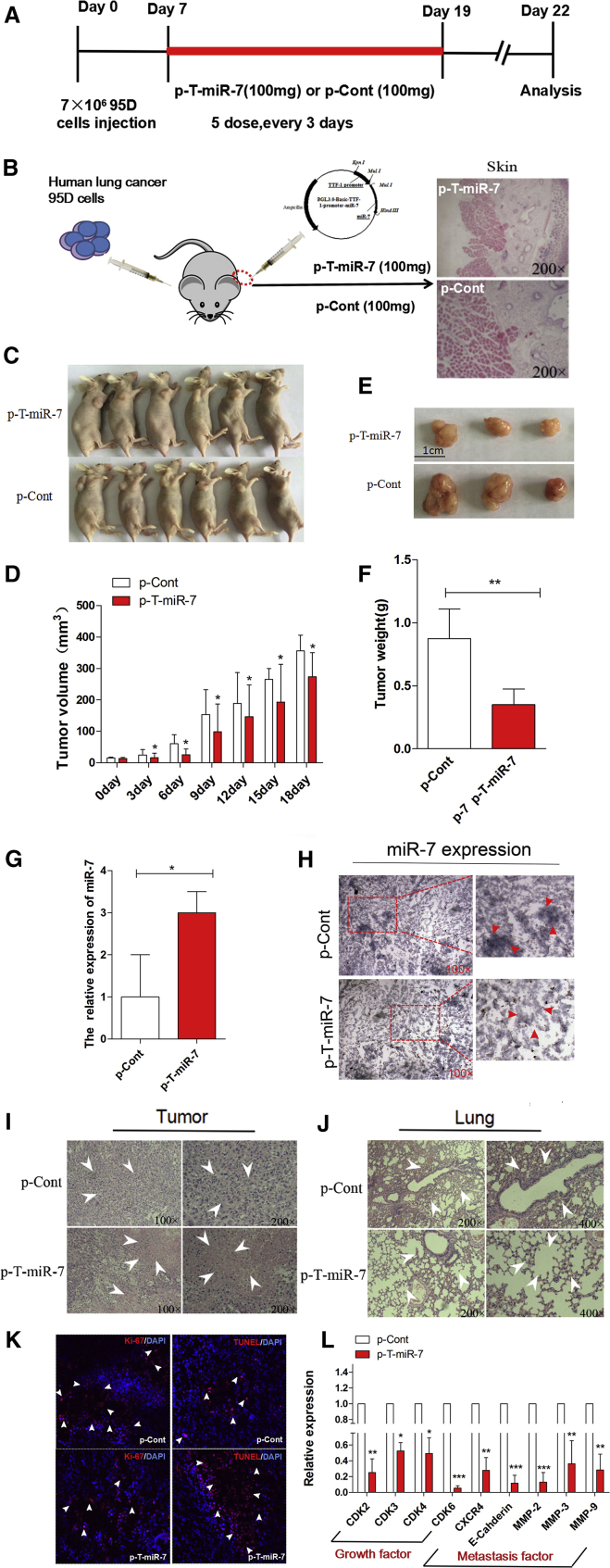
TTF-1-Promoter-Operating miR-7 Expression Suppressed Tumorigenesis of Lung Cancer In Vivo (A) The schedule of study design. (B) Human lung cancer cell line 95D cells were injected subcutaneously into right flank of BALB/c nude mice (n = 8). 7 days later, the plasmid of p-T-miR-7 (100 mg) or p-Cont (100 mg) was remotely given by subcutaneous injection into the left flank of nude mice five times every 3 days. 3 days after last injection, all mice were killed. The skin of injection sections was stained with H&E and observed by microscope (magnification 200×). And partial mice were imaged (C). (D) The growth curve of tumor. (E) The representative morphology and (F) weight of tumor. The expression of miR-7 in tumor tissue was detected by real-time PCR assay (G) and in situ hybridization assay (H). H&E staining of tumor tissue (I) and lung tissue (J). (K) The ability of proliferation (Ki-67) and apoptosis (TUNEL) in tumor tissue were analyzed by immunofluorescence assay. (L) The expression of cell-growth-related molecules (CDK2, CDK3, CDK4, and CDK6) and metastasis-related molecules (CXCR4, E-Cadherin, MMP2, MMP3, and MMP9) in tumor tissue was detected by real-time PCR assay. *p < 0.05, **p < 0.01. ***p < 0.001.

To further explore the potential therapeutic effect of TTF-1-promoter-operating miR-7 expression on tumorigenicity in vivo, a xenograft model of human lung cancer in nude mice was adopted. Human lung cancer cell line 95D cells, which is a highly tumorigenic and metastatic cell line, were injected subcutaneously into right flank of nude mice. 7 days later, the plasmid of p-T-miR-7 or p-Cont was remotely given by subcutaneous injection into the left flank of nude mice five times every 3 days (Figures 2A and 2B). Then, the growth of tumor was observed accordingly. As shown in Figures 2C–2F, both the volume and weight of tumor tissue in the p-T-miR-7 injection group were reduced significantly compared with those in the p-Cont injection group (p < 0.05). Importantly, we analyzed the expression level of miR-7 in tumor tissue and found that the expression level of miR-7 in the p-T-miR-7 injection group was significantly higher than that in the p-Cont injection group (Figure 2G; p < 0.05). To confirm these data, we further detected the expression of miR-7 in tumor mass by in situ hybridization and obtained a similar result (Figure 2H).

Next, we further monitored the impact of TTF-1-promoter-operating miR-7 expression on lung cancer cells in vivo. As shown in Figure 2H, H&E staining showed that there were large areas of necrosis in tumor tissue in the p-T-miR-7 injection group. Moreover, the metastatic index of lung also decreased significantly (Figure 2J; p < 0.05), indicating TTF-1-promoter-operating miR-7 expression also could significantly inhibit the metastasis of lung cancer cells in vivo, which was consistent with our previous report.^17^ Immunofluorescence assay further showed that the proliferation of tumor cells decreased significantly in the p-T-miR-7 injection group (Figure 2L; p < 0.05). On the contrary, the apoptosis of cells increased unmistakably (Figure 2L; p < 0.05). Finally, to confirm the effect of TTF-1-promoter-operating miR-7 expression on the growth and metastasis of tumor cells in vivo, we further detected the expression of cell-growth-related molecules including CDK2, CDK3, CDK4, and CDK6, as well as metastasis-related molecules including CXCR4, E-Cadherin, MMP2, MMP3, and MMP9, in tumor mass, respectively. Data showed that the expression of all of these molecules in the p-T-miR-7 injection group decreased unmistakably (Figure 2l; p < 0.05). All of the above data demonstrated that TTF-1 promoter could effectively operate miR-7 expression in tumor mass, which subsequently inhibited tumorigenicity of lung cancer in vivo.

### TTF-1-Promoter-Operating miR-7 Expression Altered the Transduction of the Akt/Erk Pathway

**Figure 3:**
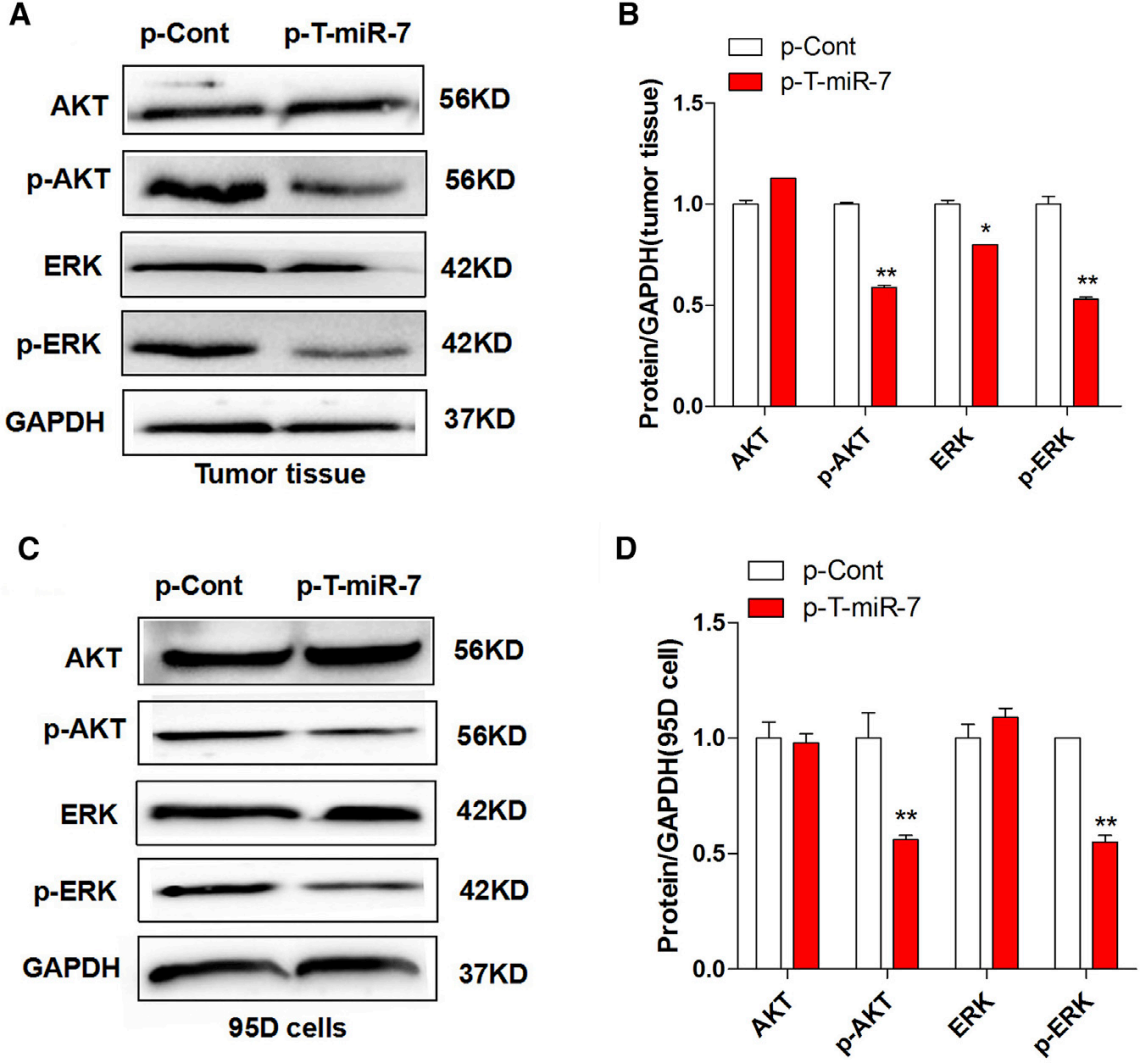
TTF-1-Promoter-Operating miR-7 Expression Altered the Transduction of the Akt and Erk Signaling Pathway Human lung cancer cell line 95D cells were injected subcutaneously into right flank of BALB/c nude mice (n = 8). 7 days later, the plasmid of p-T-miR-7 (100 mg) or p-Cont (100 mg) was remotely given by subcutaneous injection into the left flank of nude mice five times every 3 days. 3 days after last injection, tumor mass was collected. The protein level of Akt, p-Akt, Erk, and p-Erk was detected by western blotting (A) and calculated (B). Human lung cancer cell line 95D cells were transiently transfected with p-T-miR-7 (2 μg) or p-Cont (2 μg) in vitro. 48 hr later, cells were collected, and the protein level of Akt, p-Akt, Erk, and p-Erk was detected by western blotting (C) and calculated (D). *p < 0.05, **p < 0.01. Representative data of three independent experiments are shown.

An increasing body of literatures documented that miR-7 could regulate the growth of lung cancer cells though various signal pathways such as the Akt and Erk pathway.^27^, ^28^, ^29^ Our previous work also showed that miR-7 could inhibit the proliferation and metastasis of lung cancer cells though the Akt pathway.^17^ Thus, to further verify the effect of TTF-1-promoter-operating miR-7 expression on the growth of lung cancer cells, we analyzed the expression of phosphorylation of Akt and Erk in tumor tissue derived from the p-T-miR-7 injection group or the p-Cont injection group, respectively. Data showed that there were not any changes on the expression level of both Akt and Erk in between the p-T-miR-7 injection group and the p-Cont injection group. However, the expression level of phosphor-Akt and phosphor-Erk were decreased significantly in the p-T-miR-7 injection group (Figures 3A and 3B; p < 0.05). To confirm these findings, we also transiently transfected p-T-miR-7 or p-Cont into lung cancer cell line 95D cells in vitro, respectively, and found the expression level of both phosphor-Akt and phosphor-Erk were decreased significantly in the p-T-miR-7-transfected group compared with those in the p-Cont-transfected group (Figures 3C and 3D; p < 0.05). These results suggested that TTF-1-promoter-operating miR-7 expression attenuated the growth of human lung cells in vitro and in vivo by altering the transduction of the Akt/Erk pathway, which was consistent with our previous findings.^17^, ^30^

### TTF-1-Promoter-Operating miR-7 Expression Reduced the Expression of NDUFA4

**Figure 4:**
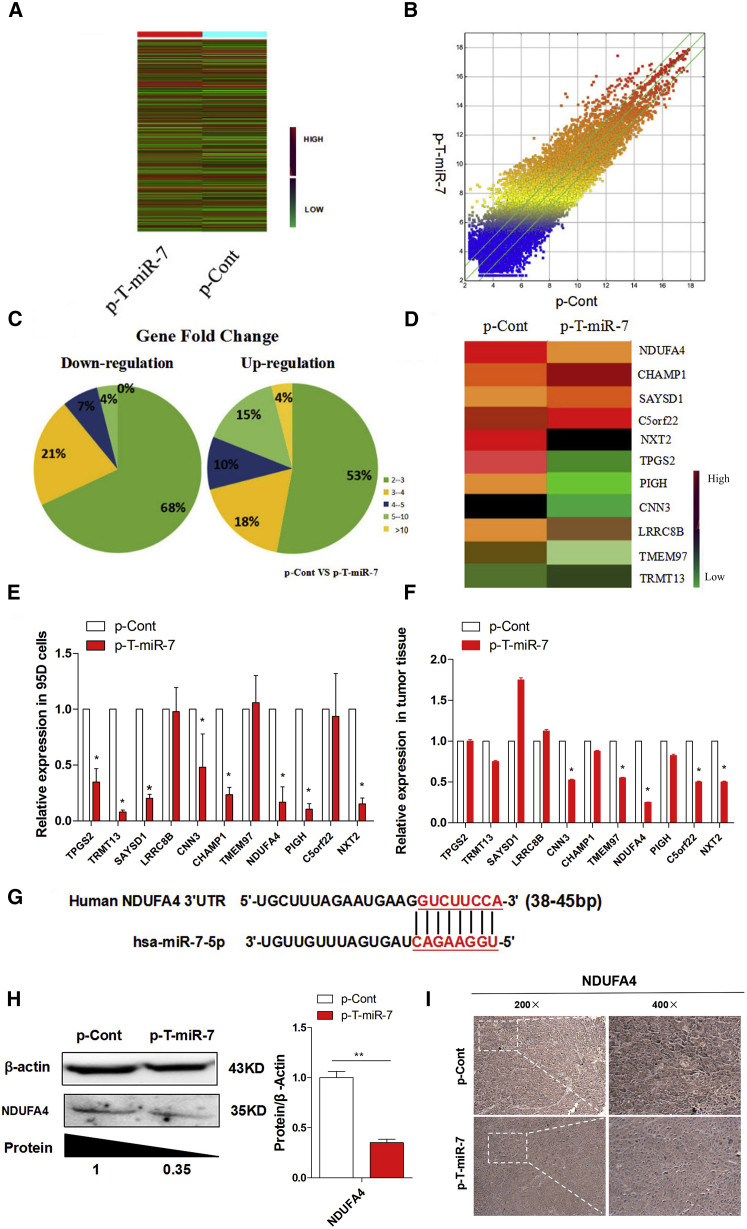
TTF-1-Promoter-Operating miR-7 Expression Reduced the Expression of NDUFA4 Human lung cancer cell line 95D cells were injected subcutaneously into right flank of BALB/c nude mice (n = 8). 7 days later, the plasmid of p-T-miR-7 (100 mg) or p-Cont (100 mg) was remotely given by subcutaneous injection into the left flank of nude mice five times every 3 days. 3 days after last injection, tumor mass were collected. The global gene expression was analyzed by cDNA chip array. (A) Heatmap and (B) scatterplot of gene expression. (C) The fold change and frequency. (D) Prediction of 11 target genes, including NXT2, C5orf22, PIGH, NDUFA4, TMEM97, CHAMP1, CNN3, LRRC8B, SAYSD1 and TRMT13, and TPGS2, by using miRBase and Targetscan software. (E) The expression of these indicated genes were determined by real-time PCR assay. (F) Human lung cancer cell line 95D cells were transiently transfected with p-T-miR-7 (2 μg) or p-Cont (2 μg) in vitro. 48 hr later, cells were collected, and the expression of the indicated genes were determined by real-time PCR. (G) Putative miR-7-binding sites in the 3′ UTR of human NDUFA4. (H) The protein level of NDUFA4 in tumor tissue was analyzed by western blotting and (I) immunohistochemical staining, respectively (original magnification 100×). *p < 0.05, **p < 0.01.

In order to explore the underlying mechanism of TTF-1-promoter-operating miR-7 expression on the growth of lung cancer cells, we analyzed the global gene expression profile in tumor tissue between the p-T-miR-7 and the p-Cont injection group using gene expression microarray assay. The altered gene expression profiles in p-T-miR-7 were shown in a heatmap (Figures 4A and 4B). Given a 4-fold change in differential expression as a cutoff, 568 genes were upregulated, and 534 of them were downregulated (Figure 4C; Table S1). To further elucidate the potential molecular mechanism through which TTF-1-promoter-operating miR-7 expression affected the growth of lung cancer cells, we used miRBase and TargetScan software to compare the downregulated genes in the p-T-miR-7 injection group and found 11 putative miR-7 target genes, including NXT2, C5orf22, PIGH, NDUFA4, TMEM97, CHAMP1, CNN3, LRRC8B, SAYSD1, TRMT13, and TPGS2 (Figure 4D), which also were closely related to tumor cell growth according to previous literatures.^31^, ^32^, ^33^, ^34^ Then, we verified the expression of these 11 predicted target genes, respectively. Unexpectedly, real-time PCR assay showed that only NDUFA4, one target among all predicted target genes of miR-7, was significantly downregulated more than five times both in tumor tissue in the p-T-miR-7 injection group (Figure 4E; p < 0.05) and in p-T-miR-7-transfected tumor cells, respectively (Figure 4F; p < 0.05). Further analysis showed that miR-7 could bind to the 3′ UTR region of NDUFA4 mRNA (Figure 4G). Importantly, western blotting assay further showed that the level of NDUFA4 protein was significantly decreased in tumor tissue in the p-T-miR-7 injection group compared with that in the p-Cont injection group (Figure 4H; p < 0.05). Moreover, we also performed immunohistochemistry assay to detect the expression of the NDUFA4 protein in tumor tissue and obtained a similar result (Figure 4I). In addition, luciferase assay also showed that miR-7 could bind to the 3′ UTR region of NDUFA4 mRNA (data not shown). Collectively, our data indicated that TTF-1-promoter-operating miR-7 expression could affect the growth of tumor cells, which might be closely due to the downregulation expression of NDUFA4.

### Overexpression of NDUFA4 Promoted the Proliferation and Metastasis of Human Lung Cancer Cells

**Figure 5:**
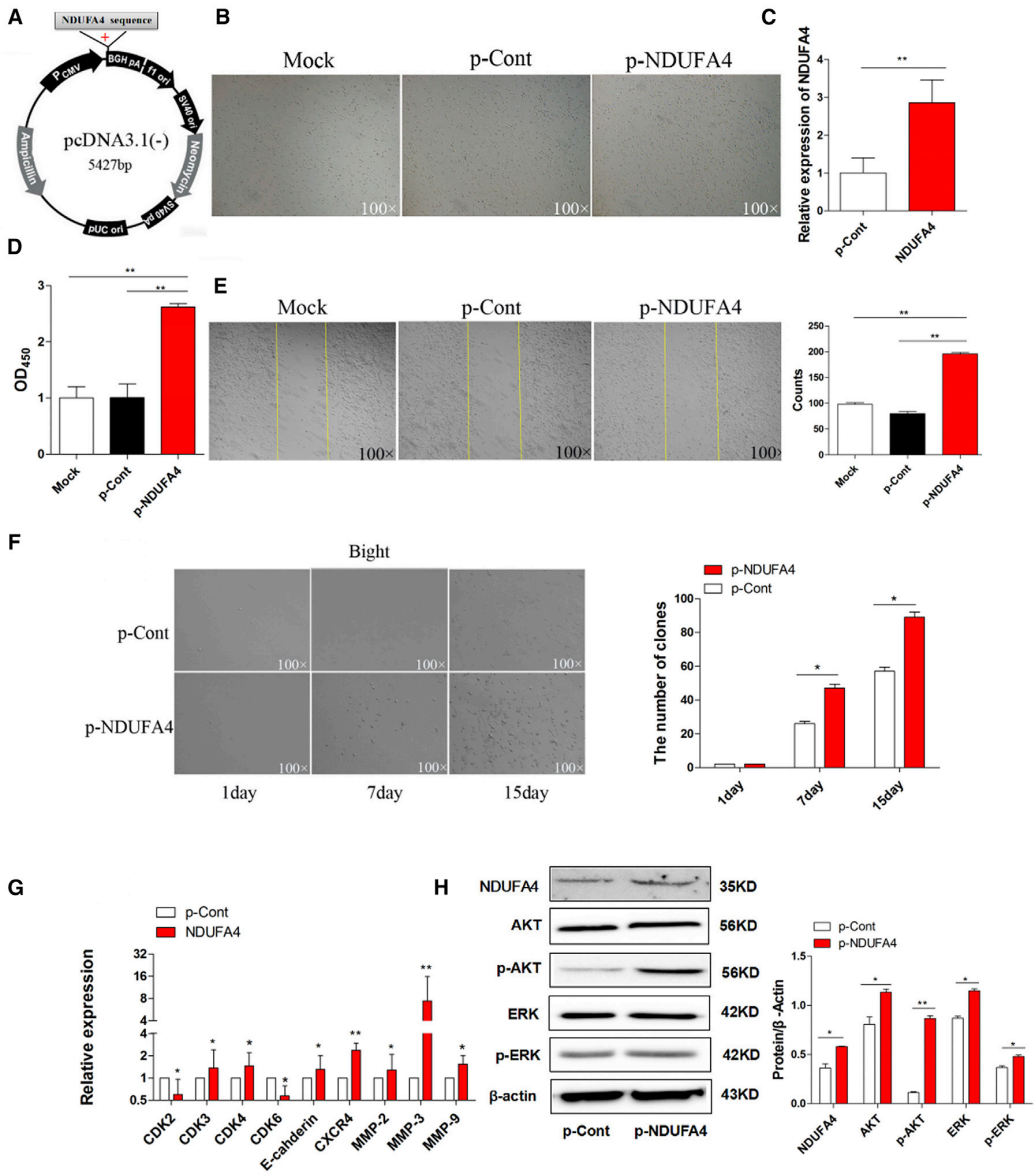
Overexpression of NDUFA4 Promoted the Proliferation and Migration of Human Lung Cancer Cells (A) The sketch map of an eukaryotic expression vector encoding NDUFA4 (termed as p-NDUFA4). (B) Human lung cancer cell line 95D cells were transiently transfected with p-NDUFA4 (10 μg) or p-Cont (10 μg) in vitro. 48 hr later, the morphology of cells were observed by microscopy (magnification 100×). (C) The relative expression of NDUFA4 was determined by real-time PCR. (D) The proliferation of cells was detected by CCK-8 assay. (E) The migration of 95D cells was performed by scratch assay, and the cell number was also calculated. The yellow line indicates the border of scratch wound (magnification 100×). (F) The colon-formation assay also was performed and calculated at indicated time points. Human lung cancer cell line 95D cells were transiently transfected with p-NDUFA4 (10 μg) or p-Cont (10 μg) in vitro. 48 hr later, the expression of indicated cell-growth-related molecules and metastasis-related molecules was detected by real-time PCR assay (G). (H) The protein level of NDUFA4, Akt, p-Akt, Erk, and p-Erk was also detected by western blotting and calculated, respectively. Representative data of three independent experiments are shown.*p < 0.05, **p < 0.01.

Previous works showed that NDUFA4, a subunit of complex IV of the mammalian electron transport chain, played an important role in the development of cancers, such as renal cell carcinoma.^35^, ^36^ However, the knowledge on the potential role of NDUFA4 in the development of lung cancer is still limited. In order to explore whether NDUFA4 played a potent biological role in the growth of lung cancer cells, we constructed an eukaryotic expression vector of NDUFA4 (termed as p-NDUFA4) (Figure 5A) and then transiently transfected p-NDUFA4 into human lung cancer cell line 95D cells in vitro (Figure 5B). Expectedly, real-time PCR assay showed that the expression level of NDUFA4 significantly increased in the p-NDUFA4 transfection group compared with the control group (Figure 5C). Importantly, we found that both the proliferation and migration ability of 95D cells were elevated (Figures 5D and 5E; p < 0.05). Consistent with these findings, the clone-forming ability of cells was also promoted (Figure 5F; p < 0.05).

To further verify the role of NDUFA4 in the growth and metastasis of lung cancer cells, we detected the expression of cell-growth-related molecules including CDK2, CDK3, CDK4, and CDK6, as well as metastasis-related molecules including CXCR4, E-Cadherin, MMP2, MMP3, and MMP9, respectively. Data showed that, compared with the control group, the expression of all of these molecules increased unmistakably in the p-NDUFA4 transfection group (Figure 5G; p < 0.05). Finally, we also analyzed the possible change on the transduction of the Akt and Erk pathway. Western blotting assay showed that the expression level of NDUFA4 protein significantly increased in the p-NDUFA4 transfection group (Figure 5H; p < 0.05). Importantly, we noticed that the expression of phosphor-Akt and phosphor-Erk also increased unmistakably (Figure 5H; p < 0.05). Combining these data indicated that NDUFA4, as an oncogene, could promote growth and metastasis of lung cancer cells by regulating the Akt/Erk signaling pathway.

### Overexpression of NDUFA4 Abrogated the Suppressive Effect of TTF-1-Promoter-Operating miR-7 Expression

**Figure 6:**
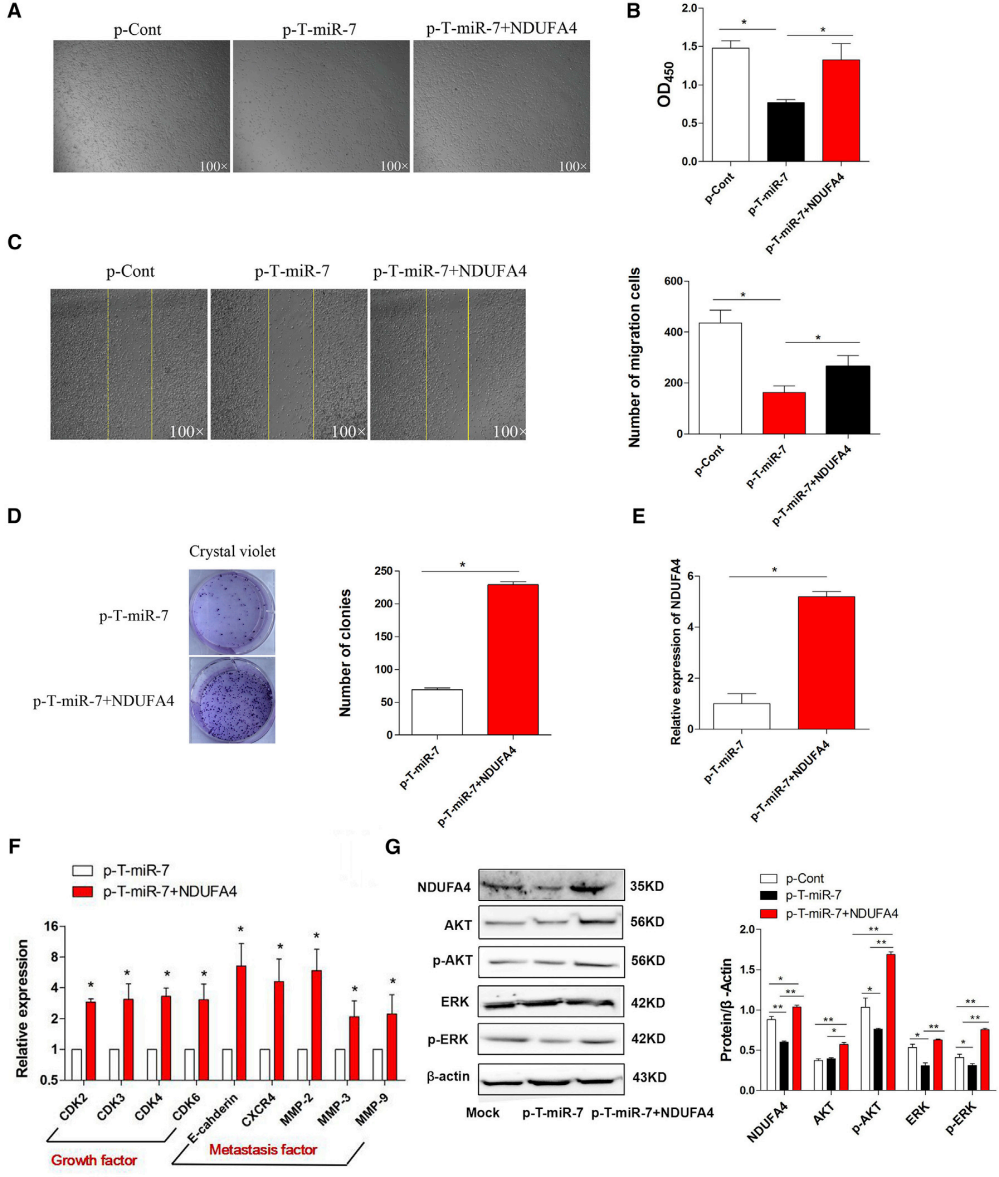
Overexpression of NDUFA4 Abrogated the Suppressive Effect of TTF-1-Promoter-Operating miR-7 Expression Human lung cancer cell line 95D cells were transiently co-transfected with p-T-miR-7 (10 μg) and p-NDUFA4 (10 μg) in vitro. 48 hr later, (A) the morphology of cells were observed by microscopy (magnification 100×). (B) The proliferation of 95D cells was detected by CCK-8 assay. (C) The migration of 95D cells was performed by scratch assay, and the cell number was also calculated. The yellow line indicates the border of scratch wound (magnification 100×). (D) The colony-formation assay was also performed, and the colon numbers were calculated. (E) The relative expression of NDUFA4 was determined by real-time PCR. (F) The relative expression of indicated cell-growth-related molecules and metastasis-related molecules was also detected by real-time PCR assay. (G) The protein level of NDUFA4, Akt, p-Akt, Erk, and p-Erk was detected by western blotting and calculated, respectively. Representative data of three independent experiments are shown.*p < 0.05, **p < 0.01.

Then, to further explore whether downregulation of NDUFA4 contributed to the suppressive effect of TTF-1-promoter-operating miR-7 expression on human lung cancer cells, we transiently co-transfected p-T-miR-7 and p-NDUFA4 into lung cancer cell line 95D cells and observed the possible change on cell growth and metastasis. As shown in Figures 6A and 6B, the proliferation of 95D cells decreased significantly in the p-T-miR-7 transfection group, which was consistent with our above data. Notably, we found that the proliferation of cells in the p-T-miR-7 and p-NDUFA4 co-transfection group elevated unmistakably (p < 0.05). Moreover, clone-formation assay showed that clone-formation ability of cells also increased significantly (Figure 6D; p < 0.05). Further analysis showed that the migration ability of cells in the p-T-miR-7 and p-NDUFA4 co-transfection group was enhanced compared with that in the p-T-miR-7 transfection group (Figure 6C; p < 0.05). Consistently, real-time PCR assay showed that the expression of NDUFA4, cell-growth-related molecules including CDK2, CDK3, CDK4, and CDK6, as well as metastasis-related molecules including CXCR4, E-Cadherin, MMP2, MMP3, and MMP9 were elevated significantly (Figures 6E and 6F; p < 0.05). Finally, we also analyzed the expression of phosphor-Akt and phosphor-Erk in the p-T-miR-7 and p-NDUFA4 co-transfection groups. Data showed that the expression of NDUFA4 protein increased unmistakably (Figure 6G; p < 0.05). Importantly, the level of both phosphor-Akt and phosphor-Erk significantly increased (Figure 6G; p < 0.05). Combining these results demonstrated that TTF-1-promoter-operating miR-7 expression affected the growth and metastasis of human lung cancer cells through NDUFA4.

### The Change on Organs and Tissues in Nude Mice Model of Human Lung Cancer

**Figure 7:**
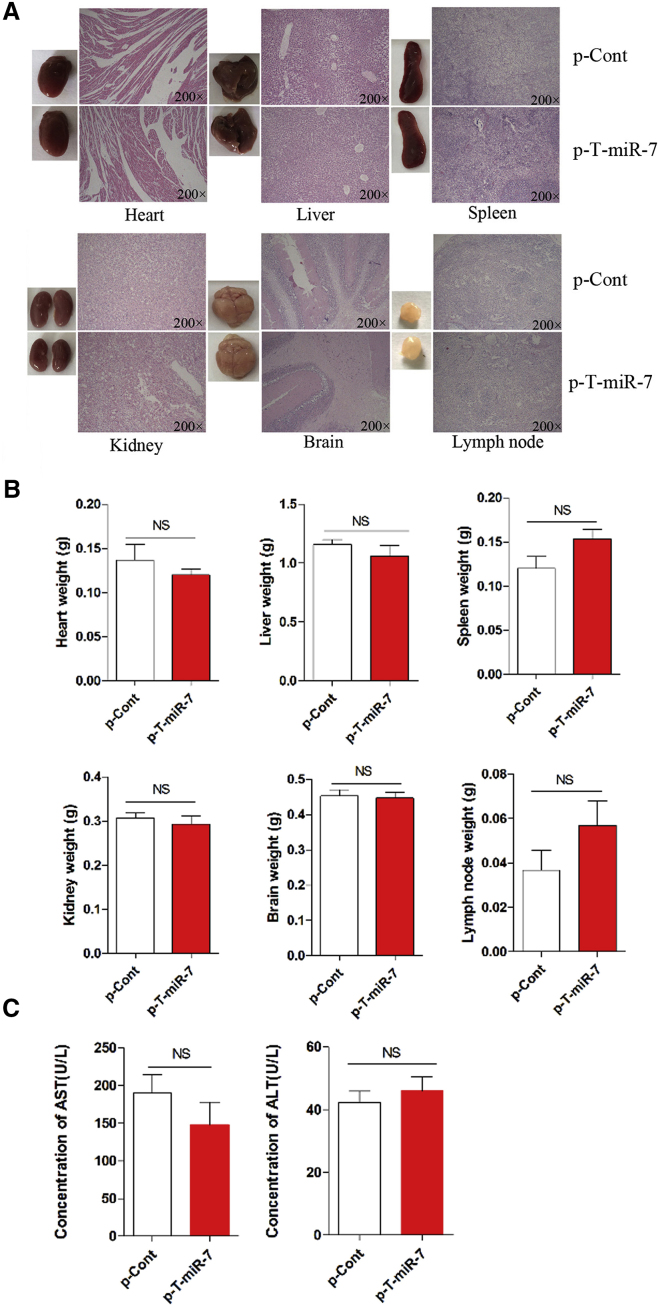
The Change on Important Organs and Tissues in Nude Mice Model of Human Lung Cancer Human lung cancer cell line 95D cells were injected subcutaneously into right flank of BALB/c nude mice (n = 8). 7 days later, the plasmid of p-T-miR-7 (100 mg) or p-Cont (100 mg) was remotely given by subcutaneous injection into the left flank of nude mice five times every three days. 3 days after last injection, all of the mice were sacrificed. (A) The morphology and H&E staining of various organs and tissues, including heart, liver, spleen, kidney, brain and lymph nodes, were performed. (B) The weight of indicated organs and tissues also were obtained. (C) The concentration of the serum AST and ALT were measured, respectively. NS, no significance.

Previous data showed that the biological change on organs and tissues were important for the relative safety of targeted gene therapy against cancer, which was critical for its potential application.^37^, ^38^ Then, we observed the possible change on various important organs and tissues in nude mice model of human lung cancer. As shown in Figure 7A, there were no significant changes in the morphology of six important organs and tissues, including heart, liver, spleen, kidney, brain, and lymph nodes, between the p-T-miR-7 injection group and the control group (p > 0.05). Moreover, we also did not find any difference in histology or weight in these organs and tissues (Figure 7B; p > 0.05). To confirm these findings, we further collected and detected the concentration of serum AST and ALT, which were critical indicators for the functional change of organs including heart and liver. It was noticed that none of these biochemistry indicators changed significantly (Figure 7C; p > 0.05), indicating that there were not any changes on biological function of important organs and tissues in the p-T-miR-7 injection group.

Finally, we preliminarily estimated the distribution of plasmid p-T-miR-7 in vivo. As shown in Figure S2, the copies of the p-T-miR-7 plasmid was higher in lung tissue and tumor tissue (p < 0.05), but not in the other organs and tissues, indicating the plasmid was mainly distributed in the lung tissue and tumor tissue in vivo. However, when we further analyzed the expression level of miR-7, it was found that, compared with their corresponding control, miR-7 level increased significantly in heart, spleen, lung, intestine, and lymph nodes, respectively (Figure S3), indicating that intrinsic transcriptional factors in these organs and tissues might bind to TTF-1 promoter and manipulate the expression of miR-7.

## Discussion

It is the first time the potential value of the TTF-1 promoter operating distinct miRNAs expression in targeted gene therapy against lung cancer has been explored. We first observed that TTF-1 promoter could effectively operate miR-7 expression in lung cancer cells. Importantly, we found that TTF-1-promoter-operating miR-7 expression could effectively inhibit the growth of lung cancer cells in vitro and in vivo. Notably, we further revealed that the downregulation of NDUFA4, a novel target of miR-7, contributed to the effects of miR-7 expression operated by TTF-1 promoter on the growth and metastasis of human lung cancer cells, accompanied by altered transduction of related signaling pathway including the Akt and Erk pathway. Finally, we observed that there were not any significantly change in various important organs or tissues, indicating the safety value of strategy of TTF-1-promoter-operating expression of miR-7 in vivo.

It is well known that miR-7, as an intrinsic tumor suppressor, has been found to be an important regulator in the development of various cancers including lung cancer.^13^, ^39^, ^40^, ^41^ For example, Xiong et al.^42^ documented that miR-7 could inhibit the growth of human lung cancer cells in vivo. And our recent work reported that the site mutation of miR-7 promoter region contributed to its altered expression in clinical lung cancer tissue.^18^ Interestingly, some research works showed that in situ local injection of miR-7 overexpression plasmids could regulate the growth and metastatic potential of human lung cancer cells in vivo via the Akt pathway.^43^, ^44^ In the present study, we further extended previous findings to report that expression of miR-7, operated by TTF-1 promoter, could reduce the growth and metastasis of human lung cancer cells in vitro. Importantly, remote hypodermic injection, but not local injection, of plasmid p-T-miR-7 could significantly inhibit the growth and metastasis of human lung cancer cells in vivo, accompanied by altered expression of growth- and metastasis-associated molecules such as CDK and MMP family members. Therefore, combining these research works may highlight the fact that miR-7 is a critical regulator and might be an ideal target for gene therapy against clinical lung cancer, which would be helpful for the outcome of clinical treatment.

The technique of targeted gene expression, including distinct delivery systems and specific gene-promoter-operating expression, is an important strategy for gene therapy against various cancers including lung cancer.^4^, ^7^, ^45^, ^46^ Up to now, extensive literatures documented the efficacy of distinct delivery systems, such as the liposome system, nano-partical system, and polyetherimide (PEI) system, and so on, in cancer gene therapy.^6^, ^47^, ^48^ However, related documents on the potential value of targeted expression of interesting genes using specific gene promoter, such as tumor suppressor, in cancer gene therapy is still scare. The main obstacle might be the selection of ideal promoter in cancers. The TTF-1 gene is limitedly expressed in lung tissue and thyroid tissue. Interestingly, TTF-1 gene was a well-known lineage-specific oncogene in lung cancer, which dominantly expressed in lung cancer but not in other types of cancers.^20^, ^21^, ^22^ Therefore, in the present study, we attempted to design and construct an eukaryotic vector encoding miR-7, which was manipulated by the TTF-1 promoter. Luckily, we found that this plasmid could effectively express miR-7 in human lung cancer cells with a higher expression level of TTF-1 in vitro, but not other human cancer cells with a lower expression level of TTF-1, including colon cancer, hepatocellular carcinoma, and gastric cancer, indicating that the TTF-1 promoter could effectively orchestrate targeted expression of miR-7 in lung cancer cells. Importantly, we further found that TTF-1-promoter-operating miR-7 expression could significantly not only inhibit the growth and metastasis of human lung cancer cells in vivo, but also induce the apoptosis of cancer cells in vivo. These data suggested that TTF-1 promoter might be an ideal candidate operator for targeted expression of interesting genes in lung cancer gene therapy. Therefore, successive research work on both the potential effect of TTF-1-promoter-operating miR-7 expression on other types of cancers and regulatory factors including transcript factors in the activation of TTF-1 promoter, which did not been investigated in present study, is valuable for the verification of usage of the TTF-1 promoter in targeted gene expression in lung cancer and can ultimately benefit the development of a therapeutic strategy in clinical lung cancer.

Accumulating evidence showed that the molecular mechanism through which miR-7 regulated the growth and metastasis of lung cancer cells was complex, and various genes including PA28 gamma, KLF4, BCL-2, and so on also were reportedly involved in the biological function of miR-7 in lung cancer.^15^, ^49^, ^50^ To reach a comprehensive knowledge on the molecular mechanism of the effect of TTF-1-operating miR-7 expression on the growth and metastasis of lung cancer cells, we used a global gene expression chip technique combined with biological information analysis to screen the potential target of miR-7. Unexpectedly, we found that TTF-1-operating miR-7 expression could significantly reduce the expression level of NDUFA4 in lung cancer cells in vitro and in vivo. Importantly, overexpression of NDUFA4 could abrogate the effect of TTF-1-operating miR-7 expression on the growth and metastasis of lung cancer cells, accompanied by altered transduction of the Akt and Erk pathway, which was critical for the growth and metastatic potential of lung cancer cells. These data demonstrated that NUDFA4, a novel target of miR-7, contributed to the effects of TTF-1-operating miR-7 expression in lung cancer cells. In particular, we also found that overexpression of NUDFA4 could enhance the proliferation and metastatic potential of human lung cancer cells and elevate the transduction of the Akt and Erk pathway. In accordance with these findings, recent literatures reported that the expression level of NDUFA4 was closely associated with the progression and/or worse prognosis of various types of cancers.^6^, ^35^ For example, Franziska et al.^36^ found that downregulated expression of NDUFA4 in clear cell renal cell carcinoma was related to cancer-specific survival. These research works suggested that NDUFA4 may be as an oncogene in the development of cancers including lung cancer. However, the exact role of NDUFA4 in the development of lung cancer, such as the value of NDUFA4 expression in prognosis of lung cancer patients, still remains to be fully elucidated in successive research work.

It is well known that the safety of gene therapy strategy, including functional change of important organs and distribution of exogenous DNA, was critical for potential application of cancer gene therapy.^37^, ^38^, ^51^ In the present study, we monitored the possible change on several important organs including liver, heart, spleen, and so on and found that there were not any difference on morphology or histology of these important organs and tissues. Moreover, we also found that there were not significant changes in serum level of aspartate transaminase (AST) and alanine aminotransferase (ALT), which were representative reflectors in the functional change of organs including heart and liver. Regarding distribution of plasmid, Thanaketpaisarn et al.^52^ reported that naked plasmid DNA (pDNA) encoding firefly luciferase was directly injected into the tail vein of mice and found that the plasmid was mostly enriched in liver, spleen, and kidney. Mahato et al.^53^ further investigated the disposition characteristics of pDNA complexed with cationic liposomes after intravenous injection in mice and found that liposomal pDNA encoding gene expression was enriched in lung, heart, kidney, and spleen, but not in liver. Some literature documented naked pDNA enriched in the liver in vivo after hydrodynamic injection via tail vein.^54^ Different from these research works, we analyzed the distribution of plasmid p-T-miR-7 after remote hypodermic injection and found that the plasmid was dominantly enriched in lung tissue and tumor mass in vivo. To the diverse phenomenon, we proposed two factors may be closely related to the different distribution of pDNA in vivo. The first factor was the different experimental settings including entry route and way, as well as the dosage, of naked pDNA. The second factor was the different hemodynamics and perfusion in distinct organs and tissues. Thereby, further studies on the dynamic distribution of the plasmid and the possible change on other organs are important for the evaluation of safety of the plasmid in vivo, which is critical for the potential application of gene therapy based on targeted miR-7 expression in lung cancer.

In conclusion, for the first time, our study demonstrated that TTF-1-promoter-operating miR-7 expression could significantly inhibit the growth of human lung cancer in vitro and in vivo, closely related to downregulation of NDUFA4 and altered transduction of related signaling pathways including Akt and Erk. These data indicated that TTF-1-promoter-operating miR-7 expression might be an ideal strategy in lung cancer, which provided preliminary experimental basis for targeted expression of distinct miRNA in lung cancer and was helpful for the development of gene therapy against clinical lung cancer.

## Materials and Methods

### Construction of Eukaryotic Vector

The genes for the miR-7 (NM-407044) were expanded by PCR from human DNA derived from 95D cells using a forward primer (5′-CGACGCGTAAGAGAGAAATGAGCCACTTGC) and a reverse primer (5′-CCCAAGCTTCCTGCCACAGTGGGGGATG) and then subcloned into *MulI* and HindIII sites of pGL3.0 basic vector (Invitrogen) to generate pGL3.0-basic-miR-7 vector (termed as p-miR-7). Afterward, for the construction of the PGL3-basic-TTF-1-promoter-miR-7 (termed as p-T-miR-7) vector, the promoter region of TTF-1 (NM-7080) was amplified from DNA derived from 95D cells using a forward primer (5′-CGGGGTACCTGTTTCGGCAACTAC) and a reverse primer (5-CGACGCGTCCTTCTGGGTCCTT) and subcloned into *KpnI* and *MulI* sites of p-miR-7 vector. The gene for the NDUFA4(NM-4697) were expanded by PCR from human cDNA derived from 95D cells using a forward primer (5′-GCTCTAGAGGCTAGGTCGGTTCTCTCCT) and a reverse primer (5′-CGGGATCCGTGGAAAATTGTGCGGATGT) and then subcloned into *XbaI* and *BamHI* sites of pcDNA3.1 vector (Invitrogen) to generate pcDNA3.1-NDUFA4 vector (termed as p-NDUFA4). Clone identity was verified using restriction digest analysis and plasmid DNA sequencing. Endotoxin-free plasmids were obtained using Endofree plasmid mega kit (QIAGEN). Then, plasmids were transiently transferred into the 95D cells using Lipofectamine-2000 (Invitrogen) in different following experiments according to the manufacturer’s instructions.

### Cell Culture and Transfection

Human lung cancer cell line 95D cells, A549 cells, NCI-H292 cells, gastric cancer cell line SGC901 cells, hepatic cancer cell line HepG2 cells, as well as colon cancer cell line SW620 cells were obtained from National Rodent Laboratory Animal Resource. Colon cancer cell line SW620 cells were cultured in McCoy 5A, RPMI-1640 containing 100 IU/mL penicillin, 100 μg/mL streptomycin, 20 mM glutamine, and 10% heat-inactivated fetal bovine serum (FBS). All of other cells were cultured in RPMI-1640 containing 100 IU/mL penicillin, 100 μg/mL streptomycin, 20 mM glutamine and 10% heat-inactivated fetal bovine serum (FBS). All cells were cultured in a humidified atmosphere of 5% CO_2_ at 37°C. For transfection, cells were seeded at 70%–80% confluence, and 12 hr later cells were transiently transfected with indicated vectors with Lipofectamine 2000 according to the manufacturer’s instruction. Cells were harvested at indicated time point in following experiments.

### Real-Time PCR Assay

The conventional primers were obtained from Shanghai Sangon Biological Engineering, the TaqMan probes of miR-7 (000386) and U6 (001793) were purchased from Life Technologies, and the other reagents were from TAKARA Bio. RT-PCR and real-time PCR were performed according to the manufacturer’s protocols. The following primers were used: CDK6: forward: 5′-AAGCCTCTTTTTCGTGGAAGT-3′, reverse: 5′-GGTTGGGCAGATTTTGA-ATG-3′; CDK4, forward: 5′-ATTGGTGTCGGTGCCTATG-3′, reverse: 5′-AACTGTGCTGATGG-GAAGG-3′; CDK3, forward: 5′-GCTCTTTCGTATCTTTCGTATGC-3′, reverse: 5′-ATTGGTGTCGGTGCCTATG-3′; CDK2, forward: 5′-TTTGCTGAGATGGTGACTCG-3′, reverse: 5′-TGGGGA-AACTTGGCTTGTAA-3′; E-cadherin, forward: 5′-TGATTCTGCTGCTCTTGCTG-3′, reverse: 5′-CTCTTCTCCGCCTCCTTCTT-3′; CXCR4, forward: 5′-TGACCGCTTCTACCCCAAT-3′, reverse: 5′-AGCCAGGATGAGGATGACTG-3′; MMP9, forward: 5′-TCTTCCCCTTCACTTTCCTG-3′, reverse: 5′-CCCACTTCTTGTCGCTGTC-3′; MMP3, forward: 5′-ATCCCGAAGTGGAGGAAAAC3′, reverse: 5′-AGCCTGGAGAATGTGAGTGG-3′; MMP2, forward: 5′-TATGGCTTCTGCCCT-GAGAC-3′, reverse: 5′-CACACCACATCTTTCCGTCA-3′; TPS2, forward: 5′-CAAAAGTGGGA-AACCAGCAT-3′, reverse: 5′-GATGAGCAGGCGGTAATAGG-3′; TRMT13, forward: 5′-TGTCCCATCCAGCATTACAC-3′, reverse: 5′-GCTCCAAACTCAACAAAGCA-3′; SAYSD1, forward: 5′GCAGCACATCAGAGACACCA-3′, reverse: 5′-GCAGGACCAACCAGAGAAGA-3′.LRRC8B, forward: 5′-GTGGTGGATGCTGAGGAGTT-3′, reverse: 5′-AGCCAGATGAAGGATGAAGG-3′; CNN3, forward: 5′-AATGAGTGTGTATGGGCTTGG-3′, reverse: 5′-TGTTCCTGTTCCTTGGCTTC-3′; CHAMP1, forward: 5′-ATGAAGCGTGGAAAAGGAAA-3′, reverse: 5′-GCATTTGTAAG-GGCTATGAACA-3′; TMEM97, forward: 5′-TGCCCCCTACTTACTCATCC-3′, reverse: 5′-CAA-CAAGCAACCACCCTGTA-3′; NDUFA4, forward: 5′-TCCCCCTCTTTGTATTTATTGG-3′, reverse: 5′-GGGCTCTGGGTTATTTCTGTC-3′.PIGH, forward: 5′-CCAGAAAGCCACATCAACAA-3′, reverse: 5′-TACGGAAAACCAGCCCCTAT-3′; C5orf22, forward: 5′-GGCACCAACCTA–ACAGAGGA-3′, reverse: 5′-CCGTTTCTTCATCATCACCA-3′; NXT2, forward: 5′-ACTGCTAC-AAGGTCCCAGATG-3′, reverse: 5′-TGGTTAGTGCCCGTCTTCTT-3′. Gene expression levels were quantified using the BIO-RAD CFX96 detection system (Bio-Rad). Relative expression of these indicated genes was calculated using the comparative threshold cycle (Ct) method.

### Cell Counting kit-8 Assay

95D cells were seeded in 96-well plates at 1 × 10^4^/well with triplicate and transiently transfected with p-T-miR-7 plasmid (2 μg), p-NDUFA4 plasmid (10 μg), or p-Cont plasmid (2 μg/10 μg). At indicated time points, cells were detected using cell-counting kit-8 (CCK-8) assay. In brief, 20 μL CCK-8 solution was added into each well. After 3 hr of incubation at 37°C, the absorbance was measured with a spectrophotometer at 450 nm with 600 nm as a reference.

### Colony-Formation Assay

95D cells were transiently transfected with p-T-miR-7 plasmid (2 μg), p-NDUFA4 plasmid (10 μg), or p-Cont plasmid (2 μg/10 μg), as described above. 24 hr later, cells were trypsinized to single-cell suspension and seeded in 6-well plates at 200/well and 800/well for the clone-forming experiment. Then, cells were incubated in a humidified atmosphere of 5% CO_2_ at 37°C. 15 days later, the colonies were stained with crystal violet, and the colony diameter and number were statistically analyzed.

### Animal Experiment

All animals were housed in the pathogen-free mouse colony at our institution, all animal experiments were performed according to the Guidelines for the Care and Use of Laboratory Animals (Ministry of Health, People’s Republic of China, 1998), and all the experimental procedures were approved by the ethical guidelines of Shanghai Medical Laboratory Animal Care and Use Committee (permit number: 2013018). Female nude mice (BALB/C, 4−6 weeks old) were purchased from Shanghai Laboratory Animal Center. For preparation of subcutaneous xenograft model, 0.2 mL lung cancer cell line 95D cells (7.0 × 10^6^) was injected subcutaneously into the right flank of nude mice. 7 days after tumor cell inoculation with confirmation of successful maturation of tumors, 16 mice were divided randomly into two groups (eight mice per group). The plasmid p-T-miR-7 or p-Cont (100 mg) was given locally by direct injection into the left flank of nude mice five times every 3 days. Meanwhile, tumor volumes were determined (in cubic millimeter) as previous description.^55^ After 18 days of treatment, all mice were sacrificed. Tumor tissue and other organs were excised and divided into two parts. One part was stored in −80°C for further use. And another section was fixed in formalin and embedded in paraffin used for H&E staining.

### In Situ Hybridization

To evaluate the cellular distribution of miR-7 in the tumors, in situ hybridization was performed according to our previous description^56^ with some modifications. Briefly, before hybridization incubation, all solutions were prepared with diethyl pyrocarbonate-treated water. After deparaffinization and rehydration, tissue sections were treated by proteinase K digestion. After blocking with normal goat serum (1:100), sections were next incubated or microwave heating and then incubated with hybridization cocktail containing miR-7 probe (1:1,000 dilution; EXIQON; no. 38485-01) at 42°C for 16 hr. Then, alkaline phosphatase-labeled anti-digoxigenin antibody (1:500) (Roche Diagnostics) and the reaction products were colorized with nitro blue tetrazolium/5-bromo-4-choloro-3-indolyl phosphate (NBT/BCIP) (ZSGB-Bio). Then, the tissues were counterstained with Mayer’s hematoxylin and systematically viewed under a light microscope (Olympus IX-71).

### Histopathology

Tumor tissue and other organs were fixed in 4% paraformaldehyde and embedded in paraffin and then cut into 5-μm-thick sections. Sections were stained with H&E, and images were taken with an Olympus IX71 microscope. All tumor tissues and other organs fields at original magnification 10×, 20×, and 40× were examined for each sample.

### Immunofluorescence: Ki-67 Staining and TUNEL Assay

To assess tumor cell proliferation and apoptosis in situ after the plasmid of p-T-miR-7 injection, immunofluorescence staining using anti-proliferation cell nuclear antigen (Ki-67) antibody and TUNEL (terminal deoxynucleotidyl transferase dUTP nick end labeling) assay was performed on all frozen sections of tumor tissues. After being deparaffinized and rehydrated, slides were incubated in citrate buffer (pH 6.0), and then antigen retrieval was performed in a microwave two times for 5 min. After cooling down at room temperature, slides were washed twice for 5 min with PBS and incubated with 10% normal goat serum for 30 min at room temperature. First, to determine tumor cell proliferation, anti-Ki-67 antibody (sc-7907, 1/100, rabbit-anti-mouse, Santa Cruz Biotechnology) in PBS were added, and the slides were incubated at 4°C overnight. After three washes in PBS for 5 min, secondary antibody Alexa-Fluor-488-conjugated goat-anti-rabbit immunoglobulin G (IgG) (1/250, Invitrogen) was added and allowed to incubate for 1 hr in the dark at room temperature. After three washes in PBS for 5 min, the slides were counterstained, mounted with SlowFade Gold antifade reagent with DAPI (Invitrogen), and left for 10 min in the dark at room temperature before examination by fluorescence microscopy (Zeiss Axioplan 2). In addition, for testing tumor cell apoptosis in situ, a TUNEL assay was performed according to the manufacturer’s protocol. In brief, after antigen retrieval, the slides were washed in PBS three times and incubated with 50 μL of TUNEL reaction mixture (11684817910, In situ Cell Death Detection Kit, Roche) for 2 hr at 37°C. At last, after three washes in PBS for 5 min, the slides were also counterstained by DAPI (Invitrogen) and evaluated by fluorescence microscopy.

### Wound-Healing Assays

Human lung cancer 95D cells were transiently transfected with p-T-miR-7 plasmid (2 μg), p-NDUFA4 plasmid (10 μg), or p-Cont plasmid (2 μg/10 μg) or scramble control as described above. Cells were then seeded in 6-well plates at 1 × 10^5^ per well in growth medium. Confluent monolayers were starved overnight in assay medium, and a single scratch wound was created using a micropipette tip. The cells were washed with PBS to remove cell debris. Images were captured with a microscope at 48 hr postwounding. For a quantitative measure, we counted the cells migrating to the wound area based on the image at 0 hr postwounding.

### Western Blotting

Western blotting was performed on cytosolic cellular extracts. Equal amounts of protein were resolved under reducing conditions on a 10% SDS-PAGE gel. Protein migration was assessed using protein standards (Bio-Rad). Transfer to a nitrocellulose membrane was performed 60 min at 250 mA using a wet transfer system. Equal protein loading was confirmed with Ponceau staining. The membrane was washed in 5% skim milk in PBS plus 0.05% Tween 20 (PBST) for 2 hr to block nonspecific protein-binding sites on the membrane. Immunoblotting was performed using rabbit polyclonal anti-human NDUFA4 antibody (Santa Cruz Biotechnology; no. Sc-517091), rabbit monoclonal anti-human ERK antibody (Cell Signaling Technology; no. 4695), rabbit polyclonal anti-human p-ERK antibody (Cell Signaling Technology; no. 4370), rabbit monoclonal anti-human AKT antibody (Cell Signaling Technology; no. 4691), rabbit monoclonal anti-human p-AKT antibody (Cell Signaling Technology; no. 4060), and rabbit-monoclonal anti-human GAPDH antibody (Cell Signaling Technology; no. 2118) or rabbit monoclonal rabbit monoclonal anti-human β-actin antibody, respectively (Cell Signaling Technology; no. 4970) at a dilution of 1/1,000 in a non-fat milk-Tris buffer. The membrane was then washed in PBST and subsequently probed with a secondary anti-rabbit antibody (Ab)-conjugated to horseradish peroxidase (HRP) (Cell Signaling Technology; no. 7074) at a dilution of 1:2,000. The signal was detected and analyzed using the chemiluminescence Imaging System (ChemiScope5600, CLINX); each experiment was performed in triplicate.

### Gene Expression Array

Total RNA was first converted to cDNA, followed by in vitro transcription to make cRNA. 5 μg of single-stranded cDNA was synthesized, end labeled, and hybridized, for 16 hr at 45°C, to Human Gene 1.0 ST arrays. All washing steps were performed by a GeneChip Fluidics Station 450 and GeneChip were scanned with the Axon GenePix 4000B microarray scanner. Partek was used to determine ANOVA p values and fold changes for genes.

### Immunohistochemistry

Immunohistochemical staining was performed following standard procedures. The formalin-fixed paraffin-embedded tissues were sliced into 5-μm-thick sections, deparaffinized, and rehydrated. Then, the cells were fixed with 95% ethanol for 15 min. Antigen retrieval was performed in 10 mmol/L citric acid buffer (pH 6.0) for 10 min using a 750-W microwave. Endogenous peroxidase activity was blocked with 3% hydrogen peroxide in methanol for 15 min. After incubation with rabbit anti-human NDUFA4 antibody (1:1,000 dilution; Santa Cruz Biotechnology; no. Sc-517091), the sections were washed in PBA and incubated with a polymer horseradish peroxidase-conjugated secondary antibody (ZSGB-Bio) for 60 min. The sections were further incubated with Liquid DAB Large-Volume Substrate-Chromogen System (ZSGB-Bio) and counterstained with hematoxylin. The immunostaining was evaluated using an Olympus IX-71 light microscope (Olympus).

### Statistical Analyses

The data were analyzed with GraphPad Prism 5.0 and were presented as the mean ± SD. Student’s t test was used when two conditions were compared, and ANOVA with Bonferroni or Newman-Keuls correction was used for multiple comparisons. p < 0.05 was considered significant; two-sided tests were performed.

## Author Contributions

L.L. and C.C. performed the experiments, analyzed the data, and wrote the paper; J.Z. performed the experiments and analyzed the data; H.W. and M.G. performed the experiments; Y.Z., J.L., and J.Z. wrote the paper; L.X. conceived and designed the experiments, analyzed the data, and wrote the paper, and all authors reviewed the paper.

## Conflicts of Interest

All authors declare that the research was conducted in the absence of any commercial or financial relationships that could be construed as a potential conflict of interest.

## Supplemental Information

Document S1. Supplemental Materials and Methods, Figures S1–S3, and Table S1

Document S2. Article plus Supplemental Information
