# Supplementary material for: Targeted Expression of miR-7 Operated by TTF-1 Promoter Inhibited the Growth of Human Lung Cancer through the NDUFA4 Pathway
Source: Mol Ther Nucleic Acids. 2016 Dec 31;6:183–97. doi: 10.1016/j.omtn.2016.12.005 (PMC5363496; doi:10.1016/j.omtn.2016.12.005)
Supplement: Document S1. Supplemental Materials and Methods, Figures S1–S3, and Table S1 [file mmc1.pdf]

OMTN, Volume *6*

Supplemental Information

Targeted Expression of miR-7 Operated by TTF-1 Promoter Inhibited the Growth of Human Lung Cancer through the NDUFA4 Pathway

Liangyu Lei, Chao Chen, Juanjuan Zhao, HaiRong Wang, Mengmeng Guo, Ya Zhou, Junming Luo, Jidong Zhang, and Lin Xu

Material and methods

Construction of eukaryotic vector

The gene for the miR-7 (NM-407044) were expanded by PCR from human DNA derived from 95D cells using a forward primer (5′-CGACGCGTAAGAGAGAAATGAGCCACTTGC) and a reverse primer (5′-CCCAAGCTTCCTGCCACAGTGGGGGATG) and then subcloned into and *Mul I* and *HindIII* sites of pGL3.0 basic vector (Invitrogen Corp, San Diego, California, USA) to generate pGL3.0- basic-miR-7 vector (termed as p-miR-7). Afterwards, for the construction of the PGL3-basic-TTF-1-promoter-miR-7(termed as p-T-miR-7) vector, the promoter region of TTF-1 (NM-7080) was amplified from DNA derived from 95D cells using a forward primer (5′-CGGGGTACCTGTTTCGGCAACTAC) and a reverse primer (5′-CGACGCGTCCTTCT--GGGTCCTT) and subcloned into *Kpn*I and *Mul I* sites of p-miR-7 vector. Clone identity was verified using restriction digest analysis and plasmid DNA sequencing. Endotoxin-free plasmids were obtained using Endofree plasmid mega kit (QIAGEN GmbH, Hilden, Germany). Then, plasmids were transiently transferred into the 95D cells using Lipofectamine-2000 (Invitrogen) in different following experiments according to the manufacturer's instruction.

Real-time PCR assay

6 organs and tissues, including heart, liver, spleen, kidney, brain, and lymph nodes were collected from nude mice lung cancer model. DNAs were purified from 100 mg these indicated organs or tissues respectively. The primers were obtained from Shanghai Sangon Biological Engineering CO, and the other reagents were from TAKARA Bio Inc. Real-time PCR were performed according to the manufacturer's protocols. The following primers were used: forward:5'-AAGCCTCTTTTTCGTGGAAGT-3',reverse:5'-GGTTGGGCAGATTTTGAATG3'

Plasmid copies were quantified using the BIO-RAD CFX96 detection system (Bio-Rad Laboratories). Relative expression was calculated using the comparative threshold cycle (Ct) method. The copies of plasmid were calculated as previously description .[1,2]

Statistical analyses

The data were analyzed with GraphPad Prism 5.0 and were presented as the mean ± SD. Student's t-test was used when two conditions were compared. Probability values of <0.05 were considered significant; two-sided tests were performed.

Supplementary figure S1

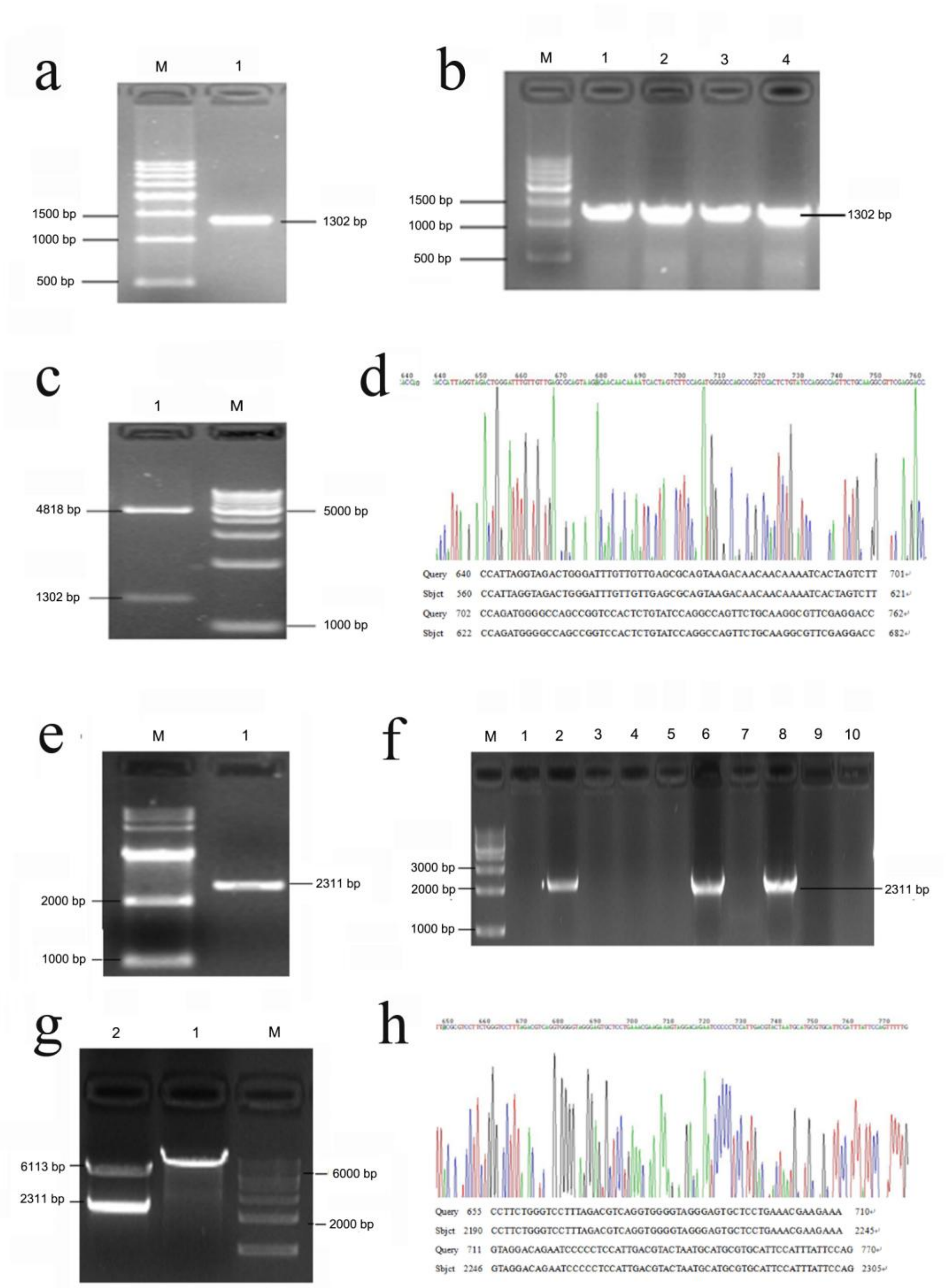

Supplement figure S1. The construction of plasmid p-T-miR-7.

miR-7 sequence were amplified from DNA derived from human lung cancer 95D cells by PCR, and then subcloned into pGL3.0 basic vector by restriction site of *Mul I* and *HindⅢ*. (**a**)The electrophoresis of pri-miR-7 (1302 bp) amplified from DNA by PCR. (**b**) The electrophoresis of PCR products of bacterium solution; 1-4: Electrophoresis of different PCR products; M: Marker; Identification of p-miR-7 plasmid by *Mul I* and *HindⅢ* double digestion(**c**) and sequencing (**d**). Then, 95D cells genomic DNA was used as template, TTF-1 promoter sequence was amplified by

PCR assay, and then cloned into p-miR-7 vector by restriction site of *Kpn I* and *Mul I* to construct PGL3.0-basic(-)-TTF-1-promoter-pri-miR-7(termed as p-T-miR-7). (**e**) The electrophoresis of PCR products of TTF-1 promoter (2311 bp) from the DNA. (**f**) The electrophoresis of PCR products of bacterium solution; 2,6,8: Electrophoresis of different PCR products; M: Marker; Identification of p-T-miR-7 plasmid by *Kpn I* and *Mul I* double digestion (**g**) and sequencing (**h**).

Supplementary figure S2

a **b**

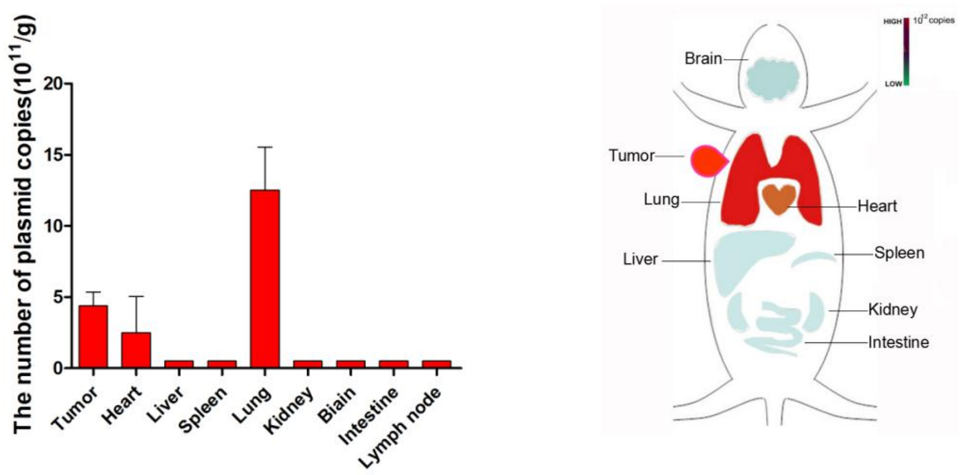

Supplement figure S2. The plasmid copy number in the main organs and tissues.

Human lung cancer cell line 95D cells were injected subcutaneously into right flank of Balb/c nude mice (n=8). 7 days later, the plasmid of p-T-miR-7(100mg) was remote given by subcutaneous injection into the left flank of nude five times every three days. 3 days after last injection, all of the mice were sacrificed and vital organs including heart, liver, spleen, kidney, brain and lymph node were harvested. The indicated DNA was purified and used for the calculation of plasmid copy number by Realtime PCR assay. (**a**) The data showed copies of plasmid in per gram organs or tissues. (**b**) The sketch map of distribution of plasmid.

Supplementary figure S3

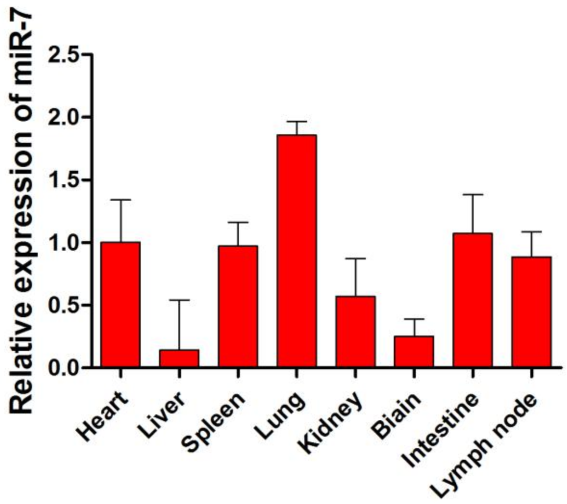

Supplement figure S3. The relative expression of miR-7.

Human lung cancer cell line 95D cells were injected subcutaneously into right flank of Balb/c nude mice (n=8). 7 days later, the plasmid of p-T-miR-7 (100mg) was remote given by subcutaneous injection into the left flank of nude five times every three days. 3 days after last injection, all of the mice were sacrificed and vital organs including heart, liver, spleen, kidney, brain and lymph node were harvested. Then, the relative expression level of miR-7 was analyzed by Realtime PCR assay. All data was normalized to heart.

Reference

[1] Ashty, S, Karim, Kathleen, A, Curran, Hal, S and Alper (2013). Characterization of plasmid burden and copy number in Saccharomyces cerevisiae for optimization of metabolic engineering applications. *FEMS Yeast Res* **13:**10.

[2]Yeon, Jeong, Son, Ae, Jin, Ryu, Ling, Li, Nam, Soo, Han and Ki, Jun, Jeong (2016). Development of a high-copy plasmid for enhanced production of recombinant proteins in Leuconostoc citreum. *Microb Cell Fact* **15**: 12.

Table S1. Over 4-fold down-regulation genes (534) in p-T-miR-7 injection group.

Trget gene	Fold change	Trget gene	Fold change	Trget gene	Fold change
RB1CC1	4.0004811	KIAA1244	4.3466776	FAM69A	5.0351474
ALDH7A1	4.0035322	lnc-CX3CR1-2	4.347025	ZCCHC6	5.0364891
AP1S1	4.0044539	MON2	4.3480297	GPAM	5.0369814
PAPOLG	4.004946	PALLD	4.3481575	RNA28S5	5.0640583
UEVLD	4.0079483	MAP9	4.3607582	GCLC	5.0918372
SLC39A1	4.0090386	C12orf29	4.3639462	GABPA	5.0993487
NMI	4.0091356	TXNDC9	4.3662175	CYP2U1	5.1121462
CD58	4.0093295	AKAP11	4.3671588	CRIM1	5.1124853
SLC39A8	4.0100366	MAP1B	4.376923	TIGD6	5.1163448
TMX1	4.0109195	PPAT	4.3782198	HAUS2	5.1170073
CBR1	4.0120512	MRPL12	4.3803404	TMEM14C	5.1240303
LOC100128593	4.0133117	PWWP2A	4.3838201	BDNF	5.1315089
TOP1	4.015285	RFK	4.3878679	MED21	5.1360545
ZFHX4-AS1	4.0230624	UQCRH	4.388114	NR3C1	5.146776
NAP1L4	4.0232364	TMEM206	4.3922516	FERMT1	5.1558826
DBF4	4.0236271	CNTD1	4.3927378	HBS1L	5.1585811
SGTB	4.0239367	ITFG1	4.3932113	AAED1	5.1615272
RNF213	4.0284339	CHCHD1	4.3948106	KPNA5	5.1650421
SAR1A	4.0286054	CAMLG	4.3951567	GALNT1	5.1791394
DNAJC8	4.0332463	CYCS	4.3995574	TUBB	5.1866571
DNAJC19	4.0386166	RPS15A	4.3998002	SEPT7	5.1869616
TERF1	4.0394778	RPS29	4.4044809	CCNT2	5.2006523
ANKRD46	4.0457504	YEATS4	4.4079015	UQCR11	5.2015659
SLC38A1	4.0486763	FAM169A	4.4106598	SIKE1	5.2101204
ZNF226	4.0527034	SHMT2	4.4131672	PRR3	5.2141027
BID	4.055145	SNRPG	4.4145549	MOB4	5.2148205
PDGFRL	4.0564638	FAM105A	4.4182045	TTK	5.2190166
SCAMP1	4.058141	PDIA3	4.424181	ADAT2	5.220592
SDHD	4.0639397	TAF13	4.4394101	LARP1B	5.2320448
IFIT5	4.0648252	SPCS2	4.4418464	XRCC6BP1	5.2335359
ALDH9A1	4.0701304	COPS8	4.442077	EPC1	5.2382358
CDH11	4.0715886	CLGN	4.4430941	PFN2	5.2403312
OSTF1	4.0752117	LIMA1	4.4503719	TPK1	5.2438936
YIPF5	4.0770612	ZBTB41	4.4625722	ASXL3	5.2496127
PHYHIPL	4.0779565	ARPP19	4.4644115	LAMP2	5.2499006
PDE12	4.0805881	MRPL14	4.4652285	TUBE1	5.2602505
CKS2	4.0824183	GGCX	4.4665874	ZNF396	5.2811016
LINC00597	4.0828569	SERPINB9	4.4700492	PTAR1	5.3097543
SNRPE	4.084074	DENND1B	4.4721761	PLA2G4A	5.3204708
MIR155HG	4.0867279	PLSCR4	4.4723237	SRP72	5.3227264
RNF2	4.0883735	NRIP1	4.4746592	MRPL13	5.3232193
DTD1	4.0924534	TMED2	4.476276	BID	5.3360282
C11orf58	4.0958997	MYCL	4.4764054	ARL5B	5.3383445
NCAM2	4.0968952	DPY19L2P2	4.4819607	GDI2	5.3388718
CDK1	4.0976521	TSNAX	4.4825066	KIF20B	5.3422926
NAA15	4.100482	TMEM126A	4.4842695	PLAC8	5.3463712
REEP3	4.1006181	PFDN4	4.4843814	FAM127C	5.3473199
ANG	4.101393	LINC00944	4.4935844	ALCAM	5.3509365
EAF2	4.101824	EPHA7	4.5006863	TMTC3	5.3733838
SETD7	4.1029706	GPR126	4.5015162	HSD17B11	5.3752394
ZNF254	4.1030846	COMMD10	4.5061302	MRPL12	5.3907821
KBTBD3	4.103441	PTGIS	4.5075934	TRUB1	5.3974826
PEX3	4.1053294	USP37	4.5104582	ANKRD31	5.4167143
IER3IP1	4.1110485	FUOM	4.5122925	DNM1L	5.425204
EPM2AIP1	4.1110499	HIAT1	4.5141489	OSTM1	5.4316605
GNPDA2	4.1119223	POLR1B	4.5143085	SNORA53	5.4380145
P2RY2	4.1133345	SLC7A11	4.5145901	C6orf57	5.4441899
HSP90AA1	4.1143628	NANP	4.5150971	KIF21A	5.4677868

Continued

Trget gene	Fold change	Trget gene	Fold change	Trget gene	Fold change
MCU	4.1149503	NDUFB6	4.5160548	PLS3	5.4695836
KIAA0825	4.1199685	LAMTOR3	4.5209263	BMPR2	5.4854744
RNU12	4.1214757	LRRIQ3	4.522189	SLC25A46	5.4916683
C10orf32	4.1260843	CASC5	4.5258802	RGS5	5.5015849
SLC35E3	4.1271017	SPECC1	4.5363288	AREG	5.502504
RAB8B	4.1273489	NUP98	4.5376596	LOH12CR1	5.5064992
HACL1	4.1274078	DR1	4.5404356	CCDC132	5.5348047
PIGP	4.1293554	KLHL7	4.5413631	TOR1AIP2	5.5424099
ZNF678	4.1308965	CENPA	4.5511199	NUP37	5.5868396
SLC16A1	4.132257	ENPP1	4.5550251	PJA2	5.5926227
TAB3	4.1323418	GALC	4.5575737	NUDT14	5.5959951
NDUFA4	4.1337181	ISOC1	4.5584078	SEL1L	5.6111108
RPS15A	4.1353258	SNORA23	4.5589766	RBM43	5.6274499
NDUFA11	4.141982	GSKIP	4.5606675	SLC31A1	5.6392426
KCTD12	4.1436553	SEC24D	4.5629603	MLF1	5.6467509
METTL21B	4.1471792	CASP8	4.568781	NT5DC1	5.6498732
SELT	4.1473184	KRIT1	4.5727226	GPR22	5.6614947
ZNF91	4.1491804	CMC1	4.5761806	MCTP1	5.6621442
CRYZ	4.1508661	PPIL1	4.5788554	SLC10A4	5.6690017
HSPA13	4.1542931	PIGK	4.5803537	HAVCR2	5.6755519
CTBS	4.1547962	CLDN1	4.581701	SHROOM3	5.6941914
THAP2	4.1576396	LYPLA1	4.5866938	TMED7-TICAM2	5.6985703
CCNB1	4.1589144	ST8SIA4	4.6039941	TMEM14A	5.7072292
DNAJC19	4.1597246	SLC25A46	4.6074257	OTUD6B	5.7202992
CANX	4.159835	BAG2	4.6101702	NAT1	5.7249199
CACYBP	4.1659427	SELT	4.6131609	CAP2	5.7308741
NPAT	4.1670332	NXT2	4.6160697	NLGN1	5.7317342
TCF12	4.1672539	UFM1	4.6193697	ANTXR1	5.7548128
BMPR1A	4.1708968	PRNP	4.6195586	RPL26L1	5.8384402
ESD	4.1715513	MDH1	4.6213809	RRM1	5.8552388
STRN	4.1770621	B9D1	4.6237785	GATC	5.8761198
GPR180	4.1788228	SMAD1	4.6240196	NQO1	5.9320905
RPL39	4.1791311	DSG2	4.6247424	HSPA4L	5.9581465
DHCR24	4.179696	P2RY1	4.6274686	C17orf104	5.9605328
RPL13AP3	4.1799596	ARL6IP5	4.6354757	HSD17B7	5.9710183
COX16	4.1827797	CRYZ	4.6441449	DBF4	5.9711909
TMEM123	4.183914	E2F7	4.64554	CKS1B	5.983232
SPRYD7	4.186569	DPH3	4.6482511	BBS10	6.0043955
TSPAN12	4.1873172	FLJ40536	4.6486787	KBTBD7	6.0282239
RBBP4	4.1899761	SNRPE	4.6501975	ZNF705A	6.0626553
LOC389831	4.193213	PHACTR2	4.6504119	MRPL42	6.0695729
TFPI	4.1939812	SPOCK3	4.6589337	FSIP2	6.0978772
TMTC2	4.1949058	PTPLAD1	4.665446	ATP5C1	6.1184024
SAP30	4.2004256	CHMP5	4.6710449	ATP7A	6.1240379
PCDHB9	4.2031674	SELM	4.675796	PIGK	6.1493874
CCDC186	4.2054809	TMLHE	4.6761182	TMEM38B	6.1752652
DDB1	4.2067235	PYROXD1	4.6780533	GLS	6.17571
LCOR	4.2068361	TMED5	4.6804946	SNORA59B	6.2214433
HLTF	4.2111667	TMEM14C	4.6810624	MRPS14	6.2243794
QSER1	4.2112339	PHF5A	4.6833621	FAHD1	6.2329166
TERF1	4.2162763	LAMTOR5	4.6841166	CHRNA5	6.2700446
SENP7	4.218407	SQRDL	4.6841426	ATP7A	6.2799497
IL6ST	4.2202311	TP53	4.6842024	WDFY3	6.29795
DACH1	4.2213861	LSM3	4.6865556	HHEX	6.3054372
EMB	4.2226185	CCDC121	4.689615	POMP	6.3258178
CLN5	4.2234501	DEF8	4.6917625	ESCO2	6.3962361
UTY	4.2242037	IL7	4.7021645	ARHGAP18	6.3977814
PSMA4	4.2264928	EPS8	4.7160228	ACSL3	6.4274055
SPIN4	4.2290218	C2orf88	4.7167682	GOLT1B	6.4374334
SLC39A9	4.2296934	COX5A	4.7227494	PPM1A	6.4660022

Continued

Trget gene	Fold change	Trget gene	Fold change	Trget gene	Fold change
TROVE2	4.2302414	PLAA	4.7274638	CSRNP1	6.480614
FLJ10038	4.2308748	SNRPE	4.7282293	TRMT1L	6.4819729
UBLCP1	4.2348416	HOMER1	4.7323212	SGOL2	6.4859235
LEPR	4.2356671	SEP15	4.7327345	PLCB1	6.5323469
SMIM11	4.2359689	CHD9	4.7368162	CTSO	6.5413568
INPP4B	4.2373001	NDUFA13	4.7436218	SLC39A10	6.5608808
CLIC4	4.2375494	NID2	4.7440493	TMEM14B	6.5609558
LINC01503	4.2381778	DLEU2L	4.7860352	AHR	6.5813781
LYPLAL1	4.2397538	FKBP14	4.7885438	TMEM106B	6.5843421
FCF1	4.2402255	KCNG3	4.7889504	EDEM3	6.6796888
ZNF720	4.2442855	MREG	4.7917764	C8orf88	6.7013572
TP53TG1	4.2477139	CROT	4.7921896	CPE	6.7260197
STXBP4	4.2496647	COMMD8	4.8076684	BPNT1	6.82355
ATXN10	4.2500214	PPM1L	4.8147277	DNAJC6	6.8529302
PHYH	4.2522093	OSBPL8	4.8157033	ERAP1	6.8606582
C2orf73	4.2527894	IL6ST	4.8157941	LMBR1	6.9024953
AARD	4.2531042	MRPL54	4.8167642	LEPROT	6.9424631
ZDHHC20	4.254398	SLC30A6	4.8176884	CHMP2B	7.0466424
ARSK	4.255253	CTDSPL2	4.8195929	MRPL1	7.0622143
GABARAPL1	4.2564468	MDH1	4.8278505	GULP1	7.073793
ITGB3	4.2582156	WDFY1	4.8299183	TMTC3	7.0817602
HOXA13	4.2586643	SDHD	4.8306174	TMPO	7.1223632
TM2D1	4.2597374	ELMOD2	4.8370351	SULT2B1	7.1726145
PAK2	4.2599072	GSTO1	4.8399797	ITGA4	7.268674
TSPAN14	4.2609969	FNIP1	4.8441314	PLS1	7.2696419
PIGN	4.2615297	COL8A1	4.8548795	NUDCD1	7.3693532
GABPB1	4.263449	POT1	4.8595917	C11orf58	7.4399344
HNRNPCL1	4.2655359	GULP1	4.8646287	CHCHD2	7.4777539
BIK	4.2656352	GSR	4.8659598	CAPN7	7.5704807
ITGAV	4.2692422	PTN	4.8729043	TRMT10A	7.5948838
POLR3G	4.2712964	SEC23A	4.8737694	MANEA	7.7068136
UQCRH	4.2724452	C15orf48	4.8746208	BFSP2	7.7398328
TIPARP	4.2737911	C11orf70	4.8765069	CNRIP1	8.1045889
SFMBT2	4.2762571	LINC01300	4.8774409	LRP12	8.1352462
HS2ST1	4.2815999	MRPS28	4.8954518	FGF12	8.1820253
SPINT2	4.2816655	CNIH1	4.8976681	RBM24	8.2989781
HIST1H4A	4.2816712	MGST3	4.9045882	FZD7	8.3067556
C2orf76	4.2841254	UMAD1	4.9051559	PRKACB	8.3318271
CERS6	4.2866006	CMTM6	4.9155563	TMEM17	8.383965
CASP3	4.2956129	TUBB	4.9252829	PHF14	8.3991539
SNRPG	4.2985855	FBXO22	4.9300323	EDIL3	8.6604477
SSX2IP	4.300463	TMEM56	4.9309205	TMEM106B	8.8020953
DTWD1	4.3004934	HOOK3	4.9451459	TLE4	8.8432238
ARL6IP1	4.3044443	DPH6	4.9558403	ACAP2	8.9732276
DLEU2L	4.3053335	HINT1	4.9558448	ATP6V1C2	8.9737867
PGM2	4.3055126	LOC100131048	4.9653899	FAM171B	9.0195703
NAPEPLD	4.3063692	LZTFL1	4.9710741	AKAP9	9.4973986
TMEM135	4.3069811	EIF5A2	4.9745703	RBBP9	9.5211247
PDLIM3	4.307655	C1QBP	4.9775679	PIGM	9.6391839
COMMD6	4.3084771	DUSP19	4.9913532	TMSB15A	9.6602271
PARVA	4.3094845	PRRC1	4.9961899	FAM198B	9.6818188
ECHDC1	4.3106248	RPA3	5.0036886	SLC30A7	9.805271
GPX8	4.315794	JAZF1	5.00808	GTF3C3	10.02813
ZC3HAV1	4.3208603	METTL20	5.0131701	DNAJB9	10.1163125
VPS41	4.3261302	SNRPG	5.015446	FSTL5	10.1447092
PCYOX1	4.3275065	SIVA1	5.0180401	CDC73	11.4744763
ZHX1	4.3312805	ARHGAP5	5.0198237	C17orf104	11.9460056
C3orf58	4.3333552	LAMP3	5.0213473	TMEM38B	13.1177293
ANLN	4.339484	DEPDC1	5.0233208	XK	15.5924897
TMEM19	4.3415554	CD302	5.0273091	HIST1H4C	15.6153013
