# Supplementary material for: Targeted Expression of miR-7 Operated by TTF-1 Promoter Inhibited the Growth of Human Lung Cancer through the NDUFA4 Pathway
Source: Mol Ther Nucleic Acids. 2016 Dec 31;6:183–97. doi: 10.1016/j.omtn.2016.12.005 (PMC5363496; doi:10.1016/j.omtn.2016.12.005)
Supplement: Document S2. Article plus Supplemental Information [file mmc2.pdf]

Molecular Therapy
Nucleic Acids
Original Article

AMERICAN SOCIETY of GENE & CELL THERAPY

Targeted Expression of miR-7 Operated by TTF-1 Promoter Inhibited the Growth of Human Lung Cancer through the NDUFA4 Pathway

Liangyu Lei,[1,3] Chao Chen,[1,3] Juanjuan Zhao,[1] HaiRong Wang,[1] Mengmeng Guo,[1] Ya Zhou,[2] Junming Luo,[1] Jidong Zhang,[1] and Lin Xu[1]

[1]Department of Immunology, Zunyi Medical College, Guizhou 563000, China; [2]Department of Medical Physics, Zunyi Medical College, Guizhou 563000, China

Targeted expression of gene technique is an important therapeutic strategy for lung cancer. MicroRNA-7 has been well documented as a promising tumor suppressor but never been test in specific gene-promoter-targeted expression in cancer gene therapy. Here, we first evaluated the efficacy of miR-7 expression operated by the promoter of TTF-1, a lineage-specific oncogene in lung cancer, in vitro using an eukaryotic vector of TTF-1-promoter-operated expression of miR-7 (termed as p-T-miR-7). Interestingly, using a nude mice model, the growth and metastasis of human lung cancer cells in vivo were significantly reduced in remote hypodermic injection of the p-T-miR-7 group, accompanied by increased expression of miR-7 and reduced transduction of the Akt and Erk pathway in situ. Mechanism aspect, global gene expression analysis showed that downregulation of NDUFA4, a novel target of miR-7, contributed to the effects of miR-7 expression operated by TTF-1 promoter on the growth and metastasis of human lung cancer cells, as well as altered transduction of the Akt and Erk pathway. Finally, there was no significant difference in weight or histopathology of other organs. These data provided a basis for development of novel modality of miRNA-based targeted expression therapy against clinical lung cancer.

INTRODUCTION

Lung cancer is one of the most common causes of cancer death. The overall 5-year survival rates for surgical resection in lung cancer patients still remain poor. Thus, it is important to identify and characterize new molecular markers and genes to develop more targeted treatment strategies to improve the clinical outcome of lung cancer.[1–3] Recent evidence showed that the technique of targeted gene expression, including specific gene-promoter-operating expression and distinct delivery systems, is a crucial strategy for gene therapy against various cancers including lung cancer.[4–7] And, microRNAs (miRNAs), which are endogenous 21- to 23-nt non-coding RNAs, have pointed to central regulatory roles in the development of lung cancer and emerged as important targets for gene therapy against clinical lung cancer.[8–11] This research suggested that targeted expression of distinct miRNA molecules might be a useful therapeutic strategy for lung cancer, which would be ultimately benefit to the outcome of clinical lung cancer patients.

MicroRNA-7 (miR-7), a unique member of miRNAs, played an important role in the progression of various tumors including lung cancer.[12–14] For lung cancer, accumulating evidence suggested that miR-7 was an important regulator in the development of lung cancer through controlling the growth and invasion, as well as apoptosis, of lung cancer cells and emerged as a novel potential therapeutic target. For example, Xiong et al.[15] found that overexpression of miR-7 suppressed NSCLC cells proliferation and migration in vitro, and reduced tumorigenicity in vivo. At the same time, Li et al.[16] showed that restoration of miR-7 expression suppressed the tumorigenicity of lung cancer cells in vivo. Consistently, our previous work also showed that overexpression of miR-7 could reduce the growth and metastasis of human lung cancer cells in vivo and in vitro.[17] Moreover, the reduced expression of miR-7 was associated with the sites mutation of its promoter region in lung cancer tissues, indicating that miR-7, an important tumor suppressor, could be used as candidate for targeted gene therapy against lung cancer.[18,19]

Thyroid transcription factor (TTF)-1 is a member of the homeodomain-containing Nkx2 family of transcription factors. Recent evidence showed that TTF-1, as a lineage-specific oncogene, was dominantly expressed in lung cancer, but not other types of cancers, and its expression level was closely correlation with the prognosis of lung cancer patients.[20–23] These findings raise an interesting question whether promoter of TTF-1 gene might be an ideal candidate for therapeutic strategy of targeted expression of distinct genes in lung cancer, which remains to be elucidated. Therefore, in this present study, we first constructed an eukaryotic vector of promoter of TTF-1-gene-operating expression of miR-7 (termed as p-T-miR-7) and observed its effects on the growth and migration of human lung cancer cells in vitro. Moreover, we further evaluated the potential effect of the TTF-1-promoter-operating miR-7 expression on the growth and metastasis of human lung cancer cells in vivo. Of note, we found

Received 14 October 2016; accepted 5 December 2016;
http://dx.doi.org/10.1016/j.omtn.2016.12.005.

[3]These author contributed equally to this work.

Correspondence: Lin Xu, Department of Immunology, Zunyi Medical College, Guizhou 563003, China.
E-mail: xulinzhouya@163.com

CrossMark

This is an open access article under the CC BY license (http://creativecommons.org/licenses/by/4.0/).

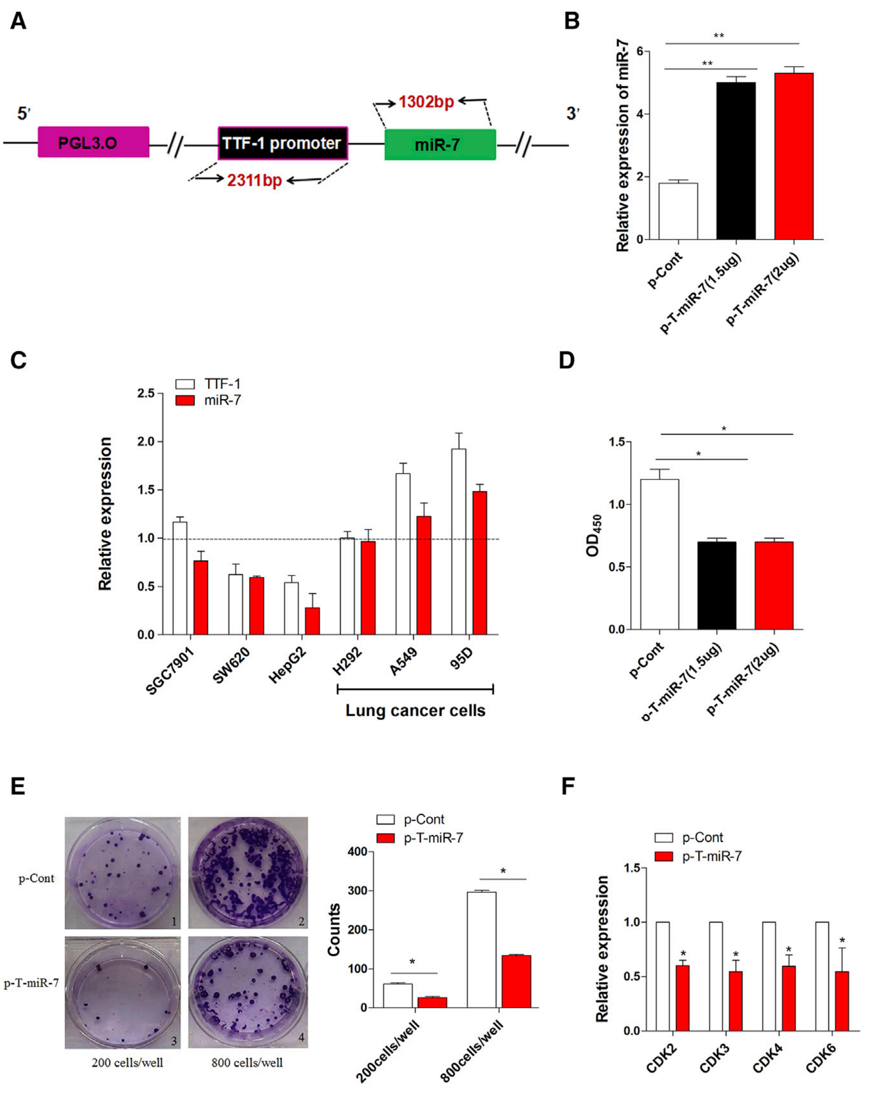

Figure 1. TTF-1-Promoter-Operating miR-7 Expression Inhibited the Growth of Human Lung Cancer Cells In Vitro

(A) The schematic of an eukaryotic expression vector (termed as p-T-miR-7). (B) Human lung cancer cell line 95D cells were transiently transfected with p-T-miR-7 (1.5 or 2 μg) or p-Cont (2 μg) in vitro. 48 hr later, cells were collected, and the expression level of miR-7 was detected by real-time PCR assay. (C) The plasmid of p-T-miR-7 (2 μg) was transiently transfected into human lung cancer cell line 95D cells, A549 cells, NCI-H292 cells, gastric cancer cell line SGC901 cells, hepatic cancer cell line HepG2 cells, and colon cancer cell line SW620 cells, respectively. After 48 hr, the expression level of miR-7 and TTF-1 was analyzed by real-time PCR assay. The relative expression of miR-7 was normalized to the corresponding control group. (D) Human lung cancer cell line 95D cells were transiently transfected with p-T-miR-7 (2 μg) or p-Cont (2 μg) in vitro. 48 hr later, the proliferation of 95D cells was detected by CCK-8 assay. (E) The colony-formation assay was performed, and the colon numbers were calculated. (F) Human lung cancer cell line 95D cells were transiently transfected with p-T-miR-7 (2 μg) or p-Cont (2 μg) in vitro. 48 hr later, the expression of indicated cell-growth-related molecules was detected by real-time PCR assay. Representative data of three independent experiments are shown. $^{*}p < 0.05$, $^{**}p < 0.01$.

that overexpression of NDUFA4, a novel target molecule of miR-7, could abrogate the effect of TTF-1-promoter-operating miR-7 expression on the growth of lung cancer cells, accompanied with altered expression of phosphorylation of Akt and Erk. Thus, our work showed for the first time that TTF-1-promoter-operating miR-7 expression might be an ideal strategy for targeted expression of miR-7 in lung cancer, which provides preliminary experimental basis for targeted expression of distinct miRNAs in lung cancer and was helpful for the development of gene therapy against clinical lung cancer.

RESULTS

TTF-1-Promoter-Operating miR-7 Expression Suppressed the Growth of Human Lung Cells In Vitro

To investigate whether the TTF-1 promoter might be an ideal candidate for operating miR-7 expression in lung cancer, as shown in Figure 1A, we first amplified and inserted the sequence of both TTF-1 promoter and miR-7 into pGL3.0 basic vector and successfully constructed an eukaryotic expression vector, which could express miR-7 operated by TTF-1 promoter (termed as p-T-miR-7) (Figure S1). In order to test the efficiency of TTF-1-promoter-operating miR-7 expression, we then transiently transfected plasmid p-T-miR-7 into human lung cancer cell line 95D cells. Real-time PCR assay showed that the expression level of the miR-7 in p-T-miR-7 transfection group was unmistakably higher than that in the control group (Figure 1B; $p < 0.05$), indicating TTF-1 promoter could effectively operate the expression of miR-7 in lung cancer cells. Next, to observe the target efficiency, we further transfected the plasmid p-T-miR-7 into six different human cancer cell lines, including lung cancer cell line 95D cells, A549 cells, NCI-H292 cells, gastric cancer cell line SGC901 cells, hepatitic cancer cell line HepG2 cells, and colon cancer cell line SW620 cells, and then detected the expression level of miR-7 operated by TTF-1 promoter. Interestingly, we found that the expression level of miR-7 was higher in lung cancer cells, including 95D cells, A549 cells, and NCI-H292 cells, than in other types of tumor cells (Figure 1C; $p < 0.05$), indicating higher intrinsic activity of TTF-1 promoter in lung cancer cells. To verify this phenomenon, we also detected the expression of TTF-1 in these cancer cells, which also valuably reflected the intrinsic activity of TTF-1 promoter. Consistently, we found that the

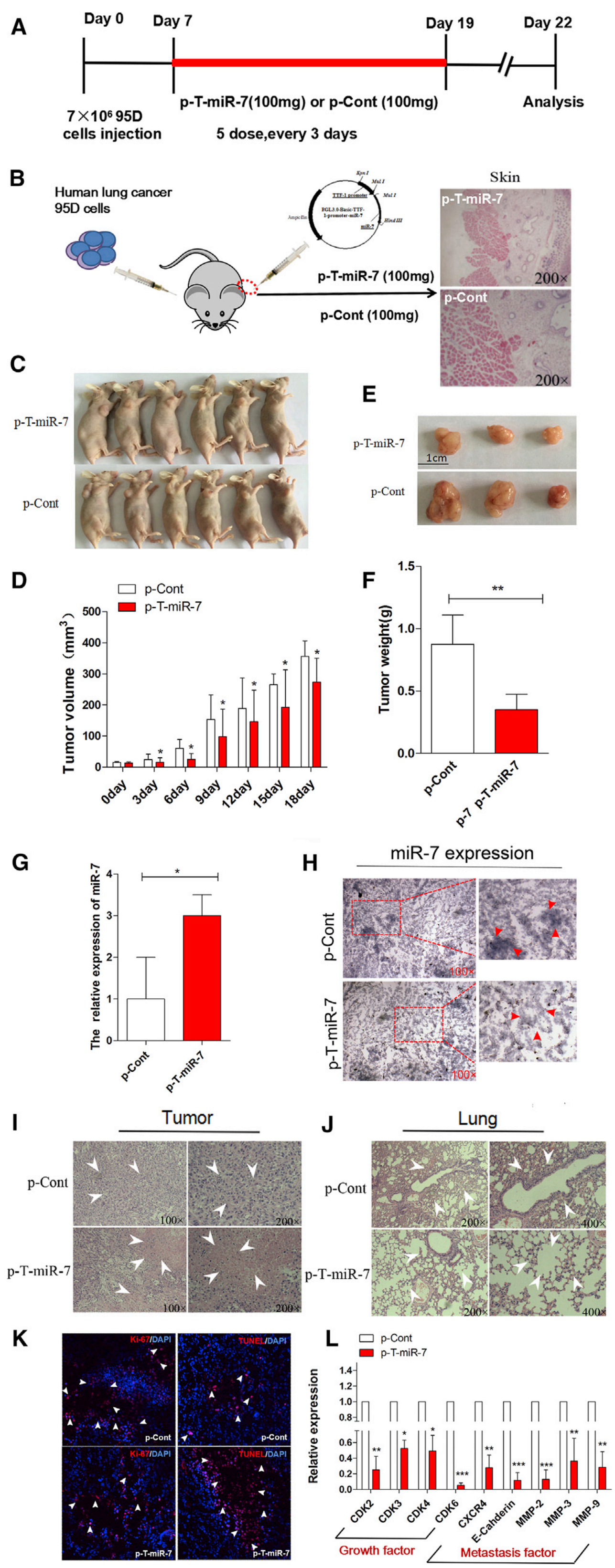

Figure 2. TTF-1-Promoter-Operating miR-7 Expression Suppressed Tumorigenesis of Lung Cancer In Vivo

(A) The schedule of study design. (B) Human lung cancer cell line 95D cells were injected subcutaneously into right flank of BALB/c nude mice (n = 8). 7 days later, the plasmid of p-T-miR-7 (100 mg) or p-Cont (100 mg) was remotely given by subcutaneous injection into the left flank of nude mice five times every 3 days. 3 days after last injection, all mice were killed. The skin of injection sections was stained with H&E and observed by microscope (magnification 200×). And partial mice were imaged (C). (D) The growth curve of tumor. (E) The representative morphology and (F) weight of tumor. The expression of miR-7 in tumor tissue was detected by real-time PCR assay (G) and in situ hybridization assay (H). H&E staining of tumor tissue (I) and lung tissue (J). (K) The ability of proliferation (Ki-67) and apoptosis (TUNEL) in tumor tissue were analyzed by immunofluorescence assay. (L) The expression of cell-growth-related molecules (CDK2, CDK3, CDK4, and CDK6) and metastasis-related molecules (CXCR4, E-Cadherin, MMP2, MMP3, and MMP9) in tumor tissue was detected by real-time PCR assay. *$p < 0.05$, **$p < 0.01$. ***$p < 0.001$.

expression level of TTF-1 was also higher in lung cancer cells than that in other cancer cells, which was consistent with previous finding that TTF-1 was dominantly expressed in lung cancer cells, but not other types of cancer cells.[24–26]

Accumulating literatures have documented that miR-7, as a promising tumor suppressor, could regulate the biological behavior of human lung cancer cells.[1,12,13] Moreover, our previous research works also showed that overexpression of miR-7 could inhibit the growth of human lung cancer cells.[17,18] Therefore, we further investigated whether expression of miR-7 operated by TTF-1 promoter could affect the growth of human lung cancer cells in vitro. As shown in Figure 1D, the proliferation of human lung cancer cell line 95D cells decreased significantly in the p-T-miR-7-transfected group ($p < 0.05$). Moreover, the colon-formation ability of cells was also impaired unmistakably (Figure 1E; $p < 0.05$), which was consistent with our previous works.[17,18] To confirm this finding, we also detected the expression of cell-growth-related factors such as CDK2, CDK3, CDK4, and CDK6 in lung cancer cells. As shown in Figure 1F, the relative expression of these CDK family members also decreased significantly in the p-T-miR-7-transfected group compared with those in the control group ($p < 0.05$). Combining these data demonstrated that miR-7 could be effectively targeted expression by the TTF-1 promoter and suppressed the growth of human lung cells in vitro.

TTF-1-Promoter-Operating miR-7 Expression Inhibited Tumorigenicity In Vivo

To further explore the potential therapeutic effect of TTF-1-promoter-operating miR-7 expression on tumorigenicity in vivo, a xenograft model of human lung cancer in nude mice was adopted. Human lung cancer cell line 95D cells, which is a highly tumorigenic and metastatic cell line, were injected subcutaneously into right flank of nude mice. 7 days later, the plasmid of p-T-miR-7 or p-Cont was remotely given by subcutaneous injection into the left flank of nude

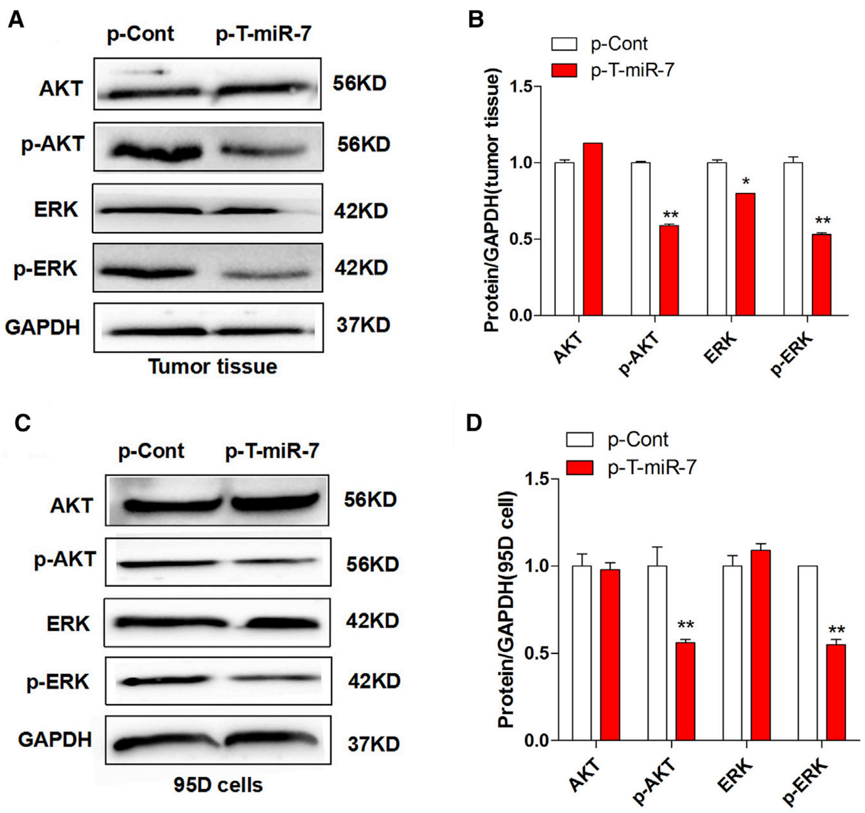

Figure 3. TTF-1-Promoter-Operating miR-7 Expression Altered the Transduction of the Akt and Erk Signaling Pathway

Human lung cancer cell line 95D cells were injected subcutaneously into right flank of BALB/c nude mice (n = 8). 7 days later, the plasmid of p-T-miR-7 (100 mg) or p-Cont (100 mg) was remotely given by subcutaneous injection into the left flank of nude mice five times every 3 days. 3 days after last injection, tumor mass was collected. The protein level of Akt, p-Akt, Erk, and p-Erk was detected by western blotting (A) and calculated (B). Human lung cancer cell line 95D cells were transiently transfected with p-T-miR-7 (2 μg) or p-Cont (2 μg) in vitro. 48 hr later, cells were collected, and the protein level of Akt, p-Akt, Erk, and p-Erk was detected by western blotting (C) and calculated (D). $^{*}p < 0.05$, $^{**}p < 0.01$. Representative data of three independent experiments are shown.

mice five times every 3 days (Figures 2A and 2B). Then, the growth of tumor was observed accordingly. As shown in Figures 2C–2F, both the volume and weight of tumor tissue in the p-T-miR-7 injection group were reduced significantly compared with those in the p-Cont injection group ($p < 0.05$). Importantly, we analyzed the expression level of miR-7 in tumor tissue and found that the expression level of miR-7 in the p-T-miR-7 injection group was significantly higher than that in the p-Cont injection group (Figure 2G; $p < 0.05$). To confirm these data, we further detected the expression of miR-7 in tumor mass by in situ hybridization and obtained a similar result (Figure 2H).

Next, we further monitored the impact of TTF-1-promoter-operating miR-7 expression on lung cancer cells in vivo. As shown in Figure 2H, H&E staining showed that there were large areas of necrosis in tumor tissue in the p-T-miR-7 injection group. Moreover, the metastatic index of lung also decreased significantly (Figure 2J; $p < 0.05$), indicating TTF-1-promoter-operating miR-7 expression also could significantly inhibit the metastasis of lung cancer cells in vivo, which was consistent with our previous report.[17] Immunofluorescence assay further showed that the proliferation of tumor cells decreased significantly in the p-T-miR-7 injection group (Figure 2L; $p < 0.05$). On the contrary, the apoptosis of cells increased unmistakably (Figure 2L; $p < 0.05$). Finally, to confirm the effect of TTF-1-promoter-operating miR-7 expression on the growth and metastasis of tumor cells in vivo, we further detected the expression of cell-growth-related molecules including CDK2, CDK3, CDK4, and CDK6, as well as metastasis-related molecules including CXCR4, E-Cadherin, MMP2, MMP3, and MMP9, in tumor mass, respectively. Data showed that the expression of all of these molecules in the p-T-miR-7 injection group decreased unmistakably (Figure 2I; $p < 0.05$). All of the above data demonstrated that TTF-1 promoter could effectively operate miR-7 expression in tumor mass, which subsequently inhibited tumorigenicity of lung cancer in vivo.

TTF-1-Promoter-Operating miR-7 Expression Altered the Transduction of the Akt/Erk Pathway

An increasing body of literatures documented that miR-7 could regulate the growth of lung cancer cells though various signal pathways such as the Akt and Erk pathway.[27–29] Our previous work also showed that miR-7 could inhibit the proliferation and metastasis of lung cancer cells though the Akt pathway.[17] Thus, to further verify the effect of TTF-1-promoter-operating miR-7 expression on the growth of lung cancer cells, we analyzed the expression of phosphorylation of Akt and Erk in tumor tissue derived from the p-T-miR-7 injection group or the p-Cont injection group, respectively. Data showed that there were not any changes on the expression level of both Akt and Erk in between the p-T-miR-7 injection group and the p-Cont injection group. However, the expression level of phosphor-Akt and phosphor-Erk were decreased significantly in the p-T-miR-7 injection group (Figures 3A and 3B; $p < 0.05$). To confirm these findings, we also transiently transfected p-T-miR-7 or p-Cont into lung cancer cell line 95D cells in vitro, respectively, and found the expression level of both phosphor-Akt and phosphor-Erk were decreased significantly in the p-T-miR-7-transfected group compared with those in the p-Cont-transfected group

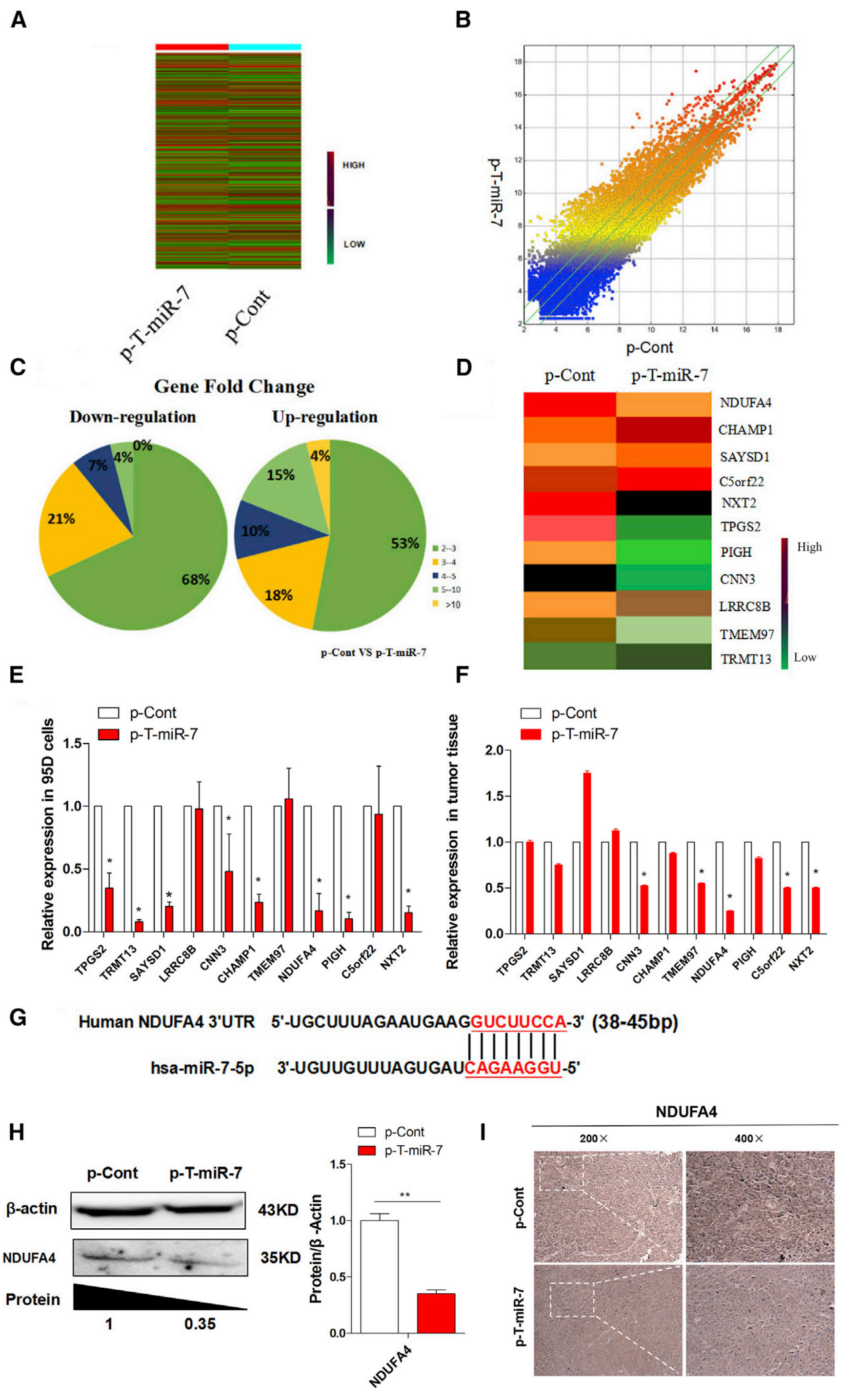

Figure 4. TTF-1-Promoter-Operating miR-7 Expression Reduced the Expression of NDUFA4

Human lung cancer cell line 95D cells were injected subcutaneously into right flank of BALB/c nude mice (n = 8). 7 days later, the plasmid of p-T-miR-7 (100 mg) or p-Cont (100 mg) was remotely given by subcutaneous injection into the left flank of nude mice five times every 3 days. 3 days after last injection, tumor mass were collected. The global gene expression was analyzed by cDNA chip array. (A) Heatmap and (B) scatterplot of gene expression. (C) The fold change and frequency. (D) Prediction of 11 target genes, including NXT2, C5orf22, PIGH, NDUFA4, TMEM97, CHAMP1, CNN3, LRRC8B, SAYSD1 and TRMT13, and TPGS2, by using miRBase and Targetscan software. (E) The expression of these indicated genes were determined by real-time PCR assay. (F) Human lung cancer cell line 95D cells were transiently transfected with p-T-miR-7 (2 μg) or p-Cont (2 μg) in vitro. 48 hr later, cells were collected, and the expression of the indicated genes were determined by real-time PCR. (G) Putative miR-7-binding sites in the 3′ UTR of human NDUFA4. (H) The protein level of NDUFA4 in tumor tissue was analyzed by western blotting and (I) immunohistochemical staining, respectively (original magnification 100×). *$p < 0.05$, **$p < 0.01$.

(Figures 3C and 3D; $p < 0.05$). These results suggested that TTF-1-promoter-operating miR-7 expression attenuated the growth of human lung cells in vitro and in vivo by altering the transduction of the Akt/Erk pathway, which was consistent with our previous findings.[17,30]

TTF-1-Promoter-Operating miR-7 Expression Reduced the Expression of NDUFA4

In order to explore the underlying mechanism of TTF-1-promoter-operating miR-7 expression on the growth of lung cancer cells, we analyzed the global gene expression profile in tumor tissue between the p-T-miR-7 and the p-Cont injection group using gene expression microarray assay. The altered gene expression profiles in p-T-miR-7 were shown in a heatmap (Figures 4A and 4B). Given a 4-fold change in differential expression as a cutoff, 568 genes were upregulated, and 534 of them were downregulated (Figure 4C; Table S1). To further elucidate the potential molecular mechanism through which TTF-1-promoter-operating miR-7 expression affected the growth of lung cancer cells, we used miRBase and TargetScan software to compare the downregulated genes in the p-T-miR-7 injection group and found 11 putative miR-7 target genes, including NXT2, C5orf22, PIGH, NDUFA4, TMEM97, CHAMP1, CNN3, LRRC8B, SAYSD1, TRMT13, and TPGS2 (Figure 4D), which also were closely related to tumor cell growth according to previous literatures.[31–34] Then, we verified the expression of these 11 predicted target genes, respectively. Unexpectedly, real-time PCR assay showed that only NDUFA4, one target among all predicted target genes of miR-7,

was significantly downregulated more than five times both in tumor tissue in the p-T-miR-7 injection group (Figure 4E; $p < 0.05$) and in p-T-miR-7-transfected tumor cells, respectively (Figure 4F; $p < 0.05$). Further analysis showed that miR-7 could bind to the 3′ UTR region of NDUFA4 mRNA (Figure 4G). Importantly, western blotting assay further showed that the level of NDUFA4 protein was significantly decreased in tumor tissue in the p-T-miR-7 injection group compared with that in the p-Cont injection group (Figure 4H; $p < 0.05$). Moreover, we also performed immunohistochemistry assay to detect the expression of the NDUFA4 protein in tumor tissue and obtained a similar result (Figure 4I). In addition, luciferase assay also showed that miR-7 could bind to the 3′ UTR region of NDUFA4 mRNA (data not shown). Collectively, our data indicated that TTF-1-promoter-operating miR-7 expression could affect the growth of tumor cells, which might be closely due to the downregulation expression of NDUFA4.

Overexpression of NDUFA4 Promoted the Proliferation and Metastasis of Human Lung Cancer Cells

Previous works showed that NDUFA4, a subunit of complex IV of the mammalian electron transport chain, played an important role in the development of cancers, such as renal cell carcinoma.[35,36] However, the knowledge on the potential role of NDUFA4 in the development of lung cancer is still limited. In order to explore whether NDUFA4 played a potent biological role in the growth of lung cancer cells, we constructed an eukaryotic expression vector of NDUFA4 (termed as p-NDUFA4) (Figure 5A) and then transiently transfected p-NDUFA4 into human lung cancer cell line 95D cells in vitro (Figure 5B). Expectedly, real-time PCR assay showed that the expression level of NDUFA4 significantly increased in the p-NDUFA4 transfection group compared with the control group (Figure 5C). Importantly, we found that both the proliferation and migration ability of 95D cells were elevated (Figures 5D and 5E; $p < 0.05$). Consistent with these findings, the clone-forming ability of cells was also promoted (Figure 5F; $p < 0.05$).

To further verify the role of NDUFA4 in the growth and metastasis of lung cancer cells, we detected the expression of cell-growth-related molecules including CDK2, CDK3, CDK4, and CDK6, as well as metastasis-related molecules including CXCR4, E-Cadherin, MMP2, MMP3, and MMP9, respectively. Data showed that, compared with the control group, the expression of all of these molecules increased unmistakably in the p-NDUFA4 transfection group (Figure 5G; $p < 0.05$). Finally, we also analyzed the possible change on the transduction of the Akt and Erk pathway. Western blotting assay showed that the expression level of NDUFA4 protein significantly increased in the p-NDUFA4 transfection group (Figure 5H; $p < 0.05$). Importantly, we noticed that the expression of phosphor-Akt and phosphor-Erk also increased unmistakably (Figure 5H; $p < 0.05$). Combining these data indicated that NDUFA4, as an oncogene, could promote growth and metastasis of lung cancer cells by regulating the Akt/Erk signaling pathway.

Overexpression of NDUFA4 Abrogated the Suppressive Effect of TTF-1-Promoter-Operating miR-7 Expression

Then, to further explore whether downregulation of NDUFA4 contributed to the suppressive effect of TTF-1-promoter-operating miR-7 expression on human lung cancer cells, we transiently co-transfected p-T-miR-7 and p-NDUFA4 into lung cancer cell line 95D cells and observed the possible change on cell growth and metastasis. As shown in Figures 6A and 6B, the proliferation of 95D cells decreased significantly in the p-T-miR-7 transfection group, which was consistent with our above data. Notably, we found that the proliferation of cells in the p-T-miR-7 and p-NDUFA4 co-transfection group elevated unmistakably ($p < 0.05$). Moreover, clone-formation assay showed that clone-formation ability of cells also increased significantly (Figure 6D; $p < 0.05$). Further analysis showed that the migration ability of cells in the p-T-miR-7 and p-NDUFA4 co-transfection group was enhanced compared with that in the p-T-miR-7 transfection group (Figure 6C; $p < 0.05$). Consistently, real-time PCR assay showed that the expression of NDUFA4, cell-growth-related molecules including CDK2, CDK3, CDK4, and CDK6, as well as metastasis-related molecules including CXCR4, E-Cadherin, MMP2, MMP3, and MMP9 were elevated significantly (Figures 6E and 6F; $p < 0.05$). Finally, we also analyzed the expression of phosphor-Akt and phosphor-Erk in the p-T-miR-7 and p-NDUFA4 co-transfection groups. Data showed that the expression of NDUFA4 protein increased unmistakably (Figure 6G; $p < 0.05$). Importantly, the level of both phosphor-Akt and phosphor-Erk significantly increased (Figure 6G; $p < 0.05$). Combining these results demonstrated that TTF-1-promoter-operating miR-7 expression affected the growth and metastasis of human lung cancer cells through NDUFA4.

The Change on Organs and Tissues in Nude Mice Model of Human Lung Cancer

Previous data showed that the biological change on organs and tissues were important for the relative safety of targeted gene therapy against cancer, which was critical for its potential application.[37,38] Then, we observed the possible change on various important organs and tissues in nude mice model of human lung cancer. As shown in Figure 7A, there were no significant changes in the morphology of six important organs and tissues, including heart, liver, spleen, kidney, brain, and lymph nodes, between the p-T-miR-7 injection group and the control group ($p > 0.05$). Moreover, we also did not find any difference in histology or weight in these organs and tissues (Figure 7B; $p > 0.05$). To confirm these findings, we further collected and detected the concentration of serum AST and ALT, which were critical indicators for the functional change of organs including heart and liver. It was noticed that none of these biochemistry indicators changed significantly (Figure 7C; $p > 0.05$), indicating that there were not any changes on biological function of important organs and tissues in the p-T-miR-7 injection group.

Finally, we preliminarily estimated the distribution of plasmid p-T-miR-7 in vivo. As shown in Figure S2, the copies of the p-T-miR-7 plasmid was higher in lung tissue and tumor tissue ($p < 0.05$), but

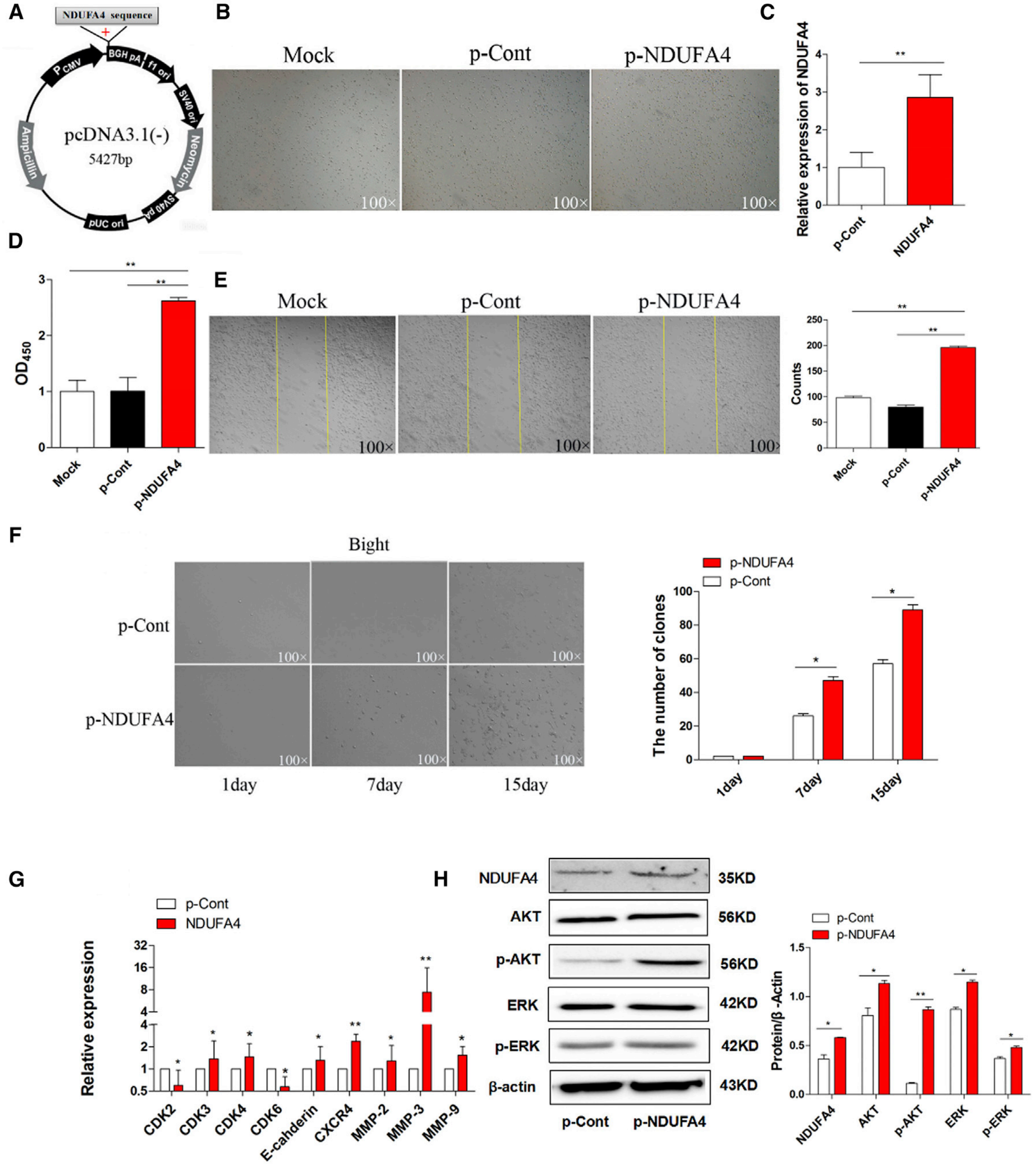

Figure 5. Overexpression of NDUFA4 Promoted the Proliferation and Migration of Human Lung Cancer Cells

(A) The sketch map of an eukaryotic expression vector encoding NDUFA4 (termed as p-NDUFA4). (B) Human lung cancer cell line 95D cells were transiently transfected with p-NDUFA4 (10 μg) or p-Cont (10 μg) in vitro. 48 hr later, the morphology of cells were observed by microscopy (magnification 100×). (C) The relative expression of NDUFA4 was determined by real-time PCR. (D) The proliferation of cells was detected by CCK-8 assay. (E) The migration of 95D cells was performed by scratch assay, and the cell number was also calculated. The yellow line indicates the border of scratch wound (magnification 100×). (F) The colon-formation assay also was performed and calculated at indicated time points. Human lung cancer cell line 95D cells were transiently transfected with p-NDUFA4 (10 μg) or p-Cont (10 μg) in vitro. 48 hr later, the expression of indicated cell-growth-related molecules and metastasis-related molecules was detected by real-time PCR assay (G). (H) The protein level of NDUFA4, Akt, p-Akt, Erk, and p-Erk was also detected by western blotting and calculated, respectively. Representative data of three independent experiments are shown.$^*p < 0.05$, $^{**}p < 0.01$.

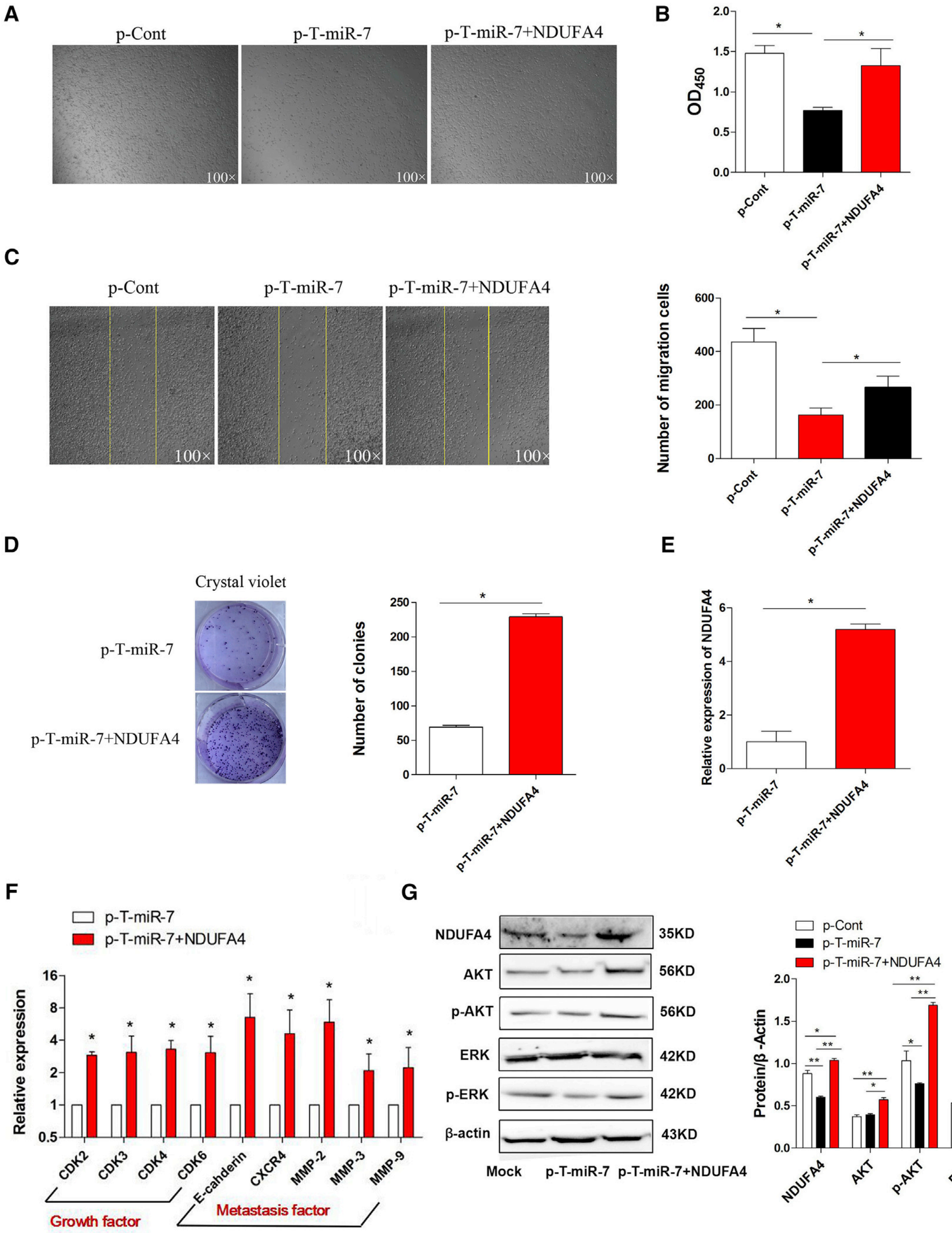

Figure 6. Overexpression of NDUFA4 Abrogated the Suppressive Effect of TTF-1-Promoter-Operating miR-7 Expression

Human lung cancer cell line 95D cells were transiently co-transfected with p-T-miR-7 (10 μg) and p-NDUFA4 (10 μg) in vitro. 48 hr later, (A) the morphology of cells were observed by microscopy (magnification 100×). (B) The proliferation of 95D cells was detected by CCK-8 assay. (C) The migration of 95D cells was performed by scratch assay, and the cell number was also calculated. The yellow line indicates the border of scratch wound (magnification 100×). (D) The colony-formation assay was also performed, and the colon numbers were calculated. (E) The relative expression of NDUFA4 was determined by real-time PCR. (F) The relative expression of indicated cell-growth-related molecules and metastasis-related molecules was also detected by real-time PCR assay. (G) The protein level of NDUFA4, Akt, p-Akt, Erk, and p-Erk was detected by western blotting and calculated, respectively. Representative data of three independent experiments are shown.*$p < 0.05$, **$p < 0.01$.

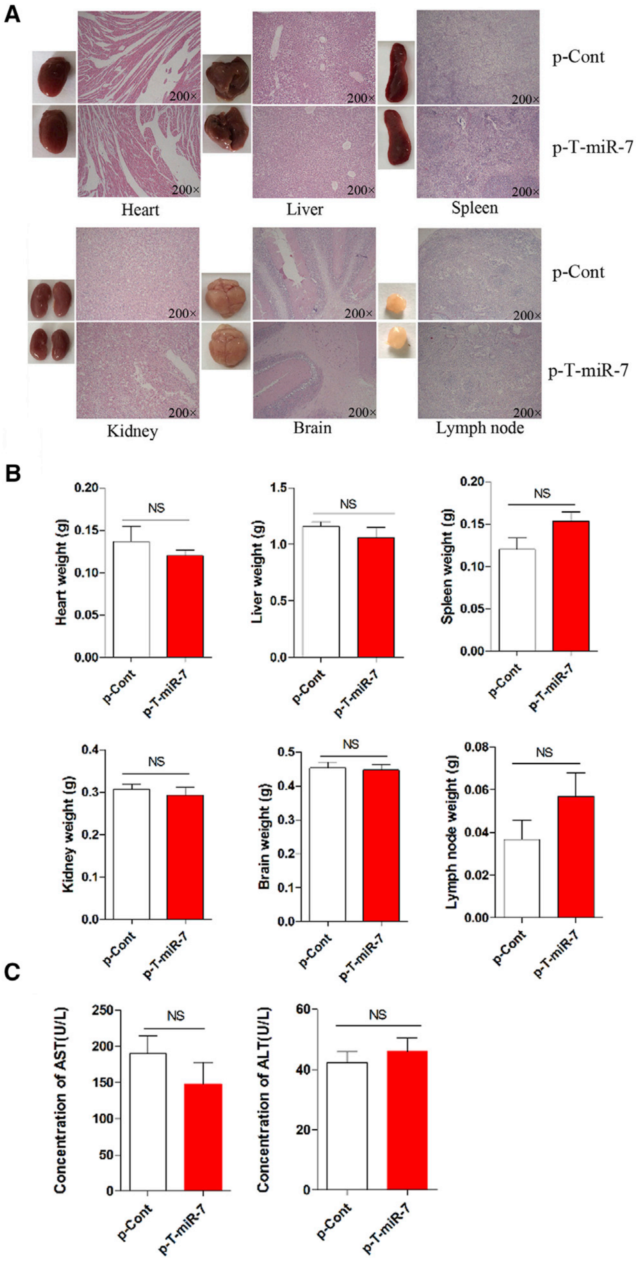

Figure 7. The Change on Important Organs and Tissues in Nude Mice Model of Human Lung Cancer

Human lung cancer cell line 95D cells were injected subcutaneously into right flank of BALB/c nude mice (n = 8). 7 days later, the plasmid of p-T-miR-7 (100 mg) or p-Cont (100 mg) was remotely given by subcutaneous injection into the left flank of nude mice five times every three days. 3 days after last injection, all of the mice were sacrificed. (A) The morphology and H&E staining of various organs and tissues, including heart, liver, spleen, kidney, brain and lymph nodes, were performed. (B) The weight of indicated organs and tissues also were obtained. (C) The concentration of the serum AST and ALT were measured, respectively. NS, no significance.

not in the other organs and tissues, indicating the plasmid was mainly distributed in the lung tissue and tumor tissue in vivo. However, when we further analyzed the expression level of miR-7, it was found that, compared with their corresponding control, miR-7 level increased significantly in heart, spleen, lung, intestine, and lymph nodes, respectively (Figure S3), indicating that intrinsic transcriptional factors in these organs and tissues might bind to TTF-1 promoter and manipulate the expression of miR-7.

DISCUSSION

It is the first time the potential value of the TTF-1 promoter operating distinct miRNAs expression in targeted gene therapy against lung cancer has been explored. We first observed that TTF-1 promoter could effectively operate miR-7 expression in lung cancer cells. Importantly, we found that TTF-1-promoter-operating miR-7 expression could effectively inhibit the growth of lung cancer cells in vitro and in vivo. Notably, we further revealed that the downregulation of NDUFA4, a novel target of miR-7, contributed to the effects of miR-7 expression operated by TTF-1 promoter on the growth and metastasis of human lung cancer cells, accompanied by altered transduction of related signaling pathway including the Akt and Erk pathway. Finally, we observed that there were not any significantly change in various important organs or tissues, indicating the safety value of strategy of TTF-1-promoter-operating expression of miR-7 in vivo.

It is well known that miR-7, as an intrinsic tumor suppressor, has been found to be an important regulator in the development of various cancers including lung cancer.[13,39–41] For example, Xiong et al.[42] documented that miR-7 could inhibit the growth of human lung cancer cells in vivo. And our recent work reported that the site mutation of miR-7 promoter region contributed to its altered expression in clinical lung cancer tissue.[18] Interestingly, some research works showed that in situ local injection of miR-7 overexpression plasmids could regulate the growth and metastatic potential of human lung cancer cells in vivo via the Akt pathway.[43,44] In the present study, we further extended previous findings to report that expression of miR-7, operated by TTF-1 promoter, could reduce the growth and metastasis of human lung cancer cells in vitro. Importantly, remote hypodermic injection, but not local injection, of plasmid p-T-miR-7 could significantly inhibit the growth and metastasis of human lung cancer cells in vivo, accompanied by altered expression of growth- and metastasis-associated molecules such as CDK and MMP family members. Therefore, combining these research works may highlight the fact that miR-7 is a critical regulator and might be an ideal target for gene therapy against clinical lung cancer, which would be helpful for the outcome of clinical treatment.

The technique of targeted gene expression, including distinct delivery systems and specific gene-promoter-operating expression, is an important strategy for gene therapy against various cancers including lung cancer.[4,7,45,46] Up to now, extensive literatures documented the efficacy of distinct delivery systems, such as the liposome system, nano-partical system, and polyetherimide (PEI) system, and so on,

in cancer gene therapy.[6,47,48] However, related documents on the potential value of targeted expression of interesting genes using specific gene promoter, such as tumor suppressor, in cancer gene therapy is still scare. The main obstacle might be the selection of ideal promoter in cancers. The TTF-1 gene is limitedly expressed in lung tissue and thyroid tissue. Interestingly, TTF-1 gene was a well-known lineage-specific oncogene in lung cancer, which dominantly expressed in lung cancer but not in other types of cancers.[20–22] Therefore, in the present study, we attempted to design and construct an eukaryotic vector encoding miR-7, which was manipulated by the TTF-1 promoter. Luckily, we found that this plasmid could effectively express miR-7 in human lung cancer cells with a higher expression level of TTF-1 in vitro, but not other human cancer cells with a lower expression level of TTF-1, including colon cancer, hepatocellular carcinoma, and gastric cancer, indicating that the TTF-1 promoter could effectively orchestrate targeted expression of miR-7 in lung cancer cells. Importantly, we further found that TTF-1-promoter-operating miR-7 expression could significantly not only inhibit the growth and metastasis of human lung cancer cells in vivo, but also induce the apoptosis of cancer cells in vivo. These data suggested that TTF-1 promoter might be an ideal candidate operator for targeted expression of interesting genes in lung cancer gene therapy. Therefore, successive research work on both the potential effect of TTF-1-promoter-operating miR-7 expression on other types of cancers and regulatory factors including transcript factors in the activation of TTF-1 promoter, which did not been investigated in present study, is valuable for the verification of usage of the TTF-1 promoter in targeted gene expression in lung cancer and can ultimately benefit the development of a therapeutic strategy in clinical lung cancer.

Accumulating evidence showed that the molecular mechanism through which miR-7 regulated the growth and metastasis of lung cancer cells was complex, and various genes including PA28 gamma, KLF4, BCL-2, and so on also were reportedly involved in the biological function of miR-7 in lung cancer.[15,49,50] To reach a comprehensive knowledge on the molecular mechanism of the effect of TTF-1-operating miR-7 expression on the growth and metastasis of lung cancer cells, we used a global gene expression chip technique combined with biological information analysis to screen the potential target of miR-7. Unexpectedly, we found that TTF-1-operating miR-7 expression could significantly reduce the expression level of NDUFA4 in lung cancer cells in vitro and in vivo. Importantly, overexpression of NDUFA4 could abrogate the effect of TTF-1-operating miR-7 expression on the growth and metastasis of lung cancer cells, accompanied by altered transduction of the Akt and Erk pathway, which was critical for the growth and metastatic potential of lung cancer cells. These data demonstrated that NUDFA4, a novel target of miR-7, contributed to the effects of TTF-1-operating miR-7 expression in lung cancer cells. In particular, we also found that overexpression of NUDFA4 could enhance the proliferation and metastatic potential of human lung cancer cells and elevate the transduction of the Akt and Erk pathway. In accordance with these findings, recent literatures reported that the expression level of NDUFA4 was closely associated with the progression and/or worse prognosis of various types of cancers.[6,35] For example, Franziska et al.[36] found that downregulated expression of NDUFA4 in clear cell renal cell carcinoma was related to cancer-specific survival. These research works suggested that NDUFA4 may be as an oncogene in the development of cancers including lung cancer. However, the exact role of NDUFA4 in the development of lung cancer, such as the value of NDUFA4 expression in prognosis of lung cancer patients, still remains to be fully elucidated in successive research work.

It is well known that the safety of gene therapy strategy, including functional change of important organs and distribution of exogenous DNA, was critical for potential application of cancer gene therapy.[37,38,51] In the present study, we monitored the possible change on several important organs including liver, heart, spleen, and so on and found that there were not any difference on morphology or histology of these important organs and tissues. Moreover, we also found that there were not significant changes in serum level of aspartate transaminase (AST) and alanine aminotransferase (ALT), which were representative reflectors in the functional change of organs including heart and liver. Regarding distribution of plasmid, Thanaketpaisarn et al.[52] reported that naked plasmid DNA (pDNA) encoding firefly luciferase was directly injected into the tail vein of mice and found that the plasmid was mostly enriched in liver, spleen, and kidney. Mahato et al.[53] further investigated the disposition characteristics of pDNA complexed with cationic liposomes after intravenous injection in mice and found that liposomal pDNA encoding gene expression was enriched in lung, heart, kidney, and spleen, but not in liver. Some literature documented naked pDNA enriched in the liver in vivo after hydrodynamic injection via tail vein.[54] Different from these research works, we analyzed the distribution of plasmid p-T-miR-7 after remote hypodermic injection and found that the plasmid was dominantly enriched in lung tissue and tumor mass in vivo. To the diverse phenomenon, we proposed two factors may be closely related to the different distribution of pDNA in vivo. The first factor was the different experimental settings including entry route and way, as well as the dosage, of naked pDNA. The second factor was the different hemodynamics and perfusion in distinct organs and tissues. Thereby, further studies on the dynamic distribution of the plasmid and the possible change on other organs are important for the evaluation of safety of the plasmid in vivo, which is critical for the potential application of gene therapy based on targeted miR-7 expression in lung cancer.

In conclusion, for the first time, our study demonstrated that TTF-1-promoter-operating miR-7 expression could significantly inhibit the growth of human lung cancer in vitro and in vivo, closely related to downregulation of NDUFA4 and altered transduction of related signaling pathways including Akt and Erk. These data indicated that TTF-1-promoter-operating miR-7 expression might be an ideal strategy in lung cancer, which provided preliminary experimental basis for targeted expression of distinct miRNA in lung cancer and was helpful for the development of gene therapy against clinical lung cancer.

MATERIALS AND METHODS

Construction of Eukaryotic Vector

The genes for the miR-7 (NM-407044) were expanded by PCR from human DNA derived from 95D cells using a forward primer (5′-CGACGCGTAAGAGAGAAATGAGCCACTTGC) and a reverse primer (5′-CCCAAGCTTCCTGCCACAGTGGGGGATG) and then subcloned into *MulI* and HindIII sites of pGL3.0 basic vector (Invitrogen) to generate pGL3.0-basic-miR-7 vector (termed as p-miR-7). Afterward, for the construction of the PGL3-basic-TTF-1-promoter-miR-7 (termed as p-T-miR-7) vector, the promoter region of TTF-1 (NM-7080) was amplified from DNA derived from 95D cells using a forward primer (5′-CGGGGTACCTGTTTCGGCAACTAC) and a reverse primer (5-CGACGCGTCCTTCTGGGTCCTT) and subcloned into *KpnI* and *MulI* sites of p-miR-7 vector. The gene for the NDUFA4(NM-4697) were expanded by PCR from human cDNA derived from 95D cells using a forward primer (5′-GCTCTAGAGGCTAGGTCGGTTCTCTCCT) and a reverse primer (5′-CGGGATCCGTGGAAAATTGTGCGGATGT) and then subcloned into *XbaI* and *BamHI* sites of pcDNA3.1 vector (Invitrogen) to generate pcDNA3.1-NDUFA4 vector (termed as p-NDUFA4). Clone identity was verified using restriction digest analysis and plasmid DNA sequencing. Endotoxin-free plasmids were obtained using Endofree plasmid mega kit (QIAGEN). Then, plasmids were transiently transferred into the 95D cells using Lipofectamine-2000 (Invitrogen) in different following experiments according to the manufacturer's instructions.

Cell Culture and Transfection

Human lung cancer cell line 95D cells, A549 cells, NCI-H292 cells, gastric cancer cell line SGC901 cells, hepatic cancer cell line HepG2 cells, as well as colon cancer cell line SW620 cells were obtained from National Rodent Laboratory Animal Resource. Colon cancer cell line SW620 cells were cultured in McCoy 5A, RPMI-1640 containing 100 IU/mL penicillin, 100 μg/mL streptomycin, 20 mM glutamine, and 10% heat-inactivated fetal bovine serum (FBS). All of other cells were cultured in RPMI-1640 containing 100 IU/mL penicillin, 100 μg/mL streptomycin, 20 mM glutamine and 10% heat-inactivated fetal bovine serum (FBS). All cells were cultured in a humidified atmosphere of 5% CO_2 at 37°C. For transfection, cells were seeded at 70%–80% confluence, and 12 hr later cells were transiently transfected with indicated vectors with Lipofectamine 2000 according to the manufacturer's instruction. Cells were harvested at indicated time point in following experiments.

Real-Time PCR Assay

The conventional primers were obtained from Shanghai Sangon Biological Engineering, the TaqMan probes of miR-7 (000386) and U6 (001793) were purchased from Life Technologies, and the other reagents were from TAKARA Bio. RT-PCR and real-time PCR were performed according to the manufacturer's protocols. The following primers were used: CDK6: forward: 5′-AAGCCTCTTTTTCGTGGAAGT-3′, reverse: 5′-GGTTGGGCAGATTTTGA-ATG-3′; CDK4, forward: 5′-ATTGGTGTCGGTGCCTATG-3′, reverse: 5′-AACTGTGCTGATGG-GAAGG-3′; CDK3, forward: 5′-GCTCTTTCGTATCTTTCGTATGC-3′, reverse: 5′-ATTGGTGTCGGTGCCTATG-3′; CDK2, forward: 5′-TTTGCTGAGATGGTGACTCG-3′, reverse: 5′-TGGGGA-AACTTGGCTTGTAA-3′; E-cadherin, forward: 5′-TGATTCTGCTGCTCTTGCTG-3′, reverse: 5′-CTCTTCTCCGCCTCCTTCTT-3′; CXCR4, forward: 5′-TGACCGCTTCTACCCCAAT-3′, reverse: 5′-AGCCAGGATGAGGATGACTG-3′; MMP9, forward: 5′-TCTTCCCCTTCACTTTCCTG-3′, reverse: 5′-CCCACTTCTTGTCGCTGTC-3′; MMP3, forward: 5′-ATCCCGAAGTGGAGGAAAAC3′, reverse: 5′-AGCCTGGAGAATGTGAGTGG-3′; MMP2, forward: 5′-TATGGCTTCTGCCCT-GAGAC-3′, reverse: 5′-CACACCACATCTTTCCGTCA-3′; TPS2, forward: 5′-CAAAAGTGGGA-AACCAGCAT-3′, reverse: 5′-GATGAGCAGGCGGTAATAGG-3′; TRMT13, forward: 5′-TGTCCCATCCAGCATTACAC-3′, reverse: 5′-GCTCCAAACTCAACAAAGCA-3′; SAYSD1, forward: 5′GCAGCACATCAGAGACACCA-3′, reverse: 5′-GCAGGACCAACCAGAGAAGA-3′.LRRC8B, forward: 5′-GTGGTGGATGCTGAGGAGTT-3′, reverse: 5′-AGCCAGATGAAGGATGAAGG-3′; CNN3, forward: 5′-AATGAGTGTGTATGGGCTTGG-3′, reverse: 5′-TGTTCCTGTTCCTTGGCTTC-3′; CHAMP1, forward: 5′-ATGAAGCGTGGAAAAGGAAA-3′, reverse: 5′-GCATTTGTAAG-GGCTATGAACA-3′; TMEM97, forward: 5′-TGCCCCCTACTTACTCATCC-3′, reverse: 5′-CAA-CAAGCAACCACCCTGTA-3′; NDUFA4, forward: 5′-TCCCCCTCTTTGTATTTATTGG-3′, reverse: 5′-GGGCTCTGGGTTATTTCTGTC-3′.PIGH, forward: 5′-CCAGAAAGCCACATCAACAA-3′, reverse: 5′-TACGGAAAACCAGCCCCTAT-3′; C5orf22, forward: 5′-GGCACCAACCTA–ACAGAGGA-3′, reverse: 5′-CCGTTTCTTCATCATCACCA-3′; NXT2, forward: 5′-ACTGCTAC-AAGGTCCCAGATG-3′, reverse: 5′-TGGTTAGTGCCCGTCTTCTT-3′. Gene expression levels were quantified using the BIO-RAD CFX96 detection system (Bio-Rad). Relative expression of these indicated genes was calculated using the comparative threshold cycle (Ct) method.

Cell Counting kit-8 Assay

95D cells were seeded in 96-well plates at 1×10^4/well with triplicate and transiently transfected with p-T-miR-7 plasmid (2 μg), p-NDUFA4 plasmid (10 μg), or p-Cont plasmid (2 μg/10 μg). At indicated time points, cells were detected using cell-counting kit-8 (CCK-8) assay. In brief, 20 μL CCK-8 solution was added into each well. After 3 hr of incubation at 37°C, the absorbance was measured with a spectrophotometer at 450 nm with 600 nm as a reference.

Colony-Formation Assay

95D cells were transiently transfected with p-T-miR-7 plasmid (2 μg), p-NDUFA4 plasmid (10 μg), or p-Cont plasmid (2 μg/10 μg), as described above. 24 hr later, cells were trypsinized to single-cell suspension and seeded in 6-well plates at 200/well and 800/well for the clone-forming experiment. Then, cells were incubated in a humidified atmosphere of 5% CO_2 at 37°C. 15 days later, the colonies were stained with crystal violet, and the colony diameter and number were statistically analyzed.

Animal Experiment

All animals were housed in the pathogen-free mouse colony at our institution, all animal experiments were performed according to the Guidelines for the Care and Use of Laboratory Animals (Ministry

of Health, People's Republic of China, 1998), and all the experimental procedures were approved by the ethical guidelines of Shanghai Medical Laboratory Animal Care and Use Committee (permit number: 2013018). Female nude mice (BALB/C, 4−6 weeks old) were purchased from Shanghai Laboratory Animal Center. For preparation of subcutaneous xenograft model, 0.2 mL lung cancer cell line 95D cells (7.0×10^6) was injected subcutaneously into the right flank of nude mice. 7 days after tumor cell inoculation with confirmation of successful maturation of tumors, 16 mice were divided randomly into two groups (eight mice per group). The plasmid p-T-miR-7 or p-Cont (100 mg) was given locally by direct injection into the left flank of nude mice five times every 3 days. Meanwhile, tumor volumes were determined (in cubic millimeter) as previous description.[55] After 18 days of treatment, all mice were sacrificed. Tumor tissue and other organs were excised and divided into two parts. One part was stored in −80°C for further use. And another section was fixed in formalin and embedded in paraffin used for H&E staining.

In Situ Hybridization

To evaluate the cellular distribution of miR-7 in the tumors, in situ hybridization was performed according to our previous description[56] with some modifications. Briefly, before hybridization incubation, all solutions were prepared with diethyl pyrocarbonate-treated water. After deparaffinization and rehydration, tissue sections were treated by proteinase K digestion. After blocking with normal goat serum (1:100), sections were next incubated or microwave heating and then incubated with hybridization cocktail containing miR-7 probe (1:1,000 dilution; EXIQON; no. 38485-01) at 42°C for 16 hr. Then, alkaline phosphatase-labeled anti-digoxigenin antibody (1:500) (Roche Diagnostics) and the reaction products were colorized with nitro blue tetrazolium/5-bromo-4-choloro-3-indolyl phosphate (NBT/BCIP) (ZSGB-Bio). Then, the tissues were counterstained with Mayer's hematoxylin and systematically viewed under a light microscope (Olympus IX-71).

Histopathology

Tumor tissue and other organs were fixed in 4% paraformaldehyde and embedded in paraffin and then cut into 5-μm-thick sections. Sections were stained with H&E, and images were taken with an Olympus IX71 microscope. All tumor tissues and other organs fields at original magnification 10×, 20×, and 40× were examined for each sample.

Immunofluorescence: Ki-67 Staining and TUNEL Assay

To assess tumor cell proliferation and apoptosis in situ after the plasmid of p-T-miR-7 injection, immunofluorescence staining using anti-proliferation cell nuclear antigen (Ki-67) antibody and TUNEL (terminal deoxynucleotidyl transferase dUTP nick end labeling) assay was performed on all frozen sections of tumor tissues. After being deparaffinized and rehydrated, slides were incubated in citrate buffer (pH 6.0), and then antigen retrieval was performed in a microwave two times for 5 min. After cooling down at room temperature, slides were washed twice for 5 min with PBS and incubated with 10% normal goat serum for 30 min at room temperature. First, to determine tumor cell proliferation, anti-Ki-67 antibody (sc-7907, 1/100, rabbit-anti-mouse, Santa Cruz Biotechnology) in PBS were added, and the slides were incubated at 4°C overnight. After three washes in PBS for 5 min, secondary antibody Alexa-Fluor-488-conjugated goat-anti-rabbit immunoglobulin G (IgG) (1/250, Invitrogen) was added and allowed to incubate for 1 hr in the dark at room temperature. After three washes in PBS for 5 min, the slides were counterstained, mounted with SlowFade Gold antifade reagent with DAPI (Invitrogen), and left for 10 min in the dark at room temperature before examination by fluorescence microscopy (Zeiss Axioplan 2). In addition, for testing tumor cell apoptosis in situ, a TUNEL assay was performed according to the manufacturer's protocol. In brief, after antigen retrieval, the slides were washed in PBS three times and incubated with 50 μL of TUNEL reaction mixture (11684817910, In situ Cell Death Detection Kit, Roche) for 2 hr at 37°C. At last, after three washes in PBS for 5 min, the slides were also counterstained by DAPI (Invitrogen) and evaluated by fluorescence microscopy.

Wound-Healing Assays

Human lung cancer 95D cells were transiently transfected with p-T-miR-7 plasmid (2 μg), p-NDUFA4 plasmid (10 μg), or p-Cont plasmid (2 μg/10 μg) or scramble control as described above. Cells were then seeded in 6-well plates at 1×10^5 per well in growth medium. Confluent monolayers were starved overnight in assay medium, and a single scratch wound was created using a micropipette tip. The cells were washed with PBS to remove cell debris. Images were captured with a microscope at 48 hr postwounding. For a quantitative measure, we counted the cells migrating to the wound area based on the image at 0 hr postwounding.

Western Blotting

Western blotting was performed on cytosolic cellular extracts. Equal amounts of protein were resolved under reducing conditions on a 10% SDS-PAGE gel. Protein migration was assessed using protein standards (Bio-Rad). Transfer to a nitrocellulose membrane was performed 60 min at 250 mA using a wet transfer system. Equal protein loading was confirmed with Ponceau staining. The membrane was washed in 5% skim milk in PBS plus 0.05% Tween 20 (PBST) for 2 hr to block nonspecific protein-binding sites on the membrane. Immunoblotting was performed using rabbit polyclonal anti-human NDUFA4 antibody (Santa Cruz Biotechnology; no. Sc-517091), rabbit monoclonal anti-human ERK antibody (Cell Signaling Technology; no. 4695), rabbit polyclonal anti-human p-ERK antibody (Cell Signaling Technology; no. 4370), rabbit monoclonal anti-human AKT antibody (Cell Signaling Technology; no. 4691), rabbit monoclonal anti-human p-AKT antibody (Cell Signaling Technology; no. 4060), and rabbit-monoclonal anti-human GAPDH antibody (Cell Signaling Technology; no. 2118) or rabbit monoclonal rabbit monoclonal anti-human β-actin antibody, respectively (Cell Signaling Technology; no. 4970) at a dilution of 1/1,000 in a non-fat milk-Tris buffer. The membrane was then washed in PBST and subsequently probed with a secondary anti-rabbit antibody (Ab)-conjugated to horseradish peroxidase (HRP) (Cell Signaling Technology; no. 7074) at a dilution of 1:2,000. The signal was detected and analyzed

using the chemiluminescence Imaging System (ChemiScope5600, CLINX); each experiment was performed in triplicate.

Gene Expression Array

Total RNA was first converted to cDNA, followed by in vitro transcription to make cRNA. 5 μg of single-stranded cDNA was synthesized, end labeled, and hybridized, for 16 hr at 45°C, to Human Gene 1.0 ST arrays. All washing steps were performed by a GeneChip Fluidics Station 450 and GeneChip were scanned with the Axon GenePix 4000B microarray scanner. Partek was used to determine ANOVA p values and fold changes for genes.

Immunohistochemistry

Immunohistochemical staining was performed following standard procedures. The formalin-fixed paraffin-embedded tissues were sliced into 5-μm-thick sections, deparaffinized, and rehydrated. Then, the cells were fixed with 95% ethanol for 15 min. Antigen retrieval was performed in 10 mmol/L citric acid buffer (pH 6.0) for 10 min using a 750-W microwave. Endogenous peroxidase activity was blocked with 3% hydrogen peroxide in methanol for 15 min. After incubation with rabbit anti-human NDUFA4 antibody (1:1,000 dilution; Santa Cruz Biotechnology; no. Sc-517091), the sections were washed in PBA and incubated with a polymer horseradish peroxidase-conjugated secondary antibody (ZSGB-Bio) for 60 min. The sections were further incubated with Liquid DAB Large-Volume Substrate-Chromogen System (ZSGB-Bio) and counterstained with hematoxylin. The immunostaining was evaluated using an Olympus IX-71 light microscope (Olympus).

Statistical Analyses

The data were analyzed with GraphPad Prism 5.0 and were presented as the mean ± SD. Student's t test was used when two conditions were compared, and ANOVA with Bonferroni or Newman-Keuls correction was used for multiple comparisons. $p < 0.05$ was considered significant; two-sided tests were performed.

SUPPLEMENTAL INFORMATION

Supplemental Information includes Supplemental Materials and Methods, three figures, and one table and can be found with this article online at http://dx.doi.org/10.1016/j.omtn.2016.12.005.

AUTHOR CONTRIBUTIONS

L.L. and C.C. performed the experiments, analyzed the data, and wrote the paper; J.Z. performed the experiments and analyzed the data; H.W. and M.G. performed the experiments; Y.Z., J.L., and J.Z. wrote the paper; L.X. conceived and designed the experiments, analyzed the data, and wrote the paper, and all authors reviewed the paper.

CONFLICTS OF INTEREST

All authors declare that the research was conducted in the absence of any commercial or financial relationships that could be construed as a potential conflict of interest.

ACKNOWLEDGMENTS

This work was supported by Program for High level innovative talents in Guizhou Province (QKH-RC-2016-4031), National Natural Science Foundation of China (31370918), Program for New Century Excellent Talents in University, Ministry of Education of China (NCET-12-0661), Program for Excellent Young Talents of Zunyi Medical University (15ZY-001), and Project of Guizhou Provincial Department of Science and Technology (2009C491).

REFERENCES

1. Sun, C.C., Li, S.J., Zhang, F., Zhang, Y.D., Zuo, Z.Y., Xi, Y.Y., Wang, L., and Li, D.J. (2016). The novel miR-9600 suppresses tumor progression and promotes paclitaxel sensitivity in non-small-cell lung cancer through altering STAT3 expression. Mol. Ther. Nucleic Acids *5*, e387.
2. Papadimitrakopoulou, V., Lee, J.J., Wistuba, I.I., Tsao, A.S., Fossella, F.V., Kalhor, N., Gupta, S., Byers, L.A., Izzo, J.G., Gettinger, S.N., et al. (2016). The BATTLE-2 study: A biomarker-integrated targeted therapy study in previously treated patients with advanced non-small-cell lung cancer. J. Clin. Oncol., Published online August 1, 2016. http://dx.doi.org/10.1200/JCO.2015.66.0084.
3. Espana-Serrano, L., and Chougule, M.B. (2016). Enhanced anticancer activity of PF-04691502, a dual PI3K/mTOR inhibitor, in combination with VEGF siRNA against non-small-cell lung cancer. Mol. Ther. Nucleic Acids *5*, e384.
4. Xue, H.Y., Guo, P., Wen, W.C., and Wong, H.L. (2015). Lipid-based nanocarriers for RNA delivery. Curr. Pharm. Des. *21*, 3140–3147.
5. Lee, H.Y., Mohammed, K.A., and Nasreen, N. (2016). Nanoparticle-based targeted gene therapy for lung cancer. Am. J. Cancer Res. *6*, 1118–1134.
6. Salzano, G., Costa, D.F., Sarisozen, C., Luther, E., Mattheolabakis, G., Dhargalkar, P.P., and Torchilin, V.P. (2016). Mixed nanosized polymeric micelles as promoter of doxorubicin and miRNA-34a co-delivery triggered by dual stimuli in tumor tissue. Small *12*, 4837–4848.
7. Amodio, N., Stamato, M.A., Gullà, A.M., Morelli, E., Romeo, E., Raimondi, L., Pitari, M.R., Ferrandino, I., Misso, G., Caraglia, M., et al. (2016). Therapeutic targeting of miR-29b/HDAC4 epigenetic loop in multiple myeloma. Mol. Cancer Ther. *15*, 1364–1375.
8. Peng, Y.F., Shi, Y.H., Ding, Z.B., Zhou, J., Qiu, S.J., Hui, B., Gu, C.Y., Yang, H., Liu, W.R., and Fan, J. (2013). α-Fetoprotein promoter-driven Cre/LoxP-switched RNA interference for hepatocellular carcinoma tissue-specific target therapy. PLoS ONE *8*, e53072.
9. Iaboni, M., Russo, V., Fontanella, R., Roscigno, G., Fiore, D., Donnarumma, E., Esposito, C.L., Quintavalle, C., Giangrande, P.H., de Franciscis, V., and Condorelli, G. (2016). Aptamer-miRNA-212 conjugate sensitizes NSCLC cells to TRAIL. Mol. Ther. Nucleic Acids *5*, e289.
10. Lu, Y.J., Liu, R.Y., Hu, K., and Wang, Y. (2016). MiR-541-3p reverses cancer progression by directly targeting TGIF2 in non-small cell lung cancer. Tumour Biol. *37*, 12685–12695.
11. Chan, L.W., Wang, F.F., and Cho, W.C. (2012). Genomic sequence analysis of EGFR regulation by microRNAs in lung cancer. Curr. Top. Med. Chem. *12*, 920–926.
12. Zhao, J.G., Men, W.F., and Tang, J. (2015). MicroRNA-7 enhances cytotoxicity induced by gefitinib in non-small cell lung cancer via inhibiting the EGFR and IGF1R signalling pathways. Contemp. Oncol. (Pozn.) *19*, 201–206.
13. Luo, J., Li, H., and Zhang, C. (2015). MicroRNA-7 inhibits the malignant phenotypes of non-small cell lung cancer in vitro by targeting Pax6. Mol. Med. Rep. *12*, 5443–5448.
14. Zeng, C.Y., Zhan, Y.S., Huang, J., and Chen, Y.X. (2016). MicroRNA-7 suppresses human colon cancer invasion and proliferation by targeting the expression of focal adhesion kinase. Mol. Med. Rep. *13*, 1297–1303.
15. Xiong, S., Zheng, Y., Jiang, P., Liu, R., Liu, X., Qian, J., Gu, J., Chang, L., Ge, D., and Chu, Y. (2014). PA28gamma emerges as a novel functional target of tumour suppressor microRNA-7 in non-small-cell lung cancer. Br. J. Cancer *110*, 353–362.

16. Li, J., Zheng, Y., Sun, G., and Xiong, S. (2014). Restoration of miR-7 expression suppresses the growth of Lewis lung cancer cells by modulating epidermal growth factor receptor signaling. Oncol. Rep. *32*, 2511–2516.

17. Xu, L., Wen, Z., Zhou, Y., Liu, Z., Li, Q., Fei, G., Luo, J., and Ren, T. (2013). MicroRNA-7-regulated TLR9 signaling-enhanced growth and metastatic potential of human lung cancer cells by altering the phosphoinositide-3-kinase, regulatory subunit 3/Akt pathway. Mol. Biol. Cell *24*, 42–55.

18. Zhao, J., Wang, K., Liao, Z., Li, Y., Yang, H., Chen, C., Zhou, Y.A., Tao, Y., Guo, M., Ren, T., and Xu, L. (2015). Promoter mutation of tumor suppressor microRNA-7 is associated with poor prognosis of lung cancer. Mol. Clin. Oncol. *3*, 1329–1336.

19. Zhao, J., Tao, Y., Zhou, Y., Qin, N., Chen, C., Tian, D., and Xu, L. (2015). MicroRNA-7: A promising new target in cancer therapy. Cancer Cell Int. *15*, 103.

20. Puglisi, F., Barbone, F., Damante, G., Bruckbauer, M., Di Lauro, V., Beltrami, C.A., and Di Loreto, C. (1999). Prognostic value of thyroid transcription factor-1 in primary, resected, non-small cell lung carcinoma. Mod. Pathol. *12*, 318–324.

21. Barletta, J.A., Perner, S., Iafrate, A.J., Yeap, B.Y., Weir, B.A., Johnson, L.A., Johnson, B.E., Meyerson, M., Rubin, M.A., Travis, W.D., et al. (2009). Clinical significance of TTF-1 protein expression and TTF-1 gene amplification in lung adenocarcinoma. J. Cell. Mol. Med. *13* (8B), 1977–1986.

22. Di Loreto, C., Di Lauro, V., Puglisi, F., Damante, G., Fabbro, D., and Beltrami, C.A. (1997). Immunocytochemical expression of tissue specific transcription factor-1 in lung carcinoma. J. Clin. Pathol. *50*, 30–32.

23. Zhao, Q., Xu, S., Liu, J., Li, Y., Fan, Y., Shi, T., Wei, S., Tang, S.C., Liu, H., and Chen, J. (2015). Thyroid transcription factor-1 expression is significantly associated with mutations in exon 21 of the epidermal growth factor receptor gene in Chinese patients with lung adenocarcinoma. Onco Targets Ther. *8*, 2469–2478.

24. Nagashio, R., Ueda, J., Ryuge, S., Nakashima, H., Jiang, S.X., Kobayashi, M., Yanagita, K., Katono, K., Satoh, Y., Masuda, N., et al. (2015). Diagnostic and prognostic significances of MUC5B and TTF-1 expressions in resected non-small cell lung cancer. Sci. Rep. *5*, 8649.

25. Civitareale, D., Lonigro, R., Sinclair, A.J., and Di Lauro, R. (1989). A thyroid-specific nuclear protein essential for tissue-specific expression of the thyroglobulin promoter. EMBO J. *8*, 2537–2542.

26. Cardnell, R.J., Behrens, C., Diao, L., Fan, Y., Tang, X., Tong, P., Minna, J.D., Mills, G.B., Heymach, J.V., Wistuba, I.I., et al. (2015). An integrated molecular analysis of lung adenocarcinomas identifies potential therapeutic targets among TTF1-negative tumors including DNA repair proteins and Nrf2. Clin. Cancer Res. *21*, 3480–3491.

27. Sun, X., Li, J., Sun, Y., Zhang, Y., Dong, L., Shen, C., Tu, Y., and Tao, J. (2016). miR-7 reverses the resistance to BRAFi in melanoma by targeting EGFR/IGF-1R/CRAF and inhibiting the MAPK and PI3K/AKT signaling pathways. Oncotarget *7*, 53558–53570.

28. Liu, Z., Jiang, Z., Huang, J., Huang, S., Li, Y., Yu, S., Yu, S., and Liu, X. (2014). miR-7 inhibits glioblastoma growth by simultaneously interfering with the PI3K/ATK and Raf/MEK/ERK pathways. Int. J. Oncol. *44*, 1571–1580.

29. Chou, Y.T., Lin, H.H., Lien, Y.C., Wang, Y.H., Hong, C.F., Kao, Y.R., Lin, S.C., Chang, Y.C., Lin, S.Y., Chen, S.J., et al. (2010). EGFR promotes lung tumorigenesis by activating miR-7 through a Ras/ERK/Myc pathway that targets the Ets2 transcriptional repressor ERF. Cancer Res. *70*, 8822–8831.

30. Li, Y.J., Wang, C.H., Zhou, Y., Liao, Z.Y., Zhu, S.F., Hu, Y., Chen, C., Luo, J.M., Wen, Z.K., and Xu, L. (2013). TLR9 signaling repressed tumor suppressor miR-7 expression through up-regulation of HuR in human lung cancer cells. Cancer Cell Int. *13*, 90.

31. Wilcox, C.B., Feddes, G.O., Willett-Brozick, J.E., Hsu, L.C., DeLoia, J.A., and Baysal, B.E. (2007). Coordinate up-regulation of TMEM97 and cholesterol biosynthesis genes in normal ovarian surface epithelial cells treated with progesterone: Implications for pathogenesis of ovarian cancer. BMC Cancer *7*, 223.

32. Qiu, G., Sun, W., Zou, Y., Cai, Z., Wang, P., Lin, X., Huang, J., Jiang, L., Ding, X., and Hu, G. (2015). RNA interference against TMEM97 inhibits cell proliferation, migration, and invasion in glioma cells. Tumour Biol. *36*, 8231–8238.

33. Vinatzer, U., Gollinger, M., Müllauer, L., Raderer, M., Chott, A., and Streubel, B. (2008). Mucosa-associated lymphoid tissue lymphoma: Novel translocations including rearrangements of ODZ2, JMJD2C, and CNN3. Clin. Cancer Res. *14*, 6426–6431.

34. Urzúa, U., Roby, K.F., Gangi, L.M., Cherry, J.M., Powell, J.I., and Munroe, D.J. (2006). Transcriptomic analysis of an in vitro murine model of ovarian carcinoma: Functional similarity to the human disease and identification of prospective tumoral markers and targets. J. Cell. Physiol. *206*, 594–602.

35. Balsa, E., Marco, R., Perales-Clemente, E., Szklarczyk, R., Calvo, E., Landázuri, M.O., and Enríquez, J.A. (2012). NDUFA4 is a subunit of complex IV of the mammalian electron transport chain. Cell Metab. *16*, 378–386.

36. Müller, F.E., Braun, M., Syring, I., Klümper, N., Schmidt, D., Perner, S., Hauser, S., Müller, S.C., and Ellinger, J. (2015). NDUFA4 expression in clear cell renal cell carcinoma is predictive for cancer-specific survival. Am. J. Cancer Res. *5*, 2816–2822.

37. Lesina, E., Dames, P., Flemmer, A., Hajek, K., Kirchner, T., Bittmann, I., and Rudolph, C. (2010). CpG-free plasmid DNA prevents deterioration of pulmonary function in mice. Eur. J. Pharm. Biopharm. *74*, 427–434.

38. Halpern, M.D., Kurlander, R.J., and Pisetsky, D.S. (1996). Bacterial DNA induces murine interferon-gamma production by stimulation of interleukin-12 and tumor necrosis factor-alpha. Cell. Immunol. *167*, 72–78.

39. Glover, A.R., Zhao, J.T., Gill, A.J., Weiss, J., Mugridge, N., Kim, E., Feeney, A.L., Ip, J.C., Reid, G., Clarke, S., et al. (2015). MicroRNA-7 as a tumor suppressor and novel therapeutic for adrenocortical carcinoma. Oncotarget *6*, 36675–36688.

40. Liu, H., Wu, X., Huang, J., Peng, J., and Guo, L. (2015). miR-7 modulates chemoresistance of small cell lung cancer by repressing MRP1/ABCC1. Int. J. Exp. Pathol. *96*, 240–247.

41. Fang, Y., Xue, J.L., Shen, Q., Chen, J., and Tian, L. (2012). miR-7 inhibits tumor growth and metastasis by targeting the PI3K/AKT pathway in hepatocellular carcinoma. Hepatology *55*, 1852–1862.

42. Xiong, S., Zheng, Y., Jiang, P., Liu, R., Liu, X., and Chu, Y. (2011). MicroRNA-7 inhibits the growth of human non-small cell lung cancer A549 cells through targeting BCL-2. Int. J. Biol. Sci. *7*, 805–814.

43. Wang, W., Dai, L.X., Zhang, S., Yang, Y., Yan, N., Fan, P., Dai, L., Tian, H.W., Cheng, L., Zhang, X.M., et al. (2013). Regulation of epidermal growth factor receptor signaling by plasmid-based microRNA-7 inhibits human malignant gliomas growth and metastasis in vivo. Neoplasma *60*, 274–283.

44. Rai, K., Takigawa, N., Ito, S., Kashihara, H., Ichihara, E., Yasuda, T., Shimizu, K., Tanimoto, M., and Kiura, K. (2011). Liposomal delivery of MicroRNA-7-expressing plasmid overcomes epidermal growth factor receptor tyrosine kinase inhibitor-resistance in lung cancer cells. Mol. Cancer Ther. *10*, 1720–1727.

45. Gomes-da-Silva, L.C., Fonseca, N.A., Moura, V., Pedroso de Lima, M.C., Simões, S., and Moreira, J.N. (2012). Lipid-based nanoparticles for siRNA delivery in cancer therapy: Paradigms and challenges. Acc. Chem. Res. *45*, 1163–1171.

46. Yu, Y., Yao, Y., Yan, H., Wang, R., Zhang, Z., Sun, X., Zhao, L., Ao, X., Xie, Z., and Wu, Q. (2016). A tumor-specific microRNA recognition system facilitates the accurate targeting to tumor cells by magnetic nanoparticles. Mol. Ther. Nucleic Acids *5*, e318.

47. Petrocca, F., and Lieberman, J. (2011). Promise and challenge of RNA interference-based therapy for cancer. J. Clin. Oncol. *29*, 747–754.

48. Castro, N.P., Fedorova-Abrams, N.D., Merchant, A.S., Rangel, M.C., Nagaoka, T., Karasawa, H., Klauzinska, M., Hewitt, S.M., Biswas, K., Sharan, S.K., and Salomon, D.S. (2015). Cripto-1 as a novel therapeutic target for triple negative breast cancer. Oncotarget *6*, 11910–11929.

49. Meza-Sosa, K.F., Pérez-García, E.I., Camacho-Concha, N., López-Gutiérrez, O., Pedraza-Alva, G., and Pérez-Martínez, L. (2014). MiR-7 promotes epithelial cell transformation by targeting the tumor suppressor KLF4. PLoS ONE *9*, e103987.

50. He, X., Li, C., Wu, X., and Yang, G. (2015). Docetaxel inhibits the proliferation of non-small-cell lung cancer cells via upregulation of microRNA-7 expression. Int. J. Clin. Exp. Pathol. *8*, 9072–9080.

51. Musacchio, T., and Torchilin, V.P. (2011). Recent developments in lipid-based pharmaceutical nanocarriers. Front. Biosci. (Landmark Ed.) *16*, 1388–1412.

52. Thanaketpaisarn, O., Nishikawa, M., Yamashita, F., and Hashida, M. (2005). Tissue-specific characteristics of in vivo electric gene: Transfer by tissue and intravenous injection of plasmid DNA. Pharm. Res. *22*, 883–891.

53. Mahato, R.I., Kawabata, K., Takakura, Y., and Hashida, M. (1995). In vivo disposition characteristics of plasmid DNA complexed with cationic liposomes. J. Drug Target. *3*, 149–157.

54. Tada, M., Hatano, E., Taura, K., Nitta, T., Koizumi, N., Ikai, I., and Shimahara, Y. (2006). High volume hydrodynamic injection of plasmid DNA via the hepatic artery results in a high level of gene expression in rat hepatocellular carcinoma induced by diethylnitrosamine. J. Gene Med. *8*, 1018–1026.

55. Naito, S., von Eschenbach, A.C., Giavazzi, R., and Fidler, I.J. (1986). Growth and metastasis of tumor cells isolated from a human renal cell carcinoma implanted into different organs of nude mice. Cancer Res. *46*, 4109–4115.

56. Zhao, J., Chen, C., Guo, M., Tao, Y., Cui, P., Zhou, Y., Qin, N., Zheng, J., Zhang, J., and Xu, L. (2016). MicroRNA-7 deficiency ameliorates the pathologies of acute lung injury through elevating KLF4. Front. Immunol. *7*, 389.

OMTN, Volume *6*

Supplemental Information

Targeted Expression of miR-7 Operated by TTF-1 Promoter Inhibited the Growth of Human Lung Cancer through the NDUFA4 Pathway

Liangyu Lei, Chao Chen, Juanjuan Zhao, HaiRong Wang, Mengmeng Guo, Ya Zhou, Junming Luo, Jidong Zhang, and Lin Xu

Material and methods

Construction of eukaryotic vector

The gene for the miR-7 (NM-407044) were expanded by PCR from human DNA derived from 95D cells using a forward primer (5′-CGACGCGTAAGAGAGAAATGAGCCACTTGC) and a reverse primer (5′-CCCAAGCTTCCTGCCACAGTGGGGGATG) and then subcloned into and *Mul I* and *HindIII* sites of pGL3.0 basic vector (Invitrogen Corp, San Diego, California, USA) to generate pGL3.0- basic-miR-7 vector (termed as p-miR-7). Afterwards, for the construction of the PGL3-basic-TTF-1-promoter-miR-7(termed as p-T-miR-7) vector, the promoter region of TTF-1 (NM-7080) was amplified from DNA derived from 95D cells using a forward primer (5′-CGGGGTACCTGTTTCGGCAACTAC) and a reverse primer (5′-CGACGCGTCCTTCT--GGGTCCTT) and subcloned into *Kpn*I and *Mul I* sites of p-miR-7 vector. Clone identity was verified using restriction digest analysis and plasmid DNA sequencing. Endotoxin-free plasmids were obtained using Endofree plasmid mega kit (QIAGEN GmbH, Hilden, Germany). Then, plasmids were transiently transferred into the 95D cells using Lipofectamine-2000 (Invitrogen) in different following experiments according to the manufacturer's instruction.

Real-time PCR assay

6 organs and tissues, including heart, liver, spleen, kidney, brain, and lymph nodes were collected from nude mice lung cancer model. DNAs were purified from 100 mg these indicated organs or tissues respectively. The primers were obtained from Shanghai Sangon Biological Engineering CO, and the other reagents were from TAKARA Bio Inc. Real-time PCR were performed according to the manufacturer's protocols. The following primers were used: forward:5'-AAGCCTCTTTTTCGTGGAAGT-3',reverse:5'-GGTTGGGCAGATTTTGAATG3'

Plasmid copies were quantified using the BIO-RAD CFX96 detection system (Bio-Rad Laboratories). Relative expression was calculated using the comparative threshold cycle (Ct) method. The copies of plasmid were calculated as previously description .[1,2]

Statistical analyses

The data were analyzed with GraphPad Prism 5.0 and were presented as the mean ± SD. Student's t-test was used when two conditions were compared. Probability values of <0.05 were considered significant; two-sided tests were performed.

Supplementary figure S1

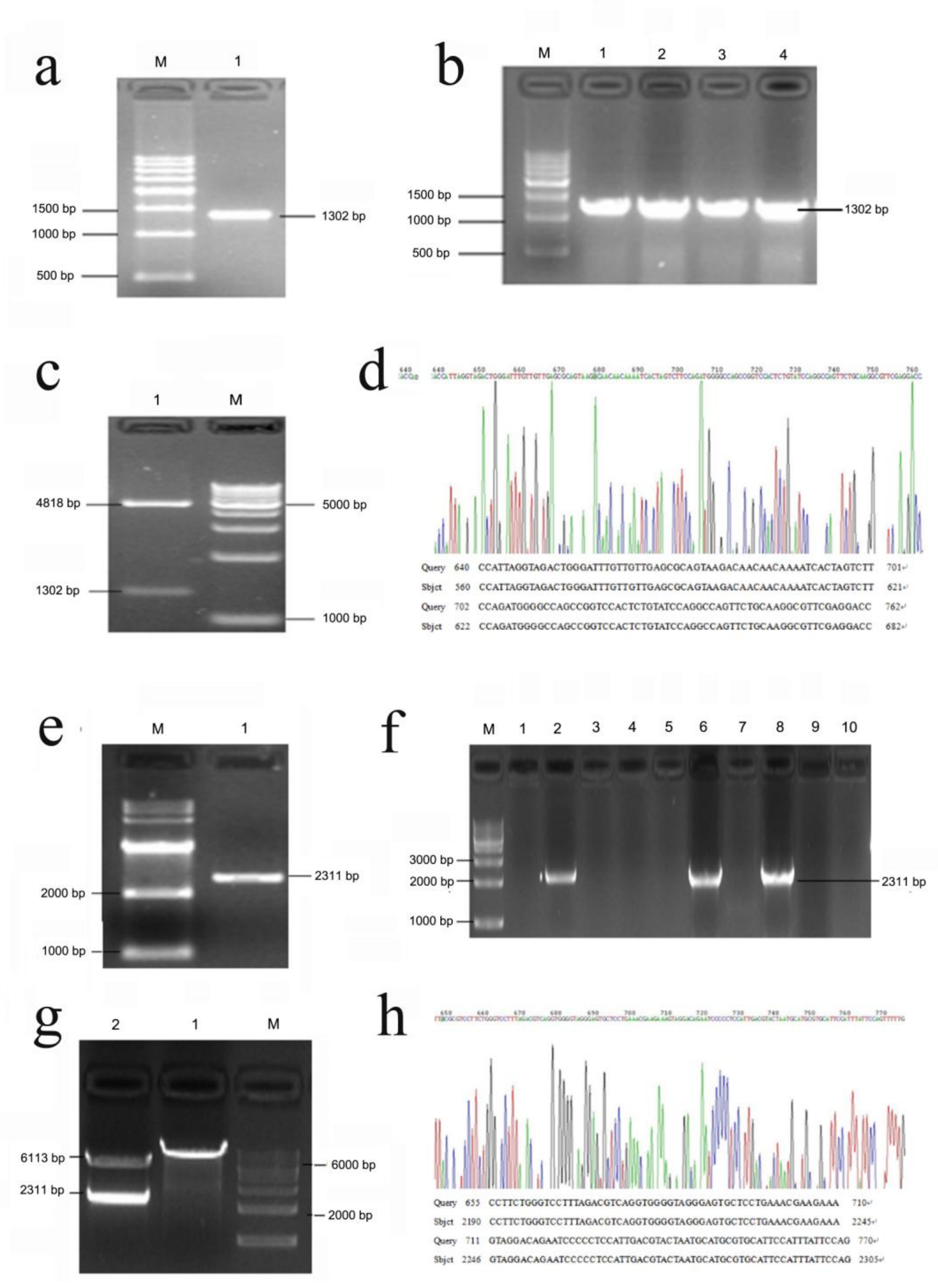

Supplement figure S1. The construction of plasmid p-T-miR-7.

miR-7 sequence were amplified from DNA derived from human lung cancer 95D cells by PCR, and then subcloned into pGL3.0 basic vector by restriction site of *Mul I* and *HindIII*. (**a**)The electrophoresis of pri-miR-7 (1302 bp) amplified from DNA by PCR. (**b**) The electrophoresis of PCR products of bacterium solution; 1-4: Electrophoresis of different PCR products; M: Marker; Identification of p-miR-7 plasmid by *Mul I* and *HindIII* double digestion(**c**) and sequencing (**d**). Then, 95D cells genomic DNA was used as template, TTF-1 promoter sequence was amplified by

PCR assay, and then cloned into p-miR-7 vector by restriction site of *Kpn I* and *Mul I* to construct PGL3.0-basic(-)-TTF-1-promoter-pri-miR-7(termed as p-T-miR-7). (**e**) The electrophoresis of PCR products of TTF-1 promoter (2311 bp) from the DNA. (**f**) The electrophoresis of PCR products of bacterium solution; 2,6,8: Electrophoresis of different PCR products; M: Marker; Identification of p-T-miR-7 plasmid by *Kpn I* and *Mul I* double digestion (**g**) and sequencing (**h**).

Supplementary figure S2

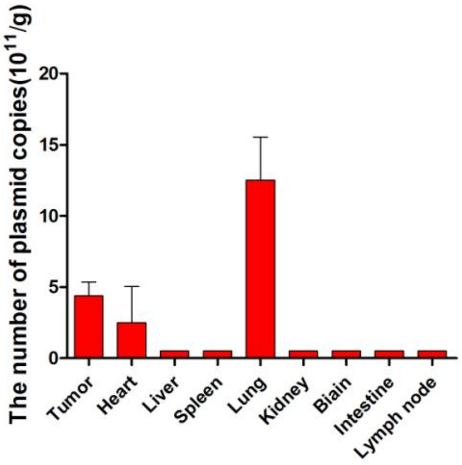

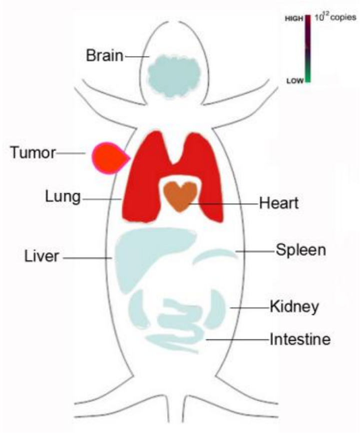

Supplement figure S2. The plasmid copy number in the main organs and tissues.

Human lung cancer cell line 95D cells were injected subcutaneously into right flank of Balb/c nude mice (n=8). 7 days later, the plasmid of p-T-miR-7(100mg) was remote given by subcutaneous injection into the left flank of nude five times every three days. 3 days after last injection, all of the mice were sacrificed and vital organs including heart, liver, spleen, kidney, brain and lymph node were harvested. The indicated DNA was purified and used for the calculation of plasmid copy number by Realtime PCR assay. (**a**) The data showed copies of plasmid in per gram organs or tissues. (**b**) The sketch map of distribution of plasmid.

Supplementary figure S3

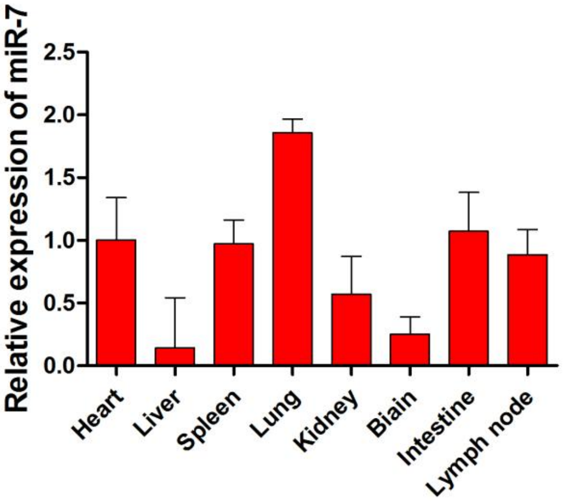

Supplement figure S3. The relative expression of miR-7.

Human lung cancer cell line 95D cells were injected subcutaneously into right flank of Balb/c nude mice (n=8). 7 days later, the plasmid of p-T-miR-7 (100mg) was remote given by subcutaneous injection into the left flank of nude five times every three days. 3 days after last injection, all of the mice were sacrificed and vital organs including heart, liver, spleen, kidney, brain and lymph node were harvested. Then, the relative expression level of miR-7 was analyzed by Realtime PCR assay. All data was normalized to heart.

Reference

[1] Ashty, S, Karim, Kathleen, A, Curran, Hal, S and Alper (2013). Characterization of plasmid burden and copy number in Saccharomyces cerevisiae for optimization of metabolic engineering applications. *FEMS Yeast Res* **13:**10.

[2]Yeon, Jeong, Son, Ae, Jin, Ryu, Ling, Li, Nam, Soo, Han and Ki, Jun, Jeong (2016). Development of a high-copy plasmid for enhanced production of recombinant proteins in Leuconostoc citreum. *Microb Cell Fact* **15**: 12.

Table S1. Over 4-fold down-regulation genes (534) in p-T-miR-7 injection group.

Trget gene	Fold change	Trget gene	Fold change	Trget gene	Fold change
RB1CC1	4.0004811	KIAA1244	4.3466776	FAM69A	5.0351474
ALDH7A1	4.0035322	lnc-CX3CR1-2	4.347025	ZCCHC6	5.0364891
AP1S1	4.0044539	MON2	4.3480297	GPAM	5.0369814
PAPOLG	4.004946	PALLD	4.3481575	RNA28S5	5.0640583
UEVLD	4.0079483	MAP9	4.3607582	GCLC	5.0918372
SLC39A1	4.0090386	C12orf29	4.3639462	GABPA	5.0993487
NMI	4.0091356	TXNDC9	4.3662175	CYP2U1	5.1121462
CD58	4.0093295	AKAP11	4.3671588	CRIM1	5.1124853
SLC39A8	4.0100366	MAP1B	4.376923	TIGD6	5.1163448
TMX1	4.0109195	PPAT	4.3782198	HAUS2	5.1170073
CBR1	4.0120512	MRPL12	4.3803404	TMEM14C	5.1240303
LOC100128593	4.0133117	PWWP2A	4.3838201	BDNF	5.1315089
TOP1	4.015285	RFK	4.3878679	MED21	5.1360545
ZFHX4-AS1	4.0230624	UQCRH	4.388114	NR3C1	5.146776
NAP1L4	4.0232364	TMEM206	4.3922516	FERMT1	5.1558826
DBF4	4.0236271	CNTD1	4.3927378	HBS1L	5.1585811
SGTB	4.0239367	ITFG1	4.3932113	AAED1	5.1615272
RNF213	4.0284339	CHCHD1	4.3948106	KPNA5	5.1650421
SAR1A	4.0286054	CAMLG	4.3951567	GALNT1	5.1791394
DNAJC8	4.0332463	CYCS	4.3995574	TUBB	5.1866571
DNAJC19	4.0386166	RPS15A	4.3998002	SEPT7	5.1869616
TERF1	4.0394778	RPS29	4.4044809	CCNT2	5.2006523
ANKRD46	4.0457504	YEATS4	4.4079015	UQCR11	5.2015659
SLC38A1	4.0486763	FAM169A	4.4106598	SIKE1	5.2101204
ZNF226	4.0527034	SHMT2	4.4131672	PRR3	5.2141027
BID	4.055145	SNRPG	4.4145549	MOB4	5.2148205
PDGFRL	4.0564638	FAM105A	4.4182045	TTK	5.2190166
SCAMP1	4.058141	PDIA3	4.424181	ADAT2	5.220592
SDHD	4.0639397	TAF13	4.4394101	LARP1B	5.2320448
IFIT5	4.0648252	SPCS2	4.4418464	XRCC6BP1	5.2335359
ALDH9A1	4.0701304	COPS8	4.442077	EPC1	5.2382358
CDH11	4.0715886	CLGN	4.4430941	PFN2	5.2403312
OSTF1	4.0752117	LIMA1	4.4503719	TPK1	5.2438936
YIPF5	4.0770612	ZBTB41	4.4625722	ASXL3	5.2496127
PHYHIPL	4.0779565	ARPP19	4.4644115	LAMP2	5.2499006
PDE12	4.0805881	MRPL14	4.4652285	TUBE1	5.2602505
CKS2	4.0824183	GGCX	4.4665874	ZNF396	5.2811016
LINC00597	4.0828569	SERPINB9	4.4700492	PTAR1	5.3097543
SNRPE	4.084074	DENND1B	4.4721761	PLA2G4A	5.3204708
MIR155HG	4.0867279	PLSCR4	4.4723237	SRP72	5.3227264
RNF2	4.0883735	NRIP1	4.4746592	MRPL13	5.3232193
DTD1	4.0924534	TMED2	4.476276	BID	5.3360282
C11orf58	4.0958997	MYCL	4.4764054	ARL5B	5.3383445
NCAM2	4.0968952	DPY19L2P2	4.4819607	GDI2	5.3388718
CDK1	4.0976521	TSNAX	4.4825066	KIF20B	5.3422926
NAA15	4.100482	TMEM126A	4.4842695	PLAC8	5.3463712
REEP3	4.1006181	PFDN4	4.4843814	FAM127C	5.3473199
ANG	4.101393	LINC00944	4.4935844	ALCAM	5.3509365
EAF2	4.101824	EPHA7	4.5006863	TMTC3	5.3733838
SETD7	4.1029706	GPR126	4.5015162	HSD17B11	5.3752394
ZNF254	4.1030846	COMMD10	4.5061302	MRPL12	5.3907821
KBTBD3	4.103441	PTGIS	4.5075934	TRUB1	5.3974826
PEX3	4.1053294	USP37	4.5104582	ANKRD31	5.4167143
IER3IP1	4.1110485	FUOM	4.5122925	DNM1L	5.425204
EPM2AIP1	4.1110499	HIAT1	4.5141489	OSTM1	5.4316605
GNPDA2	4.1119223	POLR1B	4.5143085	SNORA53	5.4380145
P2RY2	4.1133345	SLC7A11	4.5145901	C6orf57	5.4441899
HSP90AA1	4.1143628	NANP	4.5150971	KIF21A	5.4677868

Continued

Trget gene	Fold change	Trget gene	Fold change	Trget gene	Fold change
MCU	4.1149503	NDUFB6	4.5160548	PLS3	5.4695836
KIAA0825	4.1199685	LAMTOR3	4.5209263	BMPR2	5.4854744
RNU12	4.1214757	LRRIQ3	4.522189	SLC25A46	5.4916683
C10orf32	4.1260843	CASC5	4.5258802	RGS5	5.5015849
SLC35E3	4.1271017	SPECC1	4.5363288	AREG	5.502504
RAB8B	4.1273489	NUP98	4.5376596	LOH12CR1	5.5064992
HACL1	4.1274078	DR1	4.5404356	CCDC132	5.5348047
PIGP	4.1293554	KLHL7	4.5413631	TOR1AIP2	5.5424099
ZNF678	4.1308965	CENPA	4.5511199	NUP37	5.5868396
SLC16A1	4.132257	ENPP1	4.5550251	PJA2	5.5926227
TAB3	4.1323418	GALC	4.5575737	NUDT14	5.5959951
NDUFA4	4.1337181	ISOC1	4.5584078	SEL1L	5.6111108
RPS15A	4.1353258	SNORA23	4.5589766	RBM43	5.6274499
NDUFA11	4.141982	GSKIP	4.5606675	SLC31A1	5.6392426
KCTD12	4.1436553	SEC24D	4.5629603	MLF1	5.6467509
METTL21B	4.1471792	CASP8	4.568781	NT5DC1	5.6498732
SELT	4.1473184	KRIT1	4.5727226	GPR22	5.6614947
ZNF91	4.1491804	CMC1	4.5761806	MCTP1	5.6621442
CRYZ	4.1508661	PPIL1	4.5788554	SLC10A4	5.6690017
HSPA13	4.1542931	PIGK	4.5803537	HAVCR2	5.6755519
CTBS	4.1547962	CLDN1	4.581701	SHROOM3	5.6941914
THAP2	4.1576396	LYPLA1	4.5866938	TMED7-TICAM2	5.6985703
CCNB1	4.1589144	ST8SIA4	4.6039941	TMEM14A	5.7072292
DNAJC19	4.1597246	SLC25A46	4.6074257	OTUD6B	5.7202992
CANX	4.159835	BAG2	4.6101702	NAT1	5.7249199
CACYBP	4.1659427	SELT	4.6131609	CAP2	5.7308741
NPAT	4.1670332	NXT2	4.6160697	NLGN1	5.7317342
TCF12	4.1672539	UFM1	4.6193697	ANTXR1	5.7548128
BMPR1A	4.1708968	PRNP	4.6195586	RPL26L1	5.8384402
ESD	4.1715513	MDH1	4.6213809	RRM1	5.8552388
STRN	4.1770621	B9D1	4.6237785	GATC	5.8761198
GPR180	4.1788228	SMAD1	4.6240196	NQO1	5.9320905
RPL39	4.1791311	DSG2	4.6247424	HSPA4L	5.9581465
DHCR24	4.179696	P2RY1	4.6274686	C17orf104	5.9605328
RPL13AP3	4.1799596	ARL6IP5	4.6354757	HSD17B7	5.9710183
COX16	4.1827797	CRYZ	4.6441449	DBF4	5.9711909
TMEM123	4.183914	E2F7	4.64554	CKS1B	5.983232
SPRYD7	4.186569	DPH3	4.6482511	BBS10	6.0043955
TSPAN12	4.1873172	FLJ40536	4.6486787	KBTBD7	6.0282239
RBBP4	4.1899761	SNRPE	4.6501975	ZNF705A	6.0626553
LOC389831	4.193213	PHACTR2	4.6504119	MRPL42	6.0695729
TFPI	4.1939812	SPOCK3	4.6589337	FSIP2	6.0978772
TMTC2	4.1949058	PTPLAD1	4.665446	ATP5C1	6.1184024
SAP30	4.2004256	CHMP5	4.6710449	ATP7A	6.1240379
PCDHB9	4.2031674	SELM	4.675796	PIGK	6.1493874
CCDC186	4.2054809	TMLHE	4.6761182	TMEM38B	6.1752652
DDB1	4.2067235	PYROXD1	4.6780533	GLS	6.17571
LCOR	4.2068361	TMED5	4.6804946	SNORA59B	6.2214433
HLTF	4.2111667	TMEM14C	4.6810624	MRPS14	6.2243794
QSER1	4.2112339	PHF5A	4.6833621	FAHD1	6.2329166
TERF1	4.2162763	LAMTOR5	4.6841166	CHRNA5	6.2700446
SENP7	4.218407	SQRDL	4.6841426	ATP7A	6.2799497
IL6ST	4.2202311	TP53	4.6842024	WDFY3	6.29795
DACH1	4.2213861	LSM3	4.6865556	HHEX	6.3054372
EMB	4.2226185	CCDC121	4.689615	POMP	6.3258178
CLN5	4.2234501	DEF8	4.6917625	ESCO2	6.3962361
UTY	4.2242037	IL7	4.7021645	ARHGAP18	6.3977814
PSMA4	4.2264928	EPS8	4.7160228	ACSL3	6.4274055
SPIN4	4.2290218	C2orf88	4.7167682	GOLT1B	6.4374334
SLC39A9	4.2296934	COX5A	4.7227494	PPM1A	6.4660022

Continued

Trget gene	Fold change	Trget gene	Fold change	Trget gene	Fold change
TROVE2	4.2302414	PLAA	4.7274638	CSRNP1	6.480614
FLJ10038	4.2308748	SNRPE	4.7282293	TRMT1L	6.4819729
UBLCP1	4.2348416	HOMER1	4.7323212	SGOL2	6.4859235
LEPR	4.2356671	SEP15	4.7327345	PLCB1	6.5323469
SMIM11	4.2359689	CHD9	4.7368162	CTSO	6.5413568
INPP4B	4.2373001	NDUFA13	4.7436218	SLC39A10	6.5608808
CLIC4	4.2375494	NID2	4.7440493	TMEM14B	6.5609558
LINC01503	4.2381778	DLEU2L	4.7860352	AHR	6.5813781
LYPLAL1	4.2397538	FKBP14	4.7885438	TMEM106B	6.5843421
FCF1	4.2402255	KCNG3	4.7889504	EDEM3	6.6796888
ZNF720	4.2442855	MREG	4.7917764	C8orf88	6.7013572
TP53TG1	4.2477139	CROT	4.7921896	CPE	6.7260197
STXBP4	4.2496647	COMMD8	4.8076684	BPNT1	6.82355
ATXN10	4.2500214	PPM1L	4.8147277	DNAJC6	6.8529302
PHYH	4.2522093	OSBPL8	4.8157033	ERAP1	6.8606582
C2orf73	4.2527894	IL6ST	4.8157941	LMBR1	6.9024953
AARD	4.2531042	MRPL54	4.8167642	LEPROT	6.9424631
ZDHHC20	4.254398	SLC30A6	4.8176884	CHMP2B	7.0466424
ARSK	4.255253	CTDSPL2	4.8195929	MRPL1	7.0622143
GABARAPL1	4.2564468	MDH1	4.8278505	GULP1	7.073793
ITGB3	4.2582156	WDFY1	4.8299183	TMTC3	7.0817602
HOXA13	4.2586643	SDHD	4.8306174	TMPO	7.1223632
TM2D1	4.2597374	ELMOD2	4.8370351	SULT2B1	7.1726145
PAK2	4.2599072	GSTO1	4.8399797	ITGA4	7.268674
TSPAN14	4.2609969	FNIP1	4.8441314	PLS1	7.2696419
PIGN	4.2615297	COL8A1	4.8548795	NUDCD1	7.3693532
GABPB1	4.263449	POT1	4.8595917	C11orf58	7.4399344
HNRNPCL1	4.2655359	GULP1	4.8646287	CHCHD2	7.4777539
BIK	4.2656352	GSR	4.8659598	CAPN7	7.5704807
ITGAV	4.2692422	PTN	4.8729043	TRMT10A	7.5948838
POLR3G	4.2712964	SEC23A	4.8737694	MANEA	7.7068136
UQCRH	4.2724452	C15orf48	4.8746208	BFSP2	7.7398328
TIPARP	4.2737911	C11orf70	4.8765069	CNRIP1	8.1045889
SFMBT2	4.2762571	LINC01300	4.8774409	LRP12	8.1352462
HS2ST1	4.2815999	MRPS28	4.8954518	FGF12	8.1820253
SPINT2	4.2816655	CNIH1	4.8976681	RBM24	8.2989781
HIST1H4A	4.2816712	MGST3	4.9045882	FZD7	8.3067556
C2orf76	4.2841254	UMAD1	4.9051559	PRKACB	8.3318271
CERS6	4.2866006	CMTM6	4.9155563	TMEM17	8.383965
CASP3	4.2956129	TUBB	4.9252829	PHF14	8.3991539
SNRPG	4.2985855	FBXO22	4.9300323	EDIL3	8.6604477
SSX2IP	4.300463	TMEM56	4.9309205	TMEM106B	8.8020953
DTWD1	4.3004934	HOOK3	4.9451459	TLE4	8.8432238
ARL6IP1	4.3044443	DPH6	4.9558403	ACAP2	8.9732276
DLEU2L	4.3053335	HINT1	4.9558448	ATP6V1C2	8.9737867
PGM2	4.3055126	LOC100131048	4.9653899	FAM171B	9.0195703
NAPEPLD	4.3063692	LZTFL1	4.9710741	AKAP9	9.4973986
TMEM135	4.3069811	EIF5A2	4.9745703	RBBP9	9.5211247
PDLIM3	4.307655	C1QBP	4.9775679	PIGM	9.6391839
COMMD6	4.3084771	DUSP19	4.9913532	TMSB15A	9.6602271
PARVA	4.3094845	PRRC1	4.9961899	FAM198B	9.6818188
ECHDC1	4.3106248	RPA3	5.0036886	SLC30A7	9.805271
GPX8	4.315794	JAZF1	5.00808	GTF3C3	10.02813
ZC3HAV1	4.3208603	METTL20	5.0131701	DNAJB9	10.1163125
VPS41	4.3261302	SNRPG	5.015446	FSTL5	10.1447092
PCYOX1	4.3275065	SIVA1	5.0180401	CDC73	11.4744763
ZHX1	4.3312805	ARHGAP5	5.0198237	C17orf104	11.9460056
C3orf58	4.3333552	LAMP3	5.0213473	TMEM38B	13.1177293
ANLN	4.339484	DEPDC1	5.0233208	XK	15.5924897
TMEM19	4.3415554	CD302	5.0273091	HIST1H4C	15.6153013
